# ADAMTS12 acts as a tumor microenvironment related cancer promoter in gastric cancer

**DOI:** 10.1038/s41598-021-90330-3

**Published:** 2021-05-26

**Authors:** Yangming Hou, Yingjuan Xu, Dequan Wu

**Affiliations:** 1 Department of Hepatic Surgery, The Second Affiliated Hospital of Harbin Medical University, No. 246 Xuefu Avenue, Harbin, 150086 Heilongjiang China; 2 Department of Obstetrics and Gynecology, China-Japan Union Hospital, Jilin University, No. 126 Xiantai Avenue, Changchun, 130033 China

**Keywords:** Gastric cancer, Cancer epigenetics

## Abstract

The infiltration degree of immune and stromal cells has been shown clinically significant in tumor microenvironment (TME). However, the utility of stromal and immune components in Gastric cancer (GC) has not been investigated in detail. In the present study, ESTIMATE and CIBERSORT algorithms were applied to calculate the immune/stromal scores and the proportion of tumor-infiltrating immune cell (TIC) in GC cohort, including 415 cases from The Cancer Genome Atlas (TCGA) database. The differentially expressed genes (DEGs) were screened by Cox proportional hazard regression analysis and protein–protein interaction (PPI) network construction. Then ADAMTS12 was regarded as one of the most predictive factors. Further analysis showed that ADAMTS12 expression was significantly higher in tumor samples and correlated with poor prognosis. Gene Set Enrichment Analysis (GSEA) indicated that in high ADAMTS12 expression group gene sets were mainly enriched in cancer and immune-related activities. In the low ADAMTS12 expression group, the genes were enriched in the oxidative phosphorylation pathway. CIBERSORT analysis for the proportion of TICs revealed that ADAMTS12 expression was positively correlated with Macrophages M0/M1/M2 and negatively correlated with T cells follicular helper. Therefore, ADAMTS12 might be a tumor promoter and responsible for TME status and tumor energy metabolic conversion.

## Introduction

According to the latest global cancer epidemic statistics (GLOBOCAN), gastric cancer (GC) is the third leading cause of cancer related death worldwide^1,2^. GC patients are frequently diagnosed at advanced stage with the five-year survival rate less than 20%^3^. To better estimate the impacts of genetic composition on tumor clinical prognosis, comprehensive data sets of genome-wide gene expression collections including The Cancer Genome Atlas (TCGA) have been established to detect and classify genomic abnormalities in malignant tumor cohorts^4,5^.

Internal genes, especially major transcription factors conduct the occurrence, development and evolution of malignancies. Tumor microenvironment (TME) has also been reported to regulate the expression of tumor-associated genes, hence the clinical outcomes^6–11^. TME is the cellular milieu where tumor located. It is composed of non-malignant cellular elements including mesenchymal cells, immune cells, endothelial cells, inflammatory mediators and extracellular matrix (ECM) molecules^12,13^. In TME, immune and stromal cells are two major components, and have been supposed to be crucial for tumor diagnosis and prognostic assessment^14^. To predict the purity of tumor tissues, Yoshihara et al.^9^ had created an algorithm mode called ESTIMATE (Estimation of STromal and Immune cells in MAlignant Tumor tissues using Expression data), basing on gene expression data from the TCGA database. By analyzing the specific gene expression characteristics of immune cells and mesenchymal cells, the algorithm would calculate immune and stromal scores and predict the infiltration of non-tumor cells. It has already been applied to prostate cancer^15^, breast cancer^16^, colon cancer^17^ and glioblastoma^18^ demonstrated the effectiveness of this big data-based algorithm. However, the utility of ESTIMATE in GC has not been investigated in detail.

In the current work, by taking use of both ESTIMATE algorithm and CIBERSORT computational methods, based on TCGA database, for the first time, we extracted a set of microenvironment-related genes, which could predict poor prognosis in GC patients. ADAMTS12 belongs to a member of the ADAMTS (a disintegrin and metalloproteinase with thrombospondin motifs) protein family. The protease family is a set of extracellular multifunctional enzymes that play a crucial role in cell-extracellular matrix interactions^19^. By far, ADAMTS metalloproteinases have been proved closely related to the oncogenesis and development^20^. In cancer-related processes, ADAMTS12 shows dual effects of pro and/or anti-tumor in a proteolytic or non-proteolytic manner. Here, by further comparing differentially expressed genes (DEGs), we confirmed that ADAMTS12 could be a potential prognostic factor and might be responsible for the change of TME status in GC.

## Results

### Immune scores and stromal scores are significantly associated with clinicopathological parameters and prognosis of GC

**Figure 1:**
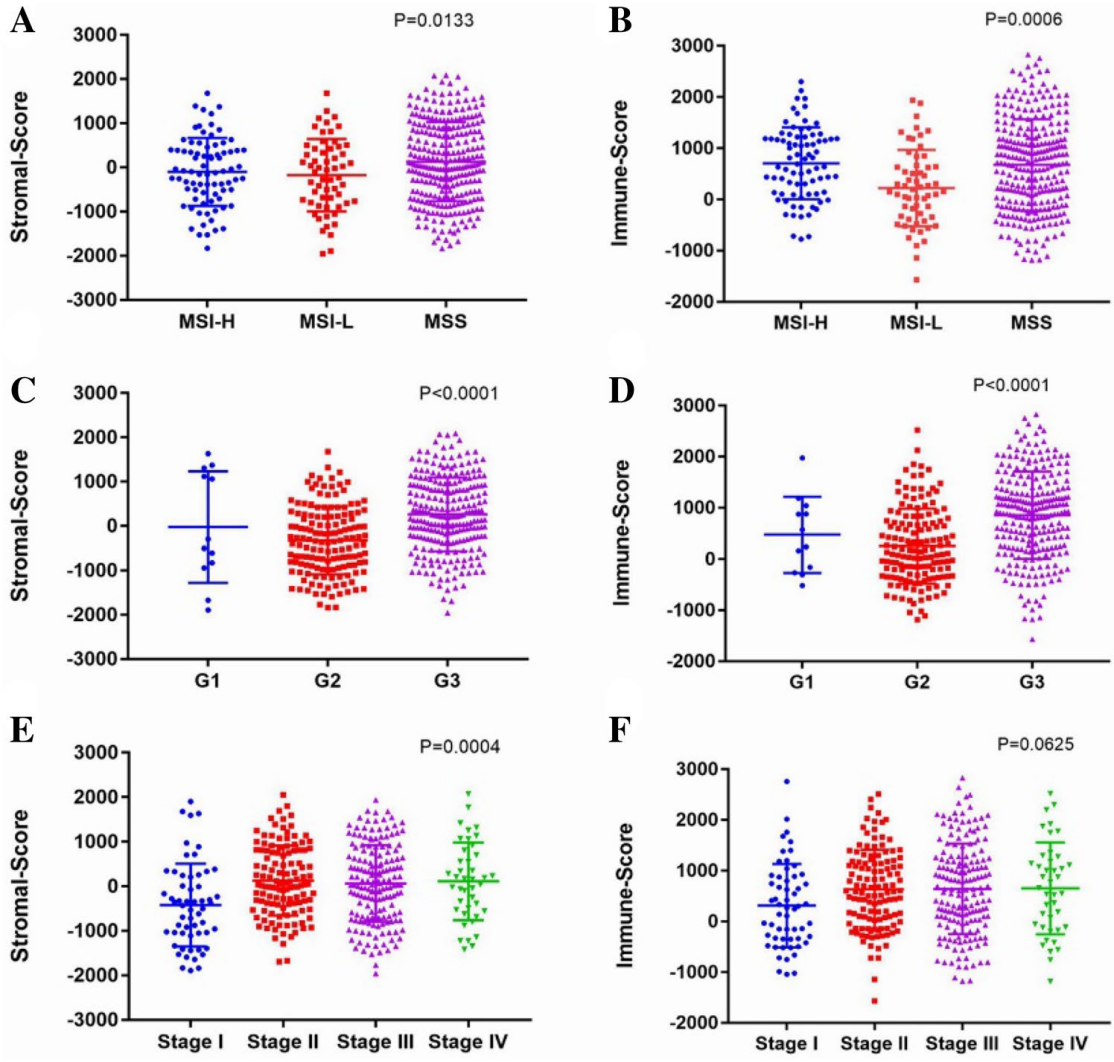
Stromal scores and immune scores are associated with clinicopathological parameters of GC cases. (**A**) The distribution of stromal scores in GC subtypes, scatter plot indicates a significant correlation between GC subtypes and stromal scores (n = 414, *p* = 0.0133). (**B**) The distribution of immune scores in GC subtypes, scatter plot indicates a significant correlation between GC subtypes and immune scores (n = 414, *p* = 0.0006). (**C**) The distribution of stromal scores in GC histological grades, scatter plot indicates a significant correlation between GC histological grades and stromal scores (n = 406, *p* < 0.0001). (**D**) The distribution of immune scores in GC histological grades, scatter plot indicates a significant correlation between GC histological grades and immune scores (n = 406, *p* < 0.0001). (**E**) The distribution of stromal scores in GC clinical stages, scatter plot indicates a significant correlation between GC clinical stages and stromal scores (n = 392, *p* = 0.0004). (**F**) The distribution of immune scores in GC clinical stages, scatter plot indicates a significant correlation between GC clinical stages and immune scores (n = 392, *p* = 0.0625).

We downloaded the gene expression profiles and clinical data of all 415 GC patients from TCGA database with primary pathological diagnosis made between 1996 and 2013. Among them, 147 (35.4%) cases were female and 268 (64.6%) cases were male. According to the number and type of MS loci, all cases were divided into 79 (19.0%) MSI-H subtype, 59 (14.2%) MSI-L subtype, and 276 (66.5%) cases of MSS subtype, 1 patient was of unknown type. Based on the ESTIMATE algorithm, the immune scores distributed from − 1184.83 to 2826.73 and the stromal scores ranged from − 1838.38 to 2085.81. The rank order of stromal scores across GC subtypes from highest to lowest was MSS > MSI-H > MSI-L (Fig. 1A, p = 0.0133). The average immune score of MSI-H subtype cases ranked the highest of all 3 subtypes, followed by that of MSS, and MSI-L subtype (Fig. 1B, p = 0.0006). The above results indicated that the immune score and stromal score were both meaningful in the relevance of subtype classification. In addition, we analyzed the scores in histological grade and clinical stage respectively, demonstrating that the stromal score (*p* < 0.0001) and immune score (*p* < 0.0001) were all associated with histological grade (Fig. 1C,D). As for clinical stage, the stromal score was statistically significant (*p* = 0.0004), but the immune score (*p* = 0.0625) just showed a relevant trend (Fig. 1E,F).

**Figure 2:**
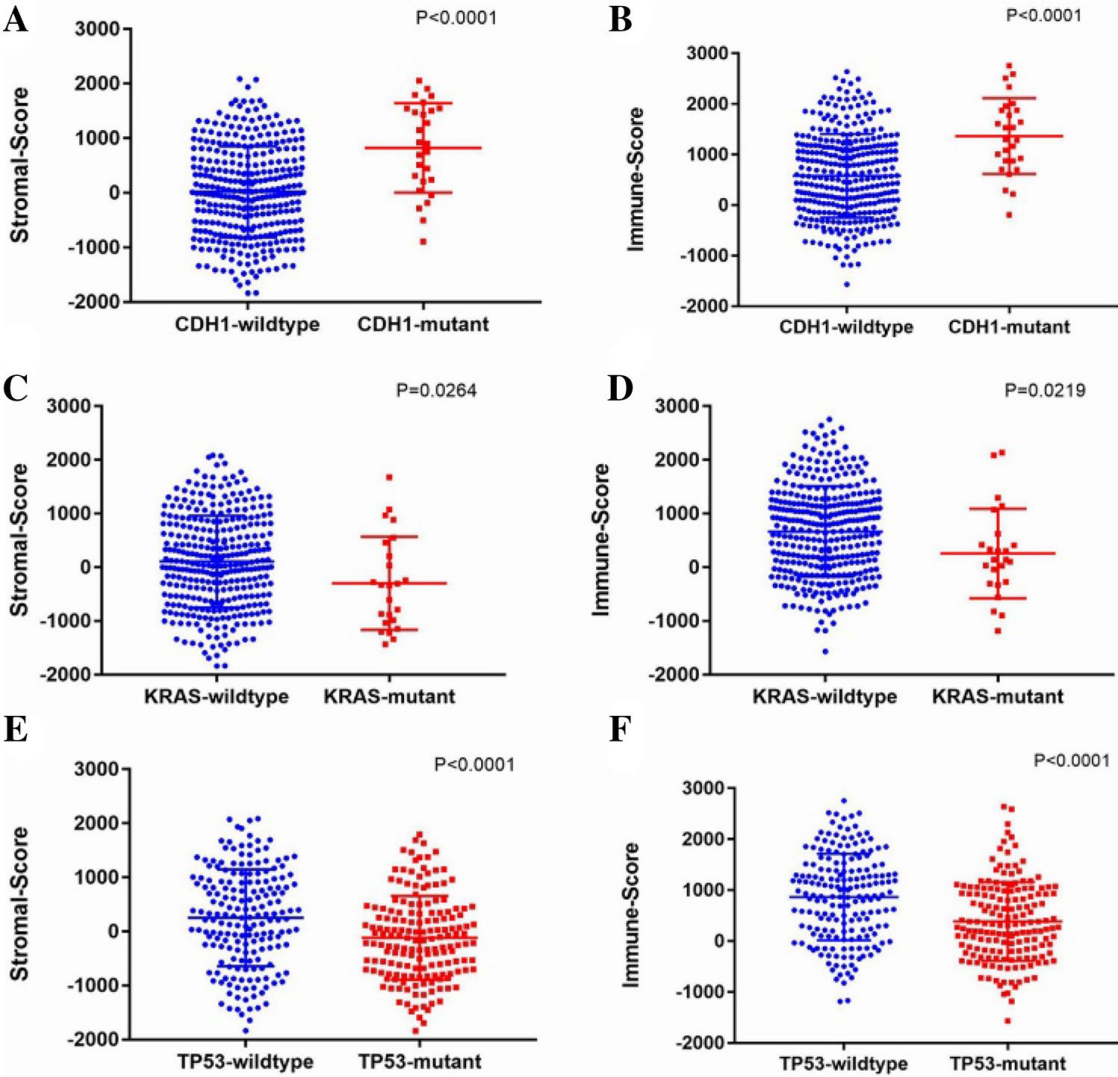
Immune scores and stromal scores are associated with some prognostic gene mutation of GC cases. (**A**) The distribution of stromal scores in CDH1 mutants and CDH1 wild-type GC cases, scatter plot indicates a significant correlation between GC CDH1 mutation status and stromal scores (n = 356, *p* < 0.0001). (**B**) The distribution of immune scores in CDH1 mutants and CDH1 wild-type GC cases, scatter plot indicates a significant correlation between GC CDH1 mutation status and immune scores (n = 356, *p* < 0.0001). (**C**) The distribution of stromal scores in KRAS mutants and KRAS wild-type GC cases, scatter plot indicates a significant correlation between GC KRAS mutation status and stromal scores (n = 356, *p* = 0.0264). (**D**) The distribution of immune scores in KRAS mutants and KRAS wild-type GC cases, scatter plot indicates a significant correlation between GC KRAS mutation status and immune scores (n = 356, *p* = 0.0219). (**E**) The distribution of stromal scores in TP53 mutants and TP53 wild-type GC cases, scatter plot indicates a significant correlation between GC TP53 mutation status and stromal scores (n = 356, *p* < 0.0001). (**F**) The distribution of immune scores in TP53 mutants and TP53 wild-type GC cases, scatter plot indicates a significant correlation between GC TP53 mutation status and immune scores (n = 356, *p* < 0.0001).

Certain gene mutation could predict the prognosis of GC patients^21–24^. We mapped the distribution of the immune and stromal scores according to CDH1, TP53 and KRAS gene mutation. The statistical analysis showed that CDH1 mutant cases had higher stromal (*p* < 0.0001) and immune (*p* < 0.0001) scores (Fig. 2A,B). On the other hand, patients with TP53 or KRAS mutants enjoyed lower stromal and immune scores (Fig. 2C–F).

To investigate the potential clinical utility of immune and stromal scores, log-rank test and univariate Cox regression model were applied. Moreover, we excluded cases with overall survival (OS) less than 70 days to avert the impact of non-tumor factors. All 301 GC cases with complete clinical data were split into two groups according to optimal cutoff values determined by X-tile software (for stromal score, low score group: − 1957.2–383.12, 103 cases, and high score group: − 379.61–2053.43, 198 cases; for immune score, low score group: − 1184.83– − 331.49, 47 cases, and high score group: − 329.95–2826.73, 254 cases). It was identified that the cases with high stromal score had shorter overall survival (*p* = 0.010; Table 1). In addition, age (*p* = 0.033), TNM stage (*p* < 0.001) and lymph node metastasis (*p* < 0.001) were also combined with GC prognosis. Parameters identified as significant in the univariate analysis and some important clinicopathological factors were included in the multivariate Cox regression model. The result indicated that stromal score (*p* = 0.029, Table 2) could serve as an independent prognostic marker for OS. Furthermore, age (*p* = 0.011) and TNM stage (*p* < 0.001) were also significant. However, immune score (*p* = 0.646), MS type (*p* = 0.074), lymph node metastasis (*p* = 0.246) and histological grade (*p* = 0.173) were not independent predictive factors for GC patients.

**Table 1:** Univariate survival analysis of OS in patients with gastric cancer.

Variable	No	OS, days		*p*-value
		Mean ± SE	95%CI	
**Age, years**	379			**0.033**
> 65	202	1369.532 ± 152.495	1070.641–1668.423	
≤ 65	177	2015.810 ± 190.318	1642.786–2388.834	
**Gender**	382			
Female	131	1903.202 ± 180.150	1550.109–2256.296	0.448
Male	251	1604.595 ± 157.902	1295.108–1914.083	
**MS type**	382			0.096
MSI-H	70	1827.188 ± 212.381	1410.921–2243.456	
MSI-L/MSS	312	1623.222 ± 151.025	1327.213–1919.231	
**Histological grade**	373			0.117
G1/G2	147	1355.044 ± 91.937	1174.848–1535.240	
G3	226	1773.580 ± 157.647	1464.592–2082.567	
**TNM stage**	369			** < 0.001**
I/II	169	2148.074 ± 198.572	1758.873–2537.274	
III/IV	200	1455.257 ± 162.135	1137.472–1773.043	
**Lymph node metastasis**	341			** < 0.001**
No	94	2295.069 ± 243.166	1818.464–2771.674	
Yes	249	1566.092 ± 155.084	1262.127–1870.057	
Anatomic subdivision	365			0.500
Antrum/distal	138	1463.279 ± 182.070	1106.422–1820.136	
Cardia/proximal	55	1353.763 ± 239.020	885.283–822.243	
Fundus/body	132	1978.355 ± 219.488	1548.158–2408.551	
Gastroesophageal junction	40	1327.102 ± 137.918	1056.783–1597.421	
**Pylori infection**	176			0.286
No	156	1324.090 ± 87.932	1151.743–1496.436	
Yes	20	2229.721 ± 443.254	1360.944–3098.498	
**Family history**	302			0.667
No	285	1846.560 ± 147.032	1558.377–2134.742	
Yes	17	1398.167 ± 385.644	642.304–2154.029	
**Stromal score**	358			**0.010**
Low	103	1536.173 ± 103.066	1334.163–1738.183	
High	198	1539.045 ± 174.518	1196.990–1881.099	
**Immune score**	301			0.139
Low	47	1557.399 ± 145.214	1272.779–1842.019	
High	254	1667.111 ± 157.335	1358.733–1975.488	

The bold indicated the result statistically significant.

**Table 2:** Multivariate Cox regression analysis for various potential prognostic characteristics of OS in 301 patients with gastric cancer.

Variable	Exp(B)	OS	
		95%CI	*p*-value
Stromal score	1.589	1.049–2.408	**0.029**
Immune score	1.155	0.625–2.132	0.646
MS type	1.589	0.957–2.641	0.074
Lymph node metastasis	1.447	0.776–2.698	0.246
Histological grade	1.339	0.880–2.035	0.173
Age	1.023	1.005–1.042	**0.011**
TNM stage	2.285	1.533–3.405	** < 0.001**

The bold indicated the result statistically significant.

### Identification of DEGs with stromal score in GC

**Figure 3:**
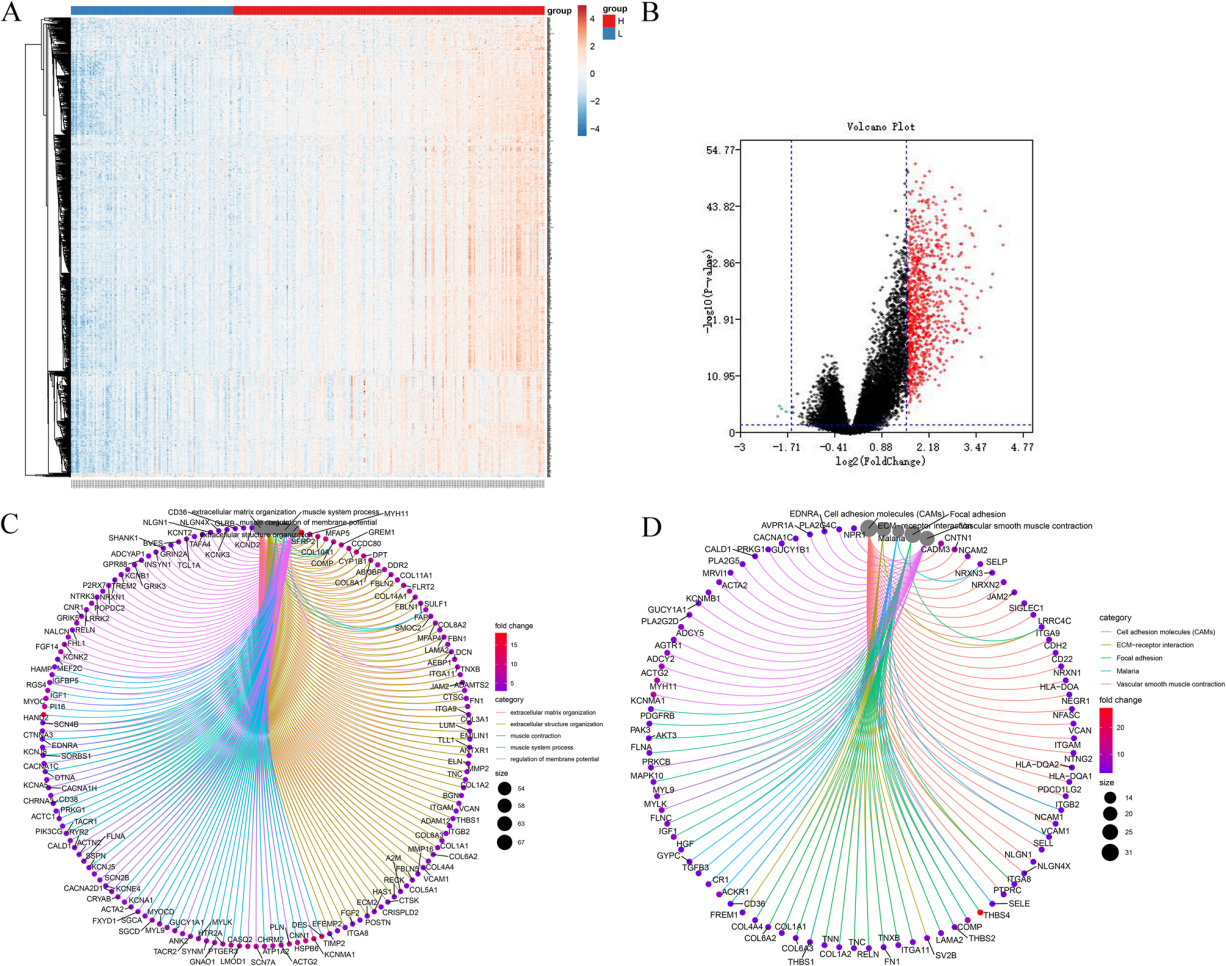
Gene differential expression profiles with stromal scores in GC cases. (**A**) Heatmap of the DEGs in high versus low stromal score groups (fold change > 3, *p* < 0.01). (**B**) Volcano plot of the DEGs in high versus low stromal score groups (fold change > 3, *p* < 0.01). (**C**) GO enrichment analysis of 834 DEGs (The cutoff value was set as *p* and q < 0.05). (**D**) KEGG enrichment analysis of 834 DEGs (*p* and q < 0.05).

To demonstrate the relationship of gene differential expression profiles with stromal score, we compared the RNA-seq data for all 301 GC cases. They were divided into two groups according to the optimal cutoff value determined by X-tile software (low score group: − 1957.2–383.12, 103 cases, and high score group: − 379.61–2053.43, 198 cases). Intuitive gene expression profiles of cases derived from high versus low stromal scores cohorts were shown in the Heat map and volcano plot of Fig. 3A,B. The high stromal score group had 834 genes up-regulated and 5 genes down-regulated (fold change > 3, *p* < 0.05). Therefore, we decided to use these up-regulated differentially expressed Genes (DEGs) as the focus for subsequent analysis in this manuscript (Supplementary Table 1).

In order to outline the potential mechanism of DEGs, we used the R language with the packages of cluster Profiler, enrichplot and ggplot2 to perform functional enrichment analysis. Gene ontology (GO) analysis included three functional groups: molecular function, biological processes, and cell composition. Results indicated that most significant features are mapped to TME-related GO terms, including extracellular matrix organization, immunoglobulin binding and cytokine binding Fig. 3C. By Kyoto Encyclopedia of Genes and Genomes (KEGG)^25^ enrichment analysis, up-regulated DEGs were enriched in Cell adhesion molecules (CAMs), PI3K − Akt signaling pathway, ECM − receptor interaction, B cell receptor signaling pathway, focal adhesion et al. Figure 3D. Therefore, the overall function of DEGs seems to be mapped on immune-related activities.

### Correlation between individual DEGs expression and overall survival with univariate Cox regression

To investigate whether each individual DEG had an effect on overall survival, we created Cox proportional hazards regression model based on TCGA database. Among the 834 upregulated DEGs in high-scoring group, a total of 501 DEGs (Supplementary Table 2) were associated with poor overall survival (*p* < 0.05).

In order to identify whether the candidate DEGs also had significant prognostic value in additional GC cases, we downloaded the microarray datasets GSE26253^26^ which included up to 432 GC cases from Gene Expression Omnibus (GEO) database. A total of 377 screened DEGs could be obtained at GSE26253. By creating the Cox proportional hazards regression model, a total of 81 DEGs (Supplementary Table 3) were confirmed to be significantly associated with recurrence-free survival (RFS).

### PPI network construction and module analysis among genes with prognostic value

**Figure 4:**
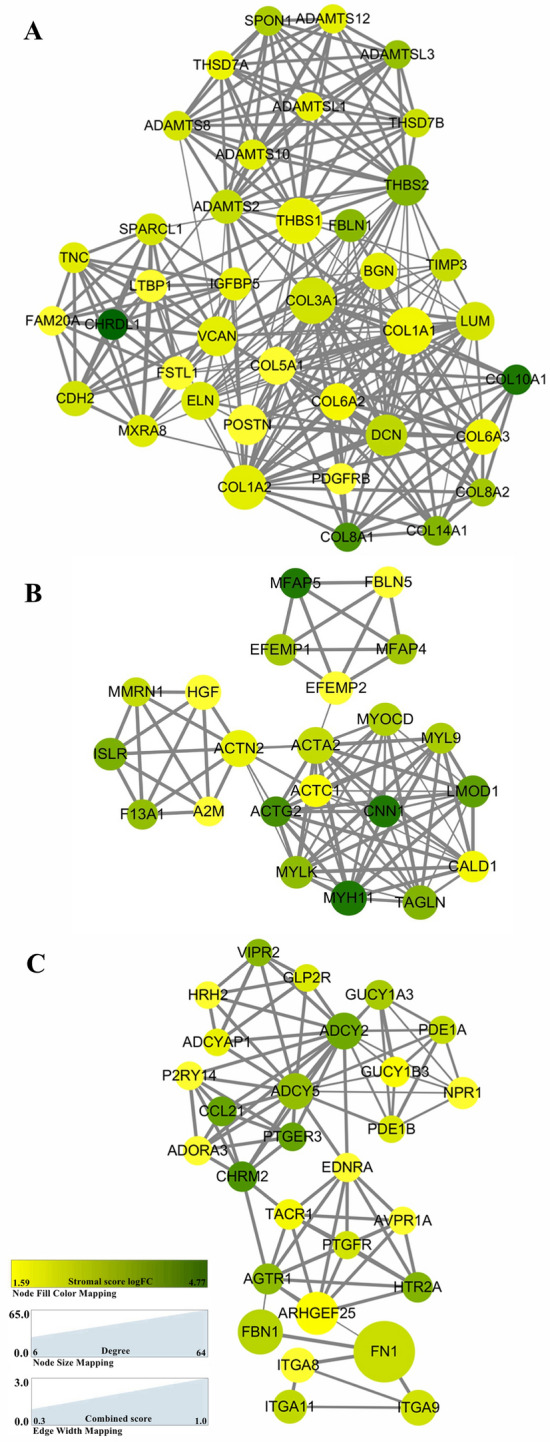
Top 3 PPI networks of COL1A1, ACTA2 and ADCY5 modules. The color of the nodes in the PPI network represents the log (FC) value of the gene expression in stromal score groups. The size of nodes reflects the number of interacting proteins with the designated ones. The edge width indicates the combined scores obtained from STRING. The figure was initially constructed by Cytoscape (v3.6.1) and screened out the most significant modules using plug-in Molecular Complex Detection (MCODE) (version 1.4.2).

To better illustrate the interactions among screened DEGs, we constructed protein–protein interaction (PPI) networks by Cytoscape (v3.6.1)^27^ and screened out the most significant modules using plug-in Molecular Complex Detection (MCODE) (version 1.4.2)^28^ which acted as a clustering tool was used to identify densely connected network regions (Fig. 4). After excluding the insular or rarely connected nodes, a network of DEGs was established, which made up of 10 modules, including 390 nodes and 1322 edges. The top three modules were selected for further analysis and named as COL1A1, ACTA2 and ADCY5. COL1A1 module (Fig. 4A) was constituted by 259 edges and 39 nodes, the MCODE score of all factors was more than 9 points. In addition, DEGs including ADAMTS12, ADAMTS10, ADAMTSL1, ADAMTS2, ADAMTSL3, SPON1, COL14A1, ELN, TNC, FBLN1, THBS1, LTBP1, TIMP3, COL10A1, ADAMTS8, POSTN, LUM, BGN, DCN, COL1A1, COL3A1, VCAN, COL1A2, COL5A1, COL6A2, COL8A2, COL6A3, SPARCL1, COL8A1 were closely related to the extracellular matrix. In the ACTA2 module (Fig. 4B), the core was occupied by ACTN2 and ACTA2. For the ADCY5 module (Fig. 4C), FN1, FBN1, ADCY2, ADCY5 and ITGA9 were considered as hub nodes with higher degree values.

The nodes in three modules were brought into the multivariate Cox regression model respectively. Statistical results showed that ADAMTS12, THSD7A and CDH2 could be used as independent prognostic genes of overall survival in COL1A1 module (Table 3). In ACTA2 module, CNN1 and MMRN1 were regarded as the independent prognostic factors. As for ADCY5 module, FBN1, GLP2R and GUCY1B3 were statistically significant.

**Table 3:** Multivariate Cox regression survival analysis of DEGs from PPI networks.

Variable	OS		
	Exp(B)	95%CI	*p*-value
**Module 1**			
THSD7A	1.226	1.024–1.467	0.026
ADAMTS12	1.242	1.010–1.527	0.040
CDH2	1.138	1.017–1.272	0.024
**Module 2**			
CNN1	1.280	1.022–1.604	0.032
MMRN1	1.205	1.055–1.376	0.006
**Module 3**			
FBN1	1.416	1.131–1.774	0.002
GLP2R	1.146	1.021–1.286	0.020
GUCY1B3	1.681	1.019–2.773	0.042

The bold indicated the result statistically significant.

### ADAMTS12 is upregulated in GC and predicts worse overall survival

**Figure 5:**
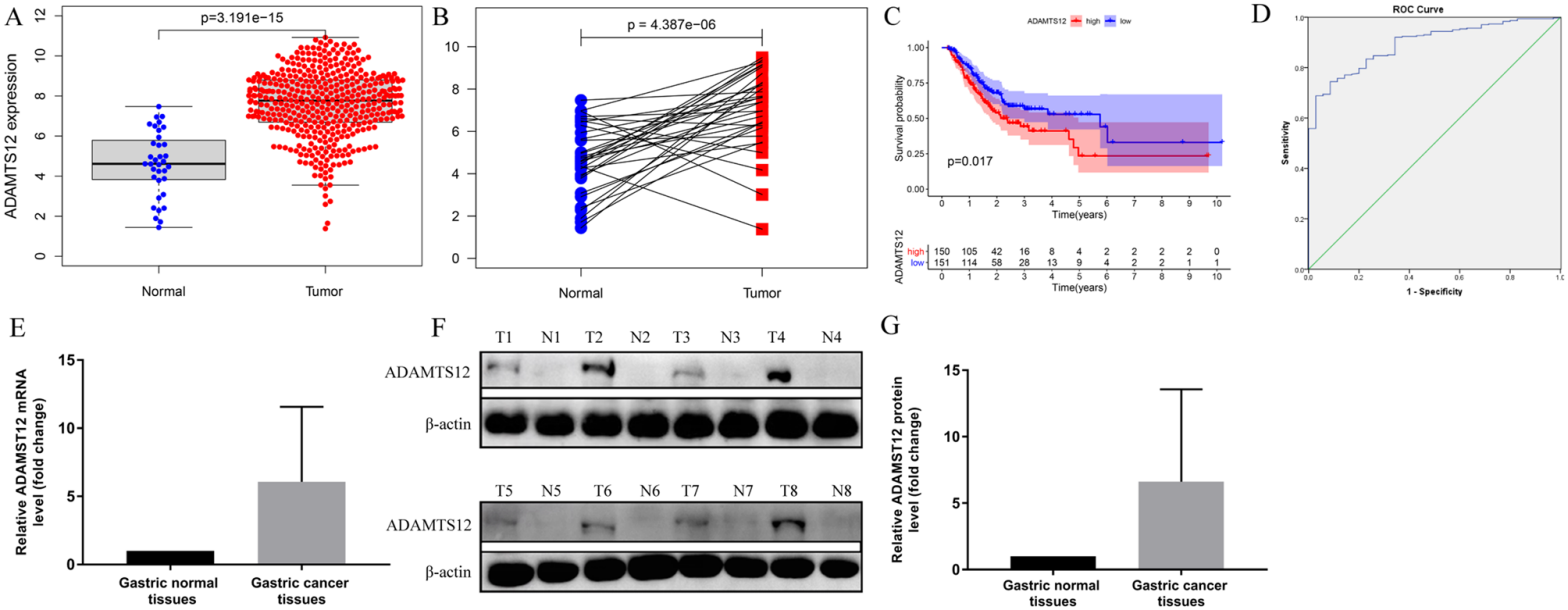
ADAMTS12 is upregulated in GC cases and predicts worse overall survival. (**A**) Differential expression of ADAMTS12 in the normal tissue and GC cases (*p* < 0.001, by Wilcoxon rank sum test). (**B**) Paired analysis of differential expression of ADAMTS12 in normal and tumor samples of the same patients (*p* < 0.001, by Wilcoxon rank sum test). (**C**) Survival analysis of high versus low ADAMTS12 expression groups (*p* = 0.017 by log-rank test). (**D**) ROC curve for the performance of ADAMTS12 in the prediction of GC. (**E**) qRT-PCR analysis of ADAMTS12 mRNA expression in 30 gastric cancer tissues and their pair-matched adjacent normal tissues. GAPDH served as the internal control and data standardization. The data shown the average fold change of ADAMTS12 expression (2^−ΔCT^). (**F**,**G**) Western blot validation of ADAMTS12 protein in GC cases and the matched adjacent normal tissues. β-actin was used as the data normalization.

We selected ADAMTS12 from the top module as the research focus. In this research, we divided all GC cases into the high and low expression groups according to the median expression level of ADAMTS12. Wilcoxon rank sum test illustrated that the ADAMTS12 expression level in tumor group was significantly higher than that in normal group (Fig. 5A). The same result was obtained by paired analysis of tumor and normal specimens of the same cases (Fig. 5B). Survival analysis revealed that the OS of high ADAMTS12 expression cases was significantly lower than that of the low expression ones (Fig. 5C). ROC curve analysis suggested that ADAMTS12 had a certain predictive value for the diagnosis of GC (*p* < 0.001). The area under the curve was 0.894 (95%CI: 0.853–0.936) (Fig. 5D).

To further confirm the expression pattern of ADAMTS12 in clinical situations, qRT-PCR was used to compare the mRNA expression in 30 gastric cancer tissues and their pair-matched adjacent normal tissues. As shown in Fig. 5E, the high expression rate of ADAMTS12 in GC was 93.3% (28/30) (Tumor/Normal > twofold), with up to 23-fold increases in cancer tissues. Simultaneously, the corresponding protein was detected with western blot. Consistent with mRNA data, ADAMTS12 protein levels in cancer tissues were also significantly increased, with a mean value of 5.5 compared with normal tissues as 1.0 (Fig. 5F,G). Then we detected the expression of ADAMTS12 in human gastric cancer cell lines (MKN45), it was highly expressed and could be used for further investigation (Fig. 9B).

### ADAMTS12 could be a potential biomarker of GC TME conversion

**Figure 6:**
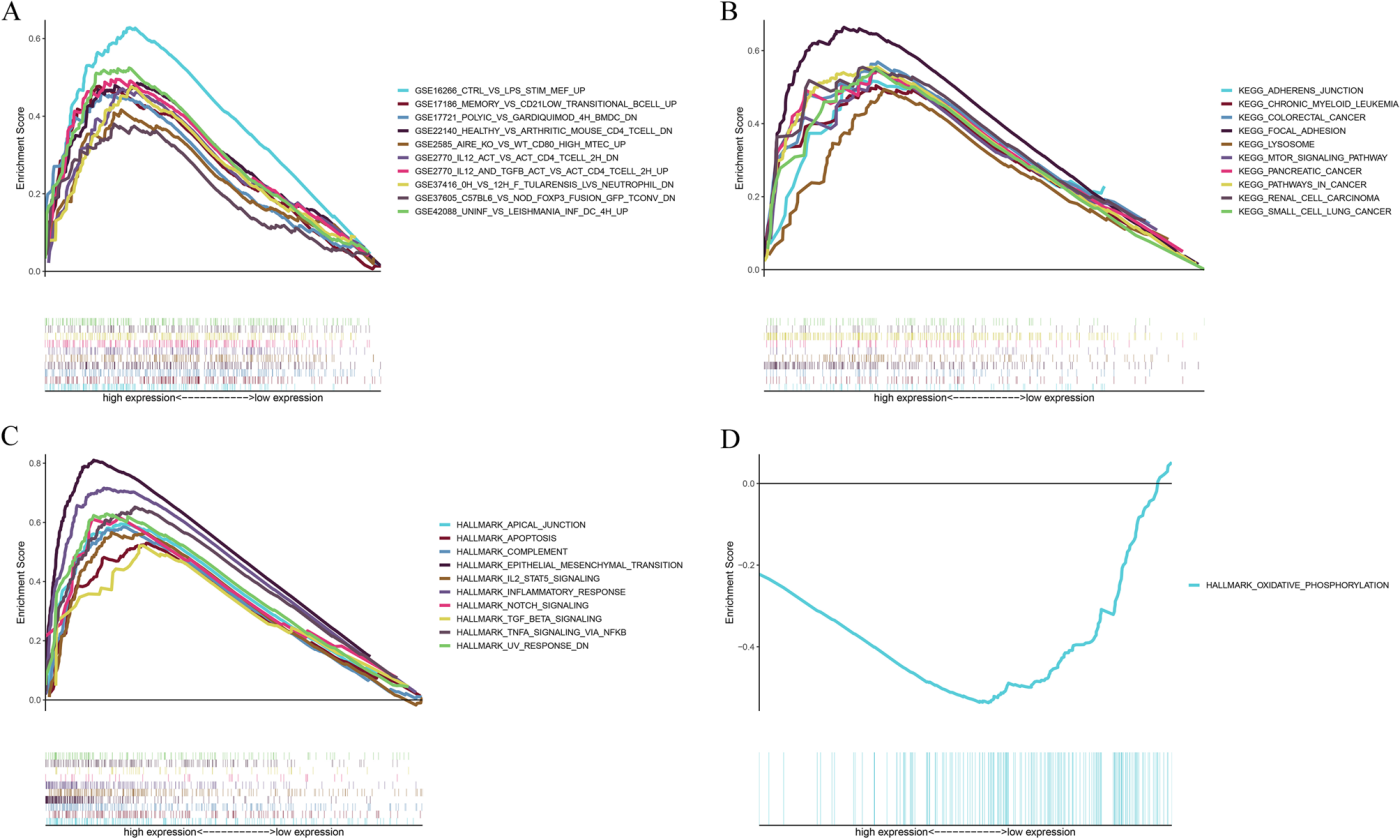
GSEA for cases with high ADAMTS12 expression and low expression. (**A**) C7 collection enriched gene sets in ADAMTS12 overexpression group (Gene sets with NOM *p* < 0.05 and FDR q < 0.05 were supposed to be significant. The top 10 gene sets are shown in the map). (**B**) KEGG collection enriched gene sets in ADAMTS12 overexpression group. (**C**) HALLMARK collection enriched gene sets in ADAMTS12 overexpression group. (**D**) The HALLMARK collection enriched gene set in low ADAMTS12 expression group.

To further explore the potential role of ADAMTS12 in GC TME, GSEA was performed in ADAMTS12 high and low expression groups. For ADAMTS12 high expression group, in C7 collection defined by MSigDB, it was enriched in multiple immunologic and immune functional gene sets (Fig. 6A) including complement and interferon response. As to KEGG enrichment analysis, the gene sets were mainly enriched in cancer-associated signaling pathways. In addition, they were also related to focal adhesion and adherens junction pathways. These signaling events culminated in reorganization of the actin cytoskeleton and would be a prerequisite for changes in cell shape, polarity, motility and proliferation, and gene expression. The above gene sets also closely bound up with TGF-beta, mTOR, NOD-like receptor signaling pathways, etc., which provided a wide spectrum of cellular functions such as proliferation, apoptosis, differentiation and migration (Fig. 6B). In HALLMARK enrichment analysis, the high ADAMTS12 expression group gene sets mainly enriched in multiple immune functional gene sets (Fig. 6C). However, only oxidative phosphorylation gene set was enriched in the low ADAMTS12 expression group (Fig. 6D, NOM *p* = 0.029 FDR = 0.061). Compared with normal cells, tumor cells have a higher level of glycolysis, suggesting that the oxidative phosphorylation level might be reduced. Researchers have confirmed that tumor cells would adapt to the changes of metabolic environment by switching between glycolysis and oxidative phosphorylation^29^ and the use of metformin under fasting conditions would significantly inhibit tumor growth^29^. Therefore, ADAMTS12 may be linked to tumor energy metabolism. The above results indicated that ADAMTS12 could be a potential biomarker of TME conversion.

### The relevance between ADAMTS12 and TICs proportion in GC

**Figure 7:**
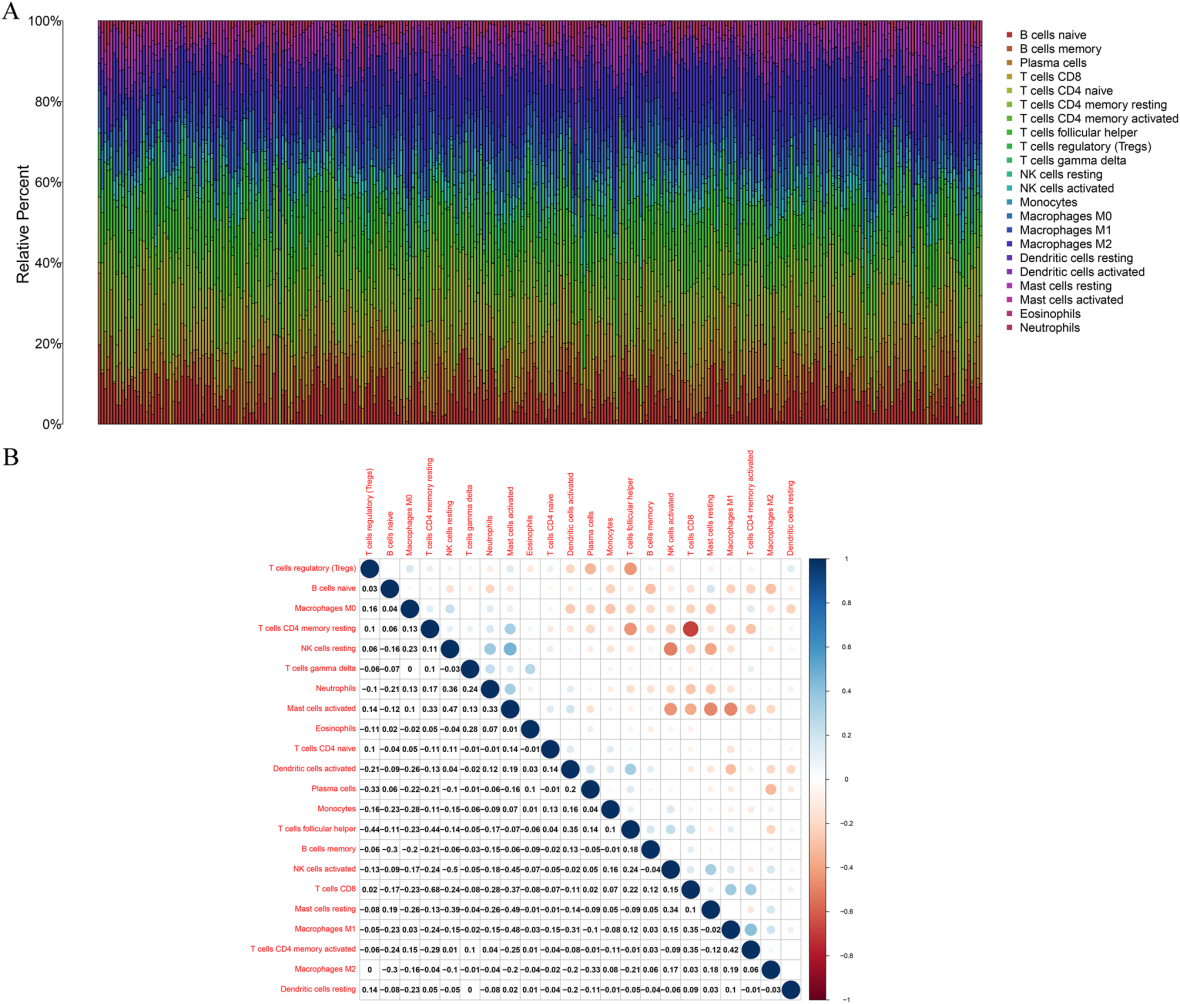
The proportion and correlation analysis of TIC in GC cases. (**A**) The proportion of 22 TICs in GC cases. (**B**) The interrelationship among 22 kinds of TICs (Pearson coefficient test). Each number represents the correlation *p* value between every two types of immune cells. The color depth indicates the corresponding correlation value between two types of cells.

**Figure 8:**
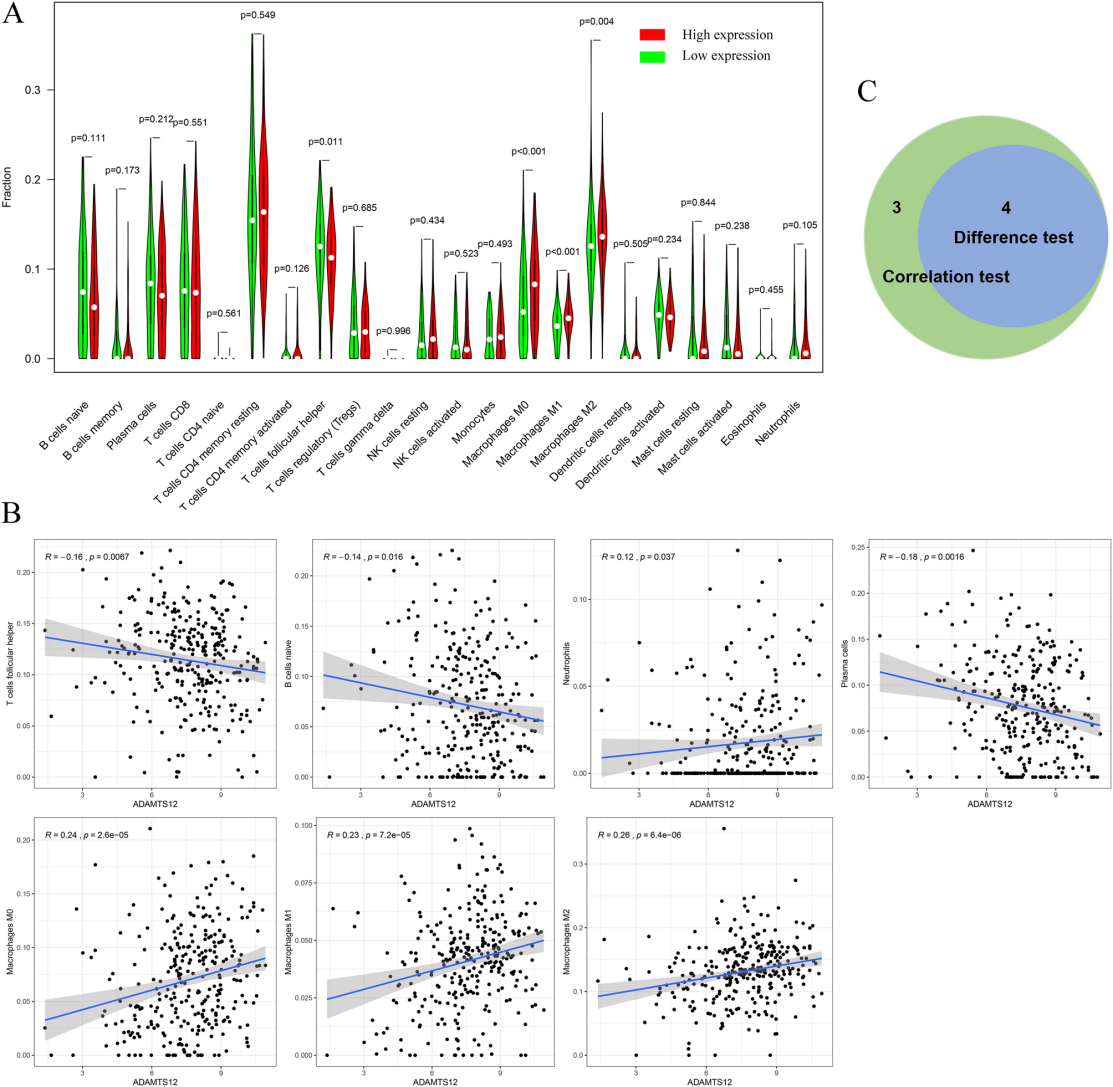
Relationship between TICs proportion and ADAMTS12 expression. (**A**) The proportion difference of 22 kinds of immune cells in high vs. low ADAMTS12 expression groups (Wilcoxon rank sum test). (**B**) TICs related to ADAMTS12 expression (*p* < 0.05, Pearson coefficient test). (**C**) TICs correlated with ADAMTS12 expression codetermined by difference and correlation tests.

In order to illustrate the relevance between ADAMTS12 expression and immune microenvironment, CIBERSORT computational method was implemented to analyze the proportion of immune subpopulations in tumor infiltration, and the map of 22 immune cells in GC samples was constructed (Fig. 7). The correlation and difference analysis results showed that there were 7 kinds of TICs relevant to ADAMTS12 expression (Fig. 8). Among them, 4 kinds were positively correlated, including Macrophages M0, Macrophages M1, Macrophages M2 and Neutrophils; 3 kinds were negatively correlated, including B cells naïve, Plasma cells and T cells follicular helper. These results further demonstrated that ADAMTS12 level influenced the immune activity of GC TME.

### ADAMTS12 knockdown inhibits the proliferation, invasion and migration of GC

**Figure 9:**
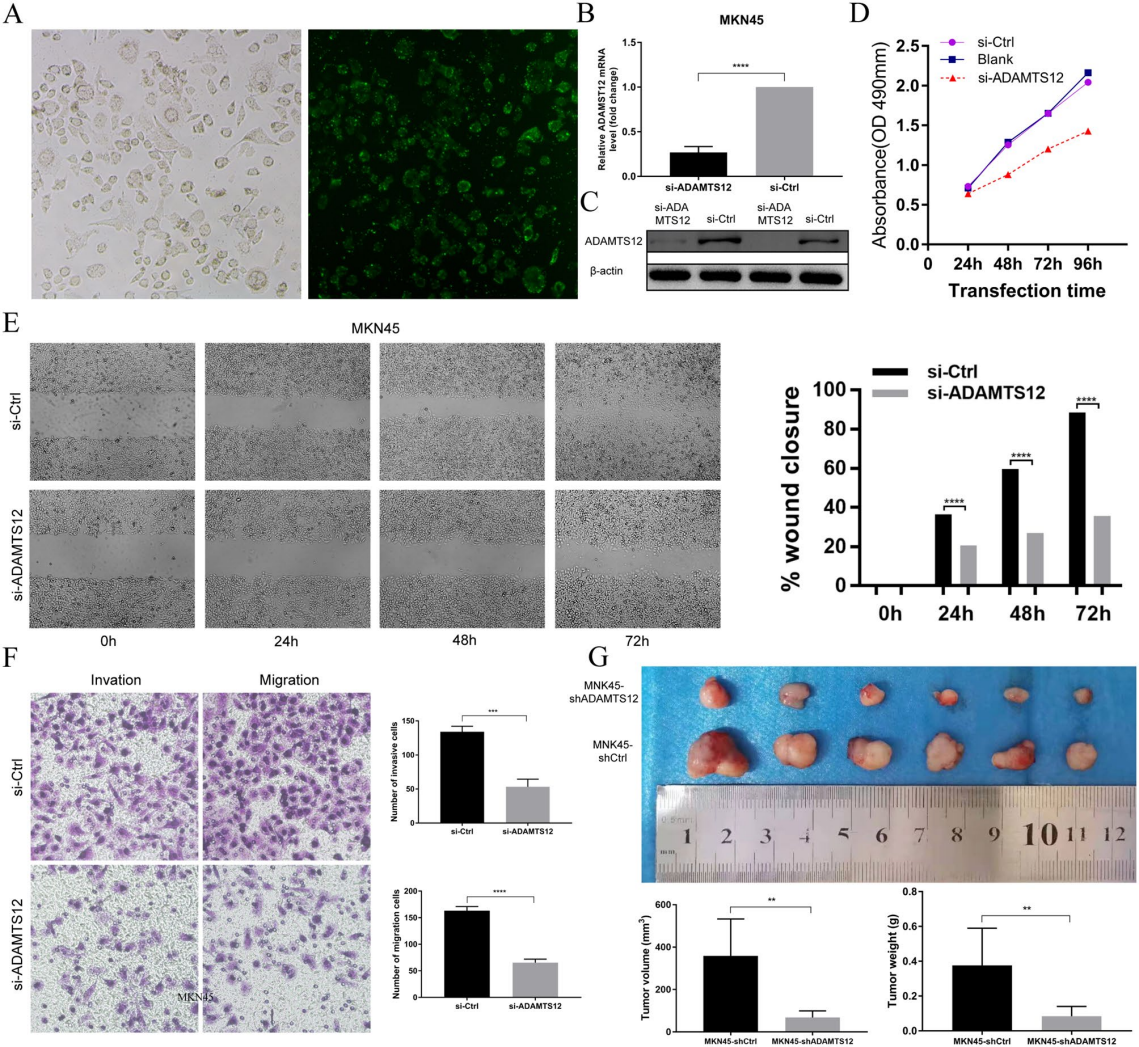
ADAMTS12 knockdown inhibits the proliferation, invasion and migration of GC cells in vitro and vivo. (**A**) The transfection efficiency of siRNA in MKN45 cells was detected by microscopy at 24 h after transfection of a FAM-labled siRNA negative control. (**B**,**C**) MKN45 cells were transfected with ADAMTS12 siRNA or siRNA negative control (50 nM), and then the relative expression of ADAMTS12 mRNA was determined by qPCR at 48 h after transfection. The relative expression of ADAMTS12 protein was detected at 72 h after transfection. Data were presented as mean ± SD. (**D**) cell proliferation was determined by CCK-8 assay and detected at 24, 48, 72, and 96 h after transfection. (**E**) Wound-healing assay indicates that silencing of ADAMTS12 inhibited MKN45 cell migration. (**F**) Transwell experiment demonstrates that knockdown of ADAMTS12 significantly reduced the migratory and invasive abilities of GC cells. (**G**) The MKN45-shADAMTS12, MKN45-shCtrl cells were subcutaneously inoculated into the left-side axilla of BALB/c nude mice (n = 6/each group). Data points are presented as the mean ± SD tumor volume and weight.

To investigate the effect of ADAMTS12 on cell proliferation in vitro, we transfected specific ADAMTS12 siRNA into a gastric cancer cell line (MKN45). The transfection efficiency of siRNA was detected by microscopy at 24 h after transfection of a FAM-labelled siRNA negative control (Fig. 9A). The expression of ADAMTS12 mRNA was identified 48 h after transfection (Fig. 9B), and the protein expression of ADAMTS12 was determined at 72 h after transformation (Fig. 9C). CCK8 experiment indicated that compared with the negative control, inhibiting ADAMTS12 significantly reduced the growth rate of MKN45 (Fig. 9D). Transwell experiment and wound migration were used to detect the effect of ADAMTS12 on the migration activity of MKN45 cells. In cells with low ADAMTS12 expression, the migration ability was significantly reduced (Fig. 9E,F). These data indicated that ADAMTS12 gene knockdown inhibited the proliferation, invasion and migration of GC.

To further explore whether ADAMTS12 can also promote GC cell proliferation in vivo. The MKN45-shADAMTS12, MKN45-shCtrl cells were subcutaneously inoculated into BALB/c nude mice. As shown in Fig. 9G the average volume and weight of the tumors with MKN45-shADAMTS12 were markedly lower than those with MKN45-shCtrl (*p* < 0.001). These results suggested that ADAMTS12 exerted a growth-promoting function in human gastric cancer.

## Discussion

Gastric cancer is a common upper gastrointestinal malignancy and often diagnosed in advanced stages. Previous studies on the mechanism of GC have mainly focused on tumor cells themselves, including oncogenes and signaling pathways^30^. However, accumulating evidence has shown that GC invasion and metastasis were the result of co-evolution of cancer cells and the microenvironment^31,32^.

In solid tumors, the cancer cells are wrapped in an intricate mixture of nontumorous cells and matrix components, which denominated as the tumor microenvironment (TME). Under selection pressure, TME can provide high levels of chemokines, such as C-X-C chemokine ligand (CXCL) 12 and stromal derived factor (SDF)1^33^ and reduce the presence of CTLs expressing PD-1 to achieve the immune escape of tumor cells^34^. In fact, TME would also reduce the efficacy of cytotoxic drugs. By enhancing the activity of NF-kB and production of cytokines (including anti-apoptotic cytokines IL-6, IL-1a, and GM-CSF), TME would protect tumor cells from DNA damage caused by antitumor drugs such as doxorubicin^35^. During radiotherapy, fibroblast would be in a susceptible state. Once activated, the cell would produce plenty of collagen and form dense fibrous tissue leading to radiation fibrosis^36^, while bone marrow-derived cells (BMDC) would induce tumor angiogenesis^37^, both of which ultimately promote tumor growth. But from the other perspective, we can also improve the treatment efficiency through TME. For instance, blocking integrin signaling and its downstream tyrosine kinase signaling would increase the efficacy of radiotherapy and molecular targeting treatment^38^. In addition, Bussard et al.^39^ demonstrated that by grafted into the developing murine mammary gland the aggressive human breast cancer cells would transform into “normal”. It is possible that the suppression of malignancy by developmental microenvironments described above is the result of a reduced fitness differential between the transformed and normal cells. Studies have pointed out that overcoming immune surveillance was a key part of tumorigenesis and immune cells from both the primordial and adaptive immune systems have been found in TME^13,40^. Therefore, reactivating the inhibited T cells may be another effective potent anticancer therapy^41^. In summary, lucubrate the TME may improve the efficacy of current therapies and provide new opportunities for therapeutic targeting.

**Figure 10:**
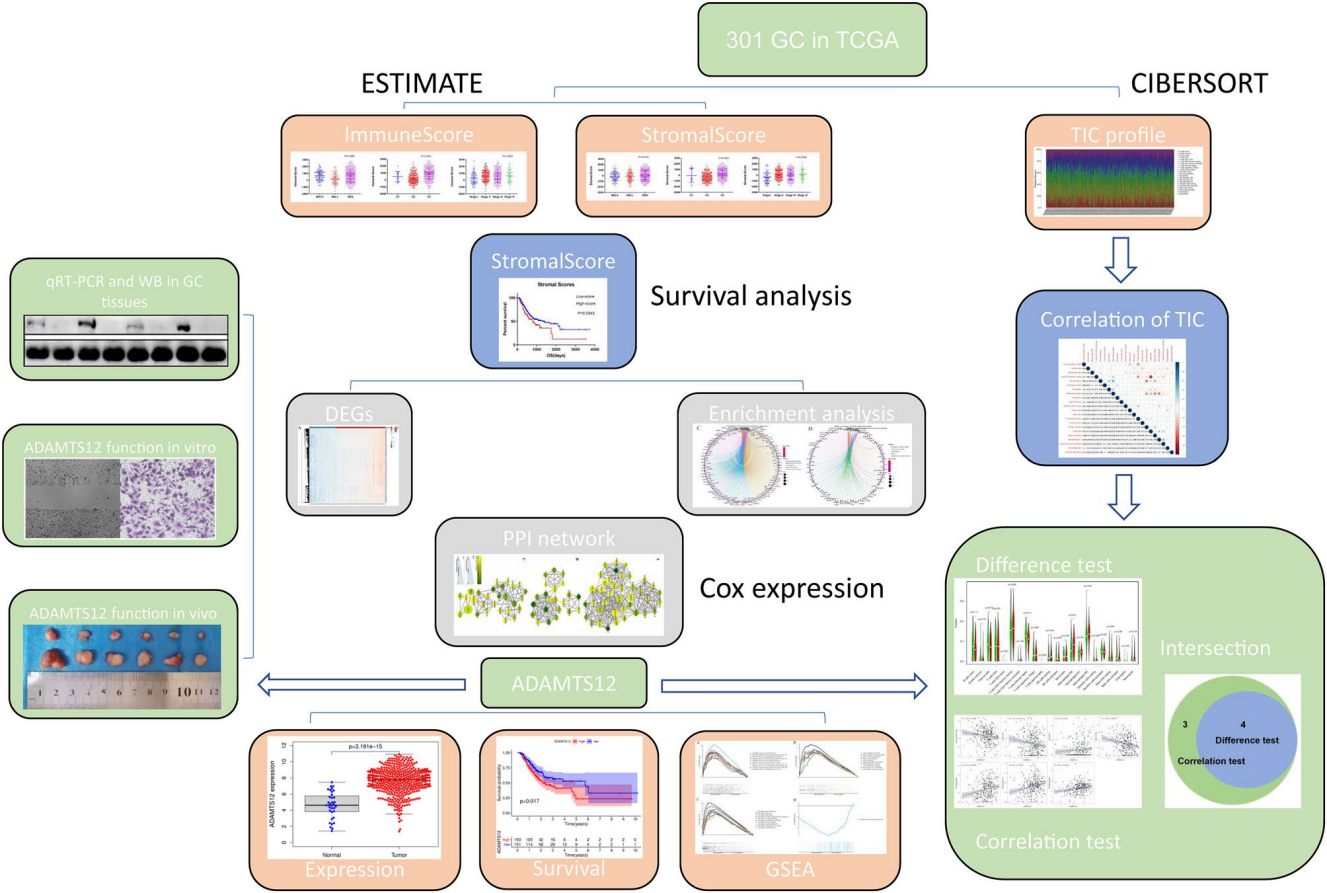
The outline diagram of the current work.

In the manuscript, we attempted to identify tumor microenvironment-related genes which also associated with the overall survival of GC patients (Fig. 10). First, we confirmed that the scores provided by ESTIMATE algorithm mode could be correlated with histological grade, clinical stage and subtype classification of GC cases. Statistics analysis indicated that it was also significant in tumor-related gene mutations and higher stromal scores signified worse prognosis. On account of this theoretical basis, we conducted the further statistical analysis from comparison of high versus low stromal score groups, and identified 834 differentially expressed genes, most of which were involved in tumor microenvironment. GO analysis indicated that most significant features are mapped to TME-related GO terms, including extracellular matrix organization, immunoglobulin binding and cytokine binding. By KEGG enrichment analysis, up-regulated DEGs were enriched in Cell adhesion molecules (CAMs), PI3K−Akt signaling pathway, ECM−receptor interaction, B cell receptor signaling pathway, focal adhesion et al. Fig. 3D. Since ECM is a component of TME, the ECM–receptor interaction signaling pathway participates in the regulation of tumor microenvironment^42^. Cell adhesion molecules are involved in immune surveillance and homeostasis. TME disorder often leads to alterations of CAMs and ultimately results in changes of cell adhesion and migration, causing inflammatory diseases and even tumors^43^. PI3K−Akt signaling pathway participates in the regulation of immune function: it has been shown that the downstream molecule mTOR could interact with T cells, B cells, NK cells, neutrophils, DC cells and other immune cells, and participate in the immune regulation of tumor development^44^. Rapamycin, an mTOR inhibitor, would inhibit the proliferation of T cells and NK cells, and the maturation and differentiation of DC cells and macrophages, thus impairing the anti-tumor immunoregulation^44^. Therefore, the overall function of DEGs seems to be mapped on immune-related activities. Subsequently, an overall survival analysis of all 834 DEGs was performed, 501 of which were associated with poor prognosis. By cross-validation with the GSE26253 cohort of 432 GC cases, we identified 81 tumor microenvironment-related genes whose expression levels were significantly associated with prognosis, indicating that our analysis has predictive value. In addition, we had established 10 protein–protein interaction modules, and selected the top three for further analysis (Fig. 4). The selected modules all have a certain correlation with TME. Among the modules, interrelated nodes including FSTL1, THBS2, COL1A1, COL10A1, HGF, EFEMP1 et al. have already been proved to promote proliferation, angiogenesis, migration and invasiveness in GC cell lines or patient samples, indicating a poor prognosis.

ADAMTS12 belongs to the ADAMTS (a disintegrin and metalloproteinase with thrombospondin motifs) protein family. It contains 8 TS-1 motifs, which may regulate cell adhesion and tumor processes by proteolytic activity. The biological function of ADAMTS12 in malignant tumors is still ambiguous. One research had pointed out that ADAMTS12 was abnormally overexpressed in esophageal squamous cell carcinoma and negatively regulated by the expression of migration inhibitor lncRNA HCG22^45^. Li et al.^46^ revealed that ADAMTS12 acted as a cancer promoter in colorectal cancer by activating the Wnt/β-catenin signaling pathway in vitro. As for lung cancer, ADAMTS12 promoted the migration and tumorigenesis^47^. In contrast, it showed an antitumor effect in renal cell carcinoma and breast cancer^48,49^. Here, we embarked from the transcriptome analysis of gastric cancer based on TCGA database and clinical patients and confirmed that ADAMTS12 was significantly overexpressed in GC tissues, the up-regulated expression was associated with poor overall survival. ADAMTS12 knockdown inhibits the proliferation, invasion and migration of GC in both vivo and vitro.

To further explore the correlation between ADAMTS12 expression and TME, GSEA was conducted, the results revealed that in addition to immune functional gene sets including complement and interferon response, cancer-associated signaling pathways and adherens junction pathways were also significantly enriched in the ADAMTS12 high-expression group. Meanwhile, only oxidative phosphorylation gene set was enriched in the ADAMTS12 low-expression group. The above results suggested that ADAMTS12 might participate in the regulation of immune cell function and the status conversion of metabolism. Subsequence analysis towards TIC sustained this viewpoint. In the manuscript, according to the CIBERSORT analysis of TICs proportion, we considered that along with the upregulation of ADAMTS12, the contents of Macrophages M0, Macrophages M1 and Macrophages M2 also increased. In TME, tumor associated macrophage (TAM) would express a variety of cytokines, which stimulate tumor cell proliferation and angiogenesis, including EGF epithelial growth factor, PDGF platelet growth factor, etc. Meanwhile, TAM would degrade the extracellular matrix and basement membrane by up-regulating proteolytic enzymes to promote tumor invasion. In addition, the down-regulation of T cells follicular helper indicated a decline in immune function against GC. Accordingly, supported by the above conclusions, we considered that ADAMTS12 might be a GC promoter and had the potential to be a prognostic biomarker and therapeutic target.

The interaction between GC and the tumor microenvironment greatly affects the evolution of the malignancy, which in turn impacts subtype classification, recurrence, drug resistance, and overall survival. In this work, we focused on the relationship between genes and the microenvironment, as well as the impact on prognosis. Our results provided more data for the decoding of complex interactions between tumors and the tumor environment. In conclusion, a list of genes related to tumor microenvironment could be extracted by functional enrichment analysis of TCGA database. Some previously overlooked genes have the potential to become new tumor markers or therapeutic targets. ADAMTS12 is a potential prognostic factor in GC patient, and might be used as an indicator of TME state transition. However, there are still some limitations in this study. More clinical samples should be included to further analyze the correlation between clinicopathological factors and ADAMTS12 expression. In addition, the regulatory mechanism and protein interaction of ADAMTS12 deserve deeper exploration. Hence, through further research on ADAMTS12, a new understanding of the potential relationship between tumor microenvironment and GC prognosis could be achieved.

## Materials and methods

### Database

Gene expression profile for GC was obtained from UCSC Genome Bioinformatics portal, which was measured experimentally using the Illumina HiSeq 2000 RNA Sequencing platform designed by the University of North Carolina TCGA genome characterization center. Clinical data such as gender, age, tumorous grading and overall survival was also downloaded from the portal. Immune scores and stromal scores were calculated by applying the ESTIMATE algorithm to the downloaded database^9^. For validation, a gene expression dataset GSE26253^26^ was obtained from GEO (Illumina GPL6947 platform, Illumina HumanHT-12 V3.0 expression beadchip).

### Evaluation of gene expression profiling in silicon

The package limma was used to perform data analysis^50^. Fold change > 2 and adj. *p* < 0.05 were set as the cut-offs to filtrate DEGs. The heatmaps were built by the open source web tool ClustVis^51^. The protein–protein interaction (PPI) network was constructed by Search Tool for the Retrieval of Interacting Genes (STRING; http://string-db.org) (version 10.0)^52^ and retrieved from Cytoscape software (v3.6.1)^27^. The plug-in Molecular Complex Detection (MCODE) (version 1.4.2) which acted as a clustering tool was used to identify densely connected network regions^28^. Only individual networks with score greater than 1000 were included for further analysis. The connectivity degree of each node was calculated.

GO and KEGG enrichment analyses were performed with the packages of cluster Profiler, enrichplot and ggplot2. The items with *p* < 0.05 were considered significantly enriched. Molecular Signatures Database provided Hallmark, KEGG, GO and C7 gene sets of v6.2 collections as the target sets. The software gsea-3.0 was used to perform the further analysis. Listed gene sets with NOM *p* < 0.05 were screened as significant. To estimate the distribution of TIC abundance, the CIBERSORT calculation method was used. Only cases with *p* < 0.05 were filtered for the further analysis.

### Tissue collection

Fresh gastric cancer specimens and paired non-cancerous tissues from 30 patients were selected from the Department of general surgery, Second Affiliated Hospital of Harbin Medical University (Harbin, China) between January 2014 and January 2015. All tissue specimens were immediately frozen in liquid nitrogen and stored at − 80 ℃ until use. The study protocol was approved by the Ethics Committee of the Second Affiliated Hospital of Harbin Medical University and samples were obtained with informed consent. We confirm that all methods were performed in accordance with the relevant guidelines and regulations.

### Cell culture and transfection

Human gastric cancer cell line, MKN45 was obtained from the American Type Culture Collection (ATCC). The cell line was cultured in RPMI 1640 and supplemented with 10% fetal bovine serum (FBS) (Gibco) at 37 °C and 5% CO_2_. SiRNA against ADAMTS12 and scrambled siRNA negative control were synthesized by GenePharma. Transient transfection was performed with liposome 2000 according to the manufacturer's instructions. The transfected cells were incubated and harvested for subsequent detection.

### Cell proliferation, migration and invasion assay

Cell count KIT-8 assay (C0038, Beyotime) was utilized to detect the proliferation capacity of normal and ADAMS12-deficient GC cells. 2 × 10^3^ cells were plated in 96-well plates with the 450 nm absorption being recorded every 24 h.

For Transwell migration and Matrigel invasion, after 24 h incubation, the migrated cells were fixed with methanol and stained with crystal violet. The migrated and invaded cells were captured and calculated using microscope.

### Establishment of animal models

All animal experiments were performed based on the institutional protocols, concerning the use and care of laboratory animal. We confirm that the experimental protocol was approved by Animal Care and Use Committee of Harbin Medical University and followed the recommendations in the ARRIVE guidelines. In vivo tumor growth assay, 4-week-old male BALB/c nude mice were injected with equal numbers of cells stably infected with pL/shRNA/F-ADAMTS12 or pL/shRNA/F (5 × 10^6^ cells/injection for MKN45). Tumor diameters were measured weekly with the precision caliper. Calculating the tumor mass volume (xenograft).

### Quantitative real-time RT-PCR

Total RNA was extracted by TRIzol reagent (15596026, Invitrogen). ABScript II one step SYBR Green RT-qPCR kit (RK20404, ABclonal) was used to conduct qRT-PCR. Primer sequences are as follows: qRT-ADAMTS12 (F: 5’-GCCATGGACTGACTGGATTT-3’, R:5’-TGCCTCCTGTAAACGATGTG-3’). GAPDH was considered as an appropriate internal control, and fold changes were calculated through relative quantification (2^−ΔCT^).

### Western blot analysis

The soluble protein was prepared in RIPA buffer. Equivalent protein (20–50 μg) was subjected to 6% SDS-PAGE, and transferred to electrophoretically PVDF membranes. After being placed in blocking buffer for 1 h, the membranes were blotted with specific primary rabbit antibodies to ADAMTS12 (1:1000; Proteintech) and β-actin (1:2000; ABclonal) for 24 h at 4℃, followed by secondary antibodies for 1 h. Then horseradish peroxidase (HRP) was added and antigen–antibody complex was detected by enhanced chemiluminescence (ECL) reagent. β-actin was used as a loading control.

### Statistical analysis and survival curve

A χ^2^ test was used to analyze the association between immune/stromal score and clinicopathological variables. In the univariate survival analysis, OS was evaluated according to the log-rank test and univariate Cox regression model to assess the differences between the levels of potential prognostic factors. In the multivariate analysis, a Cox proportional hazard regression model was applied to predict if immune/stromal score was an independent predictive factor of OS. The relationship between DEGs’ expression level and patients’ OS/RFS was illustrated by Cox proportional hazards regression model and Kaplan–Meier plots. Results were tested by log-rank test. *p* < 0.05 was considered statistically significant. The Receiver Operating Characteristic (ROC) was used to evaluate the diagnostic value of ADAMTS12 fo rGC. *p* < 0.05 was statistically significant. All statistical analyses were performed using SPSS version 20.0 software (IBM Corp., Armonk, NY, USA).

## Supplementary Information

Supplementary Information.
