# Supplementary material for: ADAMTS12 acts as a tumor microenvironment related cancer promoter in gastric cancer
Source: Sci Rep. 2021 May 26;11:10996. doi: 10.1038/s41598-021-90330-3 (PMC8154915; doi:10.1038/s41598-021-90330-3)

ADAMTS12 acts as a tumor microenvironment related cancer promoter in gastric cancer

Yangming Hou[1], Yingjuan Xu[2], Dequan Wu[1*]

[1]Department of Hepatic Surgery, The Second Affiliated Hospital of Harbin Medical University, No. 246 Xuefu Avenue, Harbin 150086, Heilongjiang, China.

[2]Department of obstetrics and gynecology, China-Japan Union Hospital, Jilin University, No. 126 Xiantai Avenue Changchun 130033, China.

Correspondence to: Dequan Wu; email: dqwu58@163.com

Supplementary Table 1.

Upregulated DEGs extracted from comparison of high vs. low stromal score groups.

Gene.names	logFC	Ave Expr	t	P.Value	adj.P.Val	B	regulated
CDH11	2.107086	10.28104	19.55531	1.68E-55	3.45E-51	115.5789	Up-Regulated
ZEB2	1.783956	9.415214	18.87549	5.99E-53	6.15E-49	109.7588	Up-Regulated
FBN1	2.168891	11.19996	18.4881	1.72E-51	8.83E-48	106.4328	Up-Regulated
PLXDC2	2.036302	8.570471	18.26022	1.24E-50	4.25E-47	104.4738	Up-Regulated
MAFB	1.801569	9.47666	17.95324	1.79E-49	5.24E-46	101.8325	Up-Regulated
C1S	1.97571	11.97922	17.9342	2.11E-49	5.41E-46	101.6686	Up-Regulated
COLEC12	2.791793	6.939806	17.68319	1.87E-48	3.83E-45	99.50723	Up-Regulated
C1R	1.941784	11.81894	17.62599	3.07E-48	5.73E-45	99.01459	Up-Regulated
OLFML1	1.710356	7.576869	17.49029	9.98E-48	1.71E-44	97.84558	Up-Regulated
RFTN1	1.594129	9.29306	17.45636	1.34E-47	2.12E-44	97.55324	Up-Regulated
RAB31	1.660987	10.38151	17.34329	3.58E-47	5.26E-44	96.57907	Up-Regulated
CCDC80	2.999266	10.27288	17.29712	5.35E-47	6.87E-44	96.18126	Up-Regulated
GGT5	1.915346	9.38305	17.28476	5.96E-47	7.20E-44	96.07474	Up-Regulated
COL8A2	2.294054	7.726121	17.19876	1.26E-46	1.44E-43	95.33375	Up-Regulated
COL8A1	2.864677	8.802955	17.08733	3.32E-46	3.59E-43	94.37366	Up-Regulated
AEBP1	2.148953	12.22637	17.04567	4.77E-46	4.89E-43	94.01468	Up-Regulated
LUM	1.982594	12.86447	17.00426	6.83E-46	6.57E-43	93.65785	Up-Regulated
C10orf72	2.077523	7.807479	17.00086	7.04E-46	6.57E-43	93.62855	Up-Regulated
ANTXR1	1.959742	11.35811	16.97245	9.01E-46	8.04E-43	93.3838	Up-Regulated
GLT8D2	1.894713	7.364171	16.96424	9.68E-46	8.28E-43	93.31305	Up-Regulated
DDR2	2.619145	7.903018	16.93758	1.22E-45	1.00E-42	93.08333	Up-Regulated
DCN	2.124677	12.28994	16.92149	1.40E-45	1.11E-42	92.9447	Up-Regulated
TIMP3	2.062227	12.07816	16.91227	1.52E-45	1.16E-42	92.86532	Up-Regulated
GUCY1A3	2.279343	9.836663	16.90716	1.59E-45	1.17E-42	92.82131	Up-Regulated
PCDHGA12	2.345298	5.950627	16.83904	2.87E-45	2.03E-42	92.23445	Up-Regulated
SERPINF1	2.144712	10.28715	16.74927	6.27E-45	4.29E-42	91.46109	Up-Regulated
TMEM119	2.145764	8.617048	16.63045	1.76E-44	1.17E-41	90.43771	Up-Regulated
GAS1	3.017572	7.257189	16.5794	2.74E-44	1.76E-41	89.9981	Up-Regulated
LRRC32	1.750644	10.01093	16.54723	3.63E-44	2.26E-41	89.72115	Up-Regulated
BNC2	2.5192	7.505235	16.52332	4.47E-44	2.70E-41	89.51523	Up-Regulated
PDGFRB	1.627913	11.72451	16.51101	4.97E-44	2.92E-41	89.40926	Up-Regulated
SLIT2	3.693431	6.055103	16.48476	6.24E-44	3.48E-41	89.18326	Up-Regulated
EMILIN1	1.980695	10.91847	16.47945	6.54E-44	3.53E-41	89.13757	Up-Regulated
VCAM1	1.747379	9.013912	16.45617	8.00E-44	4.21E-41	88.93713	Up-Regulated
CTSK	1.68803	10.17171	16.43167	9.90E-44	5.08E-41	88.72629	Up-Regulated
GNB4	1.665223	9.590568	16.38432	1.49E-43	7.48E-41	88.31869	Up-Regulated
ISLR	2.569768	10.18527	16.29611	3.21E-43	1.57E-40	87.55959	Up-Regulated
TSHZ3	1.768128	7.70955	16.20672	6.98E-43	3.33E-40	86.79057	Up-Regulated
PRRX1	2.352442	8.686396	16.20088	7.34E-43	3.43E-40	86.74037	Up-Regulated
SERPING1	1.918755	12.24874	16.18271	8.60E-43	3.92E-40	86.5841	Up-Regulated

THY1	1.679942	11.30632	16.17414	9.26E-43	4.13E-40	86.51034	Up-Regulated
PABPC5	1.915236	3.675702	16.11562	1.54E-42	6.72E-40	86.00709	Up-Regulated
ST6GALNAC 5	2.369906	6.343502	16.05082	2.70E-42	1.11E-39	85.44998	Up-Regulated
FSTL1	1.596327	12.27639	16.0505	2.71E-42	1.11E-39	85.44722	Up-Regulated
COL6A2	1.770949	13.25635	16.03363	3.13E-42	1.26E-39	85.30219	Up-Regulated
ASPN	2.504478	9.533315	16.01381	3.72E-42	1.47E-39	85.13185	Up-Regulated
MRAS	1.681282	8.22467	15.9598	5.95E-42	2.26E-39	84.66771	Up-Regulated
GAS7	1.950354	9.315962	15.95568	6.16E-42	2.30E-39	84.63228	Up-Regulated
GLI3	2.158467	7.411563	15.91767	8.57E-42	3.14E-39	84.30574	Up-Regulated
ANGPTL2	1.841391	10.4199	15.8911	1.08E-41	3.88E-39	84.07743	Up-Regulated
TMEM90B	2.365128	5.937275	15.84862	1.56E-41	5.42E-39	83.71259	Up-Regulated
GYPC	1.866308	8.360726	15.84023	1.68E-41	5.73E-39	83.64053	Up-Regulated
FBXL7	1.721679	7.791189	15.80371	2.30E-41	7.74E-39	83.32694	Up-Regulated
GPNMB	2.07851	11.05433	15.79396	2.50E-41	8.29E-39	83.24329	Up-Regulated
FBLN2	2.606884	9.626145	15.77288	3.00E-41	9.79E-39	83.06228	Up-Regulated
NDN	1.980248	7.861311	15.70741	5.30E-41	1.70E-38	82.50034	Up-Regulated
MFAP5	3.151661	7.653165	15.69403	5.95E-41	1.83E-38	82.38551	Up-Regulated
FLRT2	2.561836	7.447335	15.6939	5.95E-41	1.83E-38	82.38441	Up-Regulated
SFRP4	4.104755	9.399994	15.69337	5.98E-41	1.83E-38	82.37989	Up-Regulated
NTNG2	1.852768	5.144473	15.67693	6.89E-41	2.08E-38	82.23883	Up-Regulated
COL6A3	1.800908	13.6003	15.65232	8.53E-41	2.54E-38	82.02761	Up-Regulated
GPR124	1.608504	10.46731	15.64473	9.11E-41	2.67E-38	81.96252	Up-Regulated
TIMP2	1.59676	12.57038	15.63806	9.65E-41	2.75E-38	81.90532	Up-Regulated
FNDC1	3.279762	9.391557	15.62698	1.06E-40	2.99E-38	81.81032	Up-Regulated
ASAM	2.598349	7.179256	15.61162	1.21E-40	3.37E-38	81.67851	Up-Regulated
GUCY1B3	1.738903	8.707209	15.59444	1.41E-40	3.85E-38	81.53118	Up-Regulated
TM6SF1	1.623301	5.950325	15.5742	1.68E-40	4.53E-38	81.35767	Up-Regulated
BHLHE22	2.200592	4.585693	15.53116	2.43E-40	6.49E-38	80.98861	Up-Regulated
OLFML2B	2.029617	9.436246	15.52328	2.61E-40	6.83E-38	80.92111	Up-Regulated
CRISPLD2	1.664427	10.53392	15.52222	2.63E-40	6.83E-38	80.912	Up-Regulated
A2M	1.693285	13.58674	15.50506	3.05E-40	7.83E-38	80.76493	Up-Regulated
FIBIN	1.954615	7.072835	15.46784	4.21E-40	1.07E-37	80.44595	Up-Regulated
VCAN	1.881624	11.74018	15.46221	4.42E-40	1.11E-37	80.39774	Up-Regulated
AKT3	1.790742	8.640164	15.41979	6.38E-40	1.58E-37	80.03431	Up-Regulated
SULF1	2.345712	11.68259	15.37689	9.24E-40	2.26E-37	79.66692	Up-Regulated
COL3A1	2.00803	14.97289	15.36314	1.04E-39	2.51E-37	79.54921	Up-Regulated
DOK5	2.094712	4.81329	15.33678	1.31E-39	3.12E-37	79.32352	Up-Regulated
ITGBL1	3.329029	7.645013	15.33539	1.32E-39	3.12E-37	79.31158	Up-Regulated
FAP	2.35656	8.011842	15.28126	2.11E-39	4.92E-37	78.84831	Up-Regulated
SSC5D	2.612673	8.332775	15.24133	2.98E-39	6.79E-37	78.50671	Up-Regulated
PDLIM3	2.529703	9.785699	15.23423	3.17E-39	7.07E-37	78.44592	Up-Regulated
OMD	3.361607	5.586395	15.21734	3.66E-39	8.09E-37	78.30153	Up-Regulated

BGN	1.884747	13.03105	15.1853	4.83E-39	1.05E-36	78.02755	Up-Regulated
KCNJ8	1.6565	7.331276	15.18012	5.05E-39	1.09E-36	77.9832	Up-Regulated
HTR2A	2.50805	3.671082	15.1226	8.30E-39	1.75E-36	77.4915	Up-Regulated
ZNF521	1.737576	7.34638	15.122	8.34E-39	1.75E-36	77.48645	Up-Regulated
HIC1	1.678825	8.639984	15.06391	1.38E-38	2.85E-36	76.99014	Up-Regulated
KCNE4	1.970298	7.107172	15.05318	1.51E-38	3.10E-36	76.8985	Up-Regulated
ECM2	1.656119	7.545139	15.02188	1.98E-38	4.02E-36	76.6312	Up-Regulated
EVC	2.104733	8.284957	15.00304	2.32E-38	4.63E-36	76.4703	Up-Regulated
RUNX1T1	2.432685	5.843104	14.98651	2.68E-38	5.29E-36	76.32919	Up-Regulated
DPYSL3	2.376646	11.07743	14.98249	2.77E-38	5.43E-36	76.29492	Up-Regulated
SGCD	2.212972	8.140718	14.97513	2.96E-38	5.73E-36	76.2321	Up-Regulated
PDE1A	2.082169	6.287074	14.92598	4.51E-38	8.63E-36	75.8127	Up-Regulated
CMKLR1	1.769718	7.9413	14.92527	4.54E-38	8.63E-36	75.80669	Up-Regulated
EFEMP2	1.624765	9.190373	14.86142	7.87E-38	1.44E-35	75.26209	Up-Regulated
BOC	2.645332	7.970428	14.83827	9.60E-38	1.74E-35	75.06482	Up-Regulated
MEOX2	3.041042	4.625206	14.79563	1.39E-37	2.49E-35	74.70141	Up-Regulated
ABCC9	2.409077	7.754972	14.79501	1.39E-37	2.49E-35	74.69617	Up-Regulated
RSPO3	3.028306	6.931391	14.78816	1.48E-37	2.61E-35	74.63777	Up-Regulated
RECK	1.689473	7.320245	14.77719	1.62E-37	2.85E-35	74.54433	Up-Regulated
ENOX1	2.140309	5.031744	14.76804	1.76E-37	3.04E-35	74.46635	Up-Regulated
GIMAP1	1.605259	6.151701	14.76767	1.76E-37	3.04E-35	74.46321	Up-Regulated
FAM180A	2.685692	4.379595	14.75416	1.98E-37	3.36E-35	74.34819	Up-Regulated
LAYN	1.830564	6.570599	14.74857	2.08E-37	3.49E-35	74.30053	Up-Regulated
LAIR1	1.658811	8.601909	14.74422	2.15E-37	3.60E-35	74.26353	Up-Regulated
MMP2	1.928013	12.33847	14.74283	2.18E-37	3.61E-35	74.25172	Up-Regulated
GFPT2	2.356446	7.105944	14.72597	2.52E-37	4.12E-35	74.10817	Up-Regulated
LHFP	1.639019	8.783869	14.72564	2.53E-37	4.12E-35	74.1053	Up-Regulated
TGFB3	1.941421	7.742715	14.72003	2.65E-37	4.29E-35	74.05755	Up-Regulated
SFRP2	4.178496	9.726509	14.7099	2.89E-37	4.64E-35	73.97135	Up-Regulated
HSD11B1	2.340493	5.519007	14.70209	3.09E-37	4.92E-35	73.90489	Up-Regulated
BICC1	2.453147	6.854276	14.69618	3.26E-37	5.14E-35	73.85457	Up-Regulated
CSF1R	1.801332	9.620885	14.64083	5.24E-37	8.21E-35	73.38363	Up-Regulated
KCNMA1	3.420211	8.223047	14.63532	5.49E-37	8.54E-35	73.33675	Up-Regulated
PODN	2.375306	9.563415	14.61791	6.37E-37	9.84E-35	73.18871	Up-Regulated
COL14A1	2.494573	9.723183	14.60922	6.87E-37	1.04E-34	73.11478	Up-Regulated
VGLL3	2.147835	7.586233	14.60918	6.87E-37	1.04E-34	73.11446	Up-Regulated
ITGA11	2.099536	9.159962	14.59956	7.46E-37	1.12E-34	73.03263	Up-Regulated
PTGER3	2.694574	6.364797	14.59481	7.77E-37	1.16E-34	72.99223	Up-Regulated
AOC3	2.412222	9.16374	14.59211	7.95E-37	1.17E-34	72.96928	Up-Regulated
ZCCHC24	1.950332	9.245077	14.58552	8.42E-37	1.23E-34	72.91329	Up-Regulated
MEF2C	1.594901	8.994186	14.57558	9.17E-37	1.33E-34	72.82882	Up-Regulated
PCOLCE	1.699943	9.975264	14.55493	1.09E-36	1.56E-34	72.65331	Up-Regulated
PDCD1LG2	1.81717	5.788582	14.53917	1.25E-36	1.77E-34	72.51936	Up-Regulated

COL1A2	1.885917	14.81976	14.51075	1.60E-36	2.25E-34	72.27799	Up-Regulated
EDNRA	1.656084	8.56179	14.48846	1.93E-36	2.70E-34	72.08868	Up-Regulated
C10orf128	1.923805	4.01064	14.46363	2.39E-36	3.32E-34	71.87784	Up-Regulated
ODZ4	2.477302	6.932518	14.46075	2.45E-36	3.38E-34	71.85345	Up-Regulated
NHSL2	2.246104	5.77478	14.45165	2.65E-36	3.63E-34	71.77618	Up-Regulated
FAM26E	1.728052	6.599056	14.4184	3.53E-36	4.76E-34	71.49399	Up-Regulated
PRKD1	2.001557	5.886453	14.41695	3.57E-36	4.79E-34	71.48171	Up-Regulated
P2RX7	1.96738	5.725559	14.41587	3.60E-36	4.80E-34	71.47259	Up-Regulated
PCDHGB7	1.940957	6.912565	14.39436	4.33E-36	5.74E-34	71.29011	Up-Regulated
GPC6	1.861514	8.62745	14.373	5.20E-36	6.76E-34	71.10897	Up-Regulated
TWIST2	2.21906	4.949897	14.36312	5.66E-36	7.31E-34	71.02521	Up-Regulated
STON1	2.235281	8.160761	14.35338	6.15E-36	7.89E-34	70.94258	Up-Regulated
GREM1	3.046652	11.75777	14.34066	6.86E-36	8.74E-34	70.83477	Up-Regulated
SLC9A9	1.851401	6.457422	14.33385	7.27E-36	9.16E-34	70.77706	Up-Regulated
CLEC11A	1.656761	7.648016	14.32008	8.18E-36	1.02E-33	70.66036	Up-Regulated
MEIS3	1.73917	6.319151	14.29104	1.05E-35	1.29E-33	70.41437	Up-Regulated
SYNE1	2.023807	9.461588	14.27088	1.25E-35	1.50E-33	70.24359	Up-Regulated
FGF14	2.678211	5.377505	14.25291	1.45E-35	1.74E-33	70.09142	Up-Regulated
JAM2	2.093147	6.711058	14.24643	1.53E-35	1.83E-33	70.03655	Up-Regulated
CYP1B1	3.032275	8.247889	14.2437	1.57E-35	1.86E-33	70.01347	Up-Regulated
THBS2	2.501121	10.98939	14.23724	1.66E-35	1.96E-33	69.9588	Up-Regulated
MRC2	1.621507	10.62121	14.23157	1.74E-35	2.04E-33	69.91077	Up-Regulated
ATP8B2	1.725934	8.894217	14.22028	1.92E-35	2.23E-33	69.81521	Up-Regulated
ZFPM2	2.249233	6.058207	14.17838	2.74E-35	3.15E-33	69.46075	Up-Regulated
TLR7	2.178257	6.080035	14.17296	2.87E-35	3.26E-33	69.41495	Up-Regulated
CYSLTR1	1.951913	5.02296	14.17167	2.91E-35	3.28E-33	69.40399	Up-Regulated
C3AR1	1.789553	7.928795	14.1513	3.46E-35	3.88E-33	69.23178	Up-Regulated
MSRB3	2.202371	8.99109	14.1482	3.55E-35	3.96E-33	69.20555	Up-Regulated
FGF7	2.388484	7.863372	14.14267	3.72E-35	4.13E-33	69.15881	Up-Regulated
NAP1L3	2.090479	4.512653	14.13184	4.08E-35	4.50E-33	69.06728	Up-Regulated
RNF150	2.785757	5.874659	14.12826	4.21E-35	4.62E-33	69.03696	Up-Regulated
DPT	2.97799	6.032391	14.12588	4.29E-35	4.69E-33	69.01689	Up-Regulated
EFEMP1	2.296889	9.799738	14.11975	4.52E-35	4.89E-33	68.9651	Up-Regulated
MPDZ	1.982858	7.819772	14.09812	5.44E-35	5.85E-33	68.78229	Up-Regulated
NOX4	1.869074	5.571147	14.09628	5.53E-35	5.91E-33	68.76678	Up-Regulated
MS4A6A	1.698725	8.803264	14.09189	5.74E-35	6.10E-33	68.72963	Up-Regulated
FAM49A	1.768528	6.26975	14.05958	7.56E-35	7.96E-33	68.45681	Up-Regulated
ADAMTS10	1.904092	6.478517	14.0527	8.01E-35	8.39E-33	68.39872	Up-Regulated
HMCN1	2.488179	7.921181	14.04636	8.46E-35	8.81E-33	68.3452	Up-Regulated
CYBB	1.759693	9.899532	14.04104	8.85E-35	9.13E-33	68.30027	Up-Regulated
LSP1	1.588606	9.971782	14.03797	9.08E-35	9.33E-33	68.27437	Up-Regulated
MNDA	1.750705	7.118698	14.02385	1.02E-34	1.05E-32	68.15516	Up-Regulated
ITGAM	1.86626	7.392547	14.02077	1.05E-34	1.07E-32	68.1292	Up-Regulated

HSPB2	1.901304	5.176273	13.99947	1.26E-34	1.28E-32	67.94941	Up-Regulated
ROR2	2.45127	7.644201	13.97828	1.51E-34	1.52E-32	67.77067	Up-Regulated
PRAM1	1.707897	5.098438	13.96851	1.64E-34	1.64E-32	67.68832	Up-Regulated
KIAA1755	1.863271	7.156064	13.95704	1.81E-34	1.80E-32	67.59154	Up-Regulated
COL5A1	1.695926	12.54286	13.94442	2.01E-34	1.99E-32	67.48519	Up-Regulated
THBS1	1.831171	12.64634	13.92875	2.30E-34	2.26E-32	67.35313	Up-Regulated
THBS4	4.770719	7.313552	13.92716	2.33E-34	2.28E-32	67.33972	Up-Regulated
ABCA6	2.614845	5.381233	13.92107	2.46E-34	2.39E-32	67.28838	Up-Regulated
MAP1A	1.843566	9.015708	13.91316	2.63E-34	2.55E-32	67.2217	Up-Regulated
LY86	1.881433	6.418033	13.90849	2.73E-34	2.64E-32	67.18242	Up-Regulated
SFMBT2	1.863649	5.837402	13.88885	3.23E-34	3.10E-32	67.01697	Up-Regulated
LOC399959	2.563311	7.138298	13.8878	3.26E-34	3.11E-32	67.00812	Up-Regulated
LTBP2	1.724676	11.06728	13.87898	3.51E-34	3.34E-32	66.93384	Up-Regulated
ITGB2	1.808784	10.07025	13.87861	3.53E-34	3.34E-32	66.93067	Up-Regulated
CSF2RB	1.821205	8.641466	13.86604	3.92E-34	3.68E-32	66.82483	Up-Regulated
MS4A4A	1.815333	7.283468	13.86154	4.08E-34	3.80E-32	66.78694	Up-Regulated
CPXM2	2.58842	8.163097	13.81415	6.10E-34	5.61E-32	66.3881	Up-Regulated
TAGLN	2.520279	12.45192	13.8111	6.26E-34	5.73E-32	66.36248	Up-Regulated
MS4A7	1.686873	8.36594	13.78572	7.76E-34	7.05E-32	66.14902	Up-Regulated
COPZ2	1.85037	6.159355	13.74774	1.07E-33	9.60E-32	65.82971	Up-Regulated
CLIP3	1.933292	8.570751	13.74574	1.09E-33	9.73E-32	65.81288	Up-Regulated
PLXNC1	2.283877	7.552424	13.74053	1.14E-33	1.01E-31	65.76911	Up-Regulated
MSR1	1.839525	8.550891	13.71779	1.38E-33	1.22E-31	65.57807	Up-Regulated
SIRPB2	1.732087	5.198561	13.71561	1.41E-33	1.23E-31	65.55974	Up-Regulated
PPAPDC3	1.798339	4.995615	13.70322	1.56E-33	1.36E-31	65.45569	Up-Regulated
FXYD6	2.008259	8.347345	13.6725	2.03E-33	1.75E-31	65.19779	Up-Regulated
ACTA2	2.10583	12.50923	13.66304	2.20E-33	1.89E-31	65.1184	Up-Regulated
ADAMTS2	2.074992	9.056384	13.65498	2.35E-33	2.00E-31	65.05077	Up-Regulated
SCARF2	1.793539	8.858171	13.61687	3.25E-33	2.75E-31	64.73102	Up-Regulated
SVEP1	2.287798	8.06474	13.60646	3.55E-33	3.00E-31	64.6438	Up-Regulated
JAKMIP2	2.290257	4.052132	13.58459	4.27E-33	3.58E-31	64.46039	Up-Regulated
LAMA2	2.283692	8.265864	13.56664	4.97E-33	4.14E-31	64.31005	Up-Regulated
REM1	1.74206	5.033023	13.55336	5.56E-33	4.60E-31	64.19873	Up-Regulated
PRELP	3.177605	9.021059	13.54785	5.82E-33	4.80E-31	64.15258	Up-Regulated
LGI2	2.241581	7.16311	13.54136	6.15E-33	5.05E-31	64.09827	Up-Regulated
KAL1	2.000221	7.449725	13.5274	6.92E-33	5.66E-31	63.98134	Up-Regulated
NRK	3.251795	4.274239	13.51999	7.37E-33	6.00E-31	63.91931	Up-Regulated
SIGLEC7	1.705724	5.420067	13.47829	1.05E-32	8.47E-31	63.5703	Up-Regulated
FPR3	1.80826	8.507273	13.47643	1.06E-32	8.57E-31	63.55476	Up-Regulated
SPOCK1	2.826756	8.303459	13.4693	1.13E-32	9.03E-31	63.49515	Up-Regulated
MGP	2.482542	10.94288	13.46385	1.18E-32	9.42E-31	63.44954	Up-Regulated
CDO1	2.637653	3.760013	13.44533	1.38E-32	1.10E-30	63.29468	Up-Regulated
FCGR2C	1.893738	5.454195	13.43727	1.48E-32	1.17E-30	63.22729	Up-Regulated

SIGLEC9	1.740066	5.029659	13.42793	1.60E-32	1.26E-30	63.14924	Up-Regulated
WISP1	1.909359	7.11459	13.41519	1.78E-32	1.39E-30	63.04273	Up-Regulated
CD33	1.606518	5.149386	13.41199	1.83E-32	1.42E-30	63.016	Up-Regulated
S1PR3	1.770811	9.124039	13.39578	2.10E-32	1.62E-30	62.88062	Up-Regulated
NRP2	1.627142	9.815212	13.37367	2.53E-32	1.93E-30	62.69596	Up-Regulated
TREM2	1.939156	6.967461	13.36675	2.69E-32	2.04E-30	62.63819	Up-Regulated
MXRA8	1.915558	10.56317	13.36131	2.81E-32	2.13E-30	62.59272	Up-Regulated
IL10RA	1.717666	8.790342	13.32642	3.77E-32	2.80E-30	62.30156	Up-Regulated
CECR1	1.735578	10.07391	13.31274	4.23E-32	3.11E-30	62.18746	Up-Regulated
BTK	1.983174	6.444443	13.30845	4.39E-32	3.22E-30	62.15167	Up-Regulated
HTRA3	1.817969	9.907029	13.28057	5.55E-32	4.04E-30	61.91927	Up-Regulated
CYP7B1	2.011071	5.547411	13.27491	5.82E-32	4.22E-30	61.87208	Up-Regulated
CPZ	2.009834	7.799904	13.25289	7.00E-32	5.06E-30	61.68859	Up-Regulated
ZNF423	1.876473	7.355238	13.24774	7.31E-32	5.27E-30	61.64568	Up-Regulated
LILRB1	1.701867	6.620213	13.23076	8.44E-32	6.01E-30	61.50428	Up-Regulated
PILRA	1.670175	6.454501	13.21988	9.24E-32	6.54E-30	61.41373	Up-Regulated
NCKAP1L	1.849635	8.495297	13.20456	1.05E-31	7.42E-30	61.28619	Up-Regulated
HAND2	3.90055	5.686791	13.20197	1.07E-31	7.55E-30	61.26459	Up-Regulated
OLFML3	1.760061	8.910713	13.19383	1.15E-31	8.06E-30	61.19688	Up-Regulated
IGFBP5	1.846469	13.02422	13.18361	1.25E-31	8.75E-30	61.11181	Up-Regulated
MRVI1	2.010712	9.604103	13.18092	1.28E-31	8.92E-30	61.08946	Up-Regulated
DOCK2	1.877859	8.382099	13.1491	1.67E-31	1.15E-29	60.82487	Up-Regulated
FCGR2B	2.009041	6.342351	13.10947	2.34E-31	1.58E-29	60.49552	Up-Regulated
FERMT2	1.739985	9.712821	13.10907	2.34E-31	1.58E-29	60.49216	Up-Regulated
MRGPRF	2.418542	8.049594	13.09159	2.71E-31	1.83E-29	60.34701	Up-Regulated
CXCL12	1.922755	9.198328	13.09007	2.75E-31	1.84E-29	60.3344	Up-Regulated
FGL2	2.074353	9.586337	13.08331	2.91E-31	1.94E-29	60.27824	Up-Regulated
SIGLEC1	2.063126	8.027174	13.07222	3.19E-31	2.12E-29	60.18623	Up-Regulated
SYT11	1.607626	8.610022	13.05797	3.60E-31	2.36E-29	60.0679	Up-Regulated
RASSF2	1.643906	8.942919	13.02748	4.64E-31	3.01E-29	59.81502	Up-Regulated
ZNF385D	2.367418	3.823866	13.00611	5.55E-31	3.56E-29	59.63784	Up-Regulated
GLI1	2.162493	6.00615	12.9976	5.96E-31	3.81E-29	59.5673	Up-Regulated
CYBRD1	1.858257	10.60529	12.99321	6.18E-31	3.94E-29	59.53093	Up-Regulated
PRDM6	2.068975	4.472263	12.98166	6.81E-31	4.32E-29	59.43523	Up-Regulated
LILRB4	1.914655	7.659359	12.97688	7.09E-31	4.47E-29	59.39562	Up-Regulated
GDF6	2.458437	2.932838	12.97182	7.40E-31	4.62E-29	59.35368	Up-Regulated
DPEP2	1.594507	5.075116	12.96098	8.10E-31	5.04E-29	59.26392	Up-Regulated
PLN	3.065506	7.074001	12.95737	8.35E-31	5.18E-29	59.23405	Up-Regulated
ODZ3	2.92777	6.279927	12.95653	8.41E-31	5.20E-29	59.22706	Up-Regulated
TNS1	2.080409	12.10837	12.92273	1.11E-30	6.79E-29	58.94729	Up-Regulated
MYLK	2.440526	11.51189	12.91832	1.16E-30	7.03E-29	58.91077	Up-Regulated
ESR1	1.854495	4.597475	12.91572	1.18E-30	7.16E-29	58.88925	Up-Regulated
DCLK2	1.897999	5.690504	12.9146	1.19E-30	7.21E-29	58.87998	Up-Regulated

CD84	2.038642	6.886297	12.8907	1.46E-30	8.72E-29	58.68237	Up-Regulated
SSPN	1.871362	8.652358	12.88557	1.52E-30	9.08E-29	58.63995	Up-Regulated
C17orf87	1.901426	5.318463	12.88361	1.55E-30	9.20E-29	58.62372	Up-Regulated
PIK3R5	1.664253	7.307908	12.86869	1.75E-30	1.04E-28	58.50042	Up-Regulated
PDZRN4	3.188046	3.842102	12.85731	1.93E-30	1.14E-28	58.40633	Up-Regulated
ARHGAP20	1.958909	5.215594	12.85649	1.94E-30	1.14E-28	58.39961	Up-Regulated
RBMS3	1.915567	5.162668	12.85196	2.01E-30	1.18E-28	58.36215	Up-Regulated
FCN1	2.122986	4.986768	12.85167	2.02E-30	1.18E-28	58.35976	Up-Regulated
LRCH2	1.73079	5.464023	12.83782	2.26E-30	1.32E-28	58.24539	Up-Regulated
DCLK1	2.573374	5.92393	12.82169	2.59E-30	1.50E-28	58.11213	Up-Regulated
FAM124B	1.587592	4.792707	12.81472	2.75E-30	1.59E-28	58.05466	Up-Regulated
HRH2	1.604677	4.806355	12.7861	3.49E-30	1.99E-28	57.81849	Up-Regulated
MPEG1	1.757905	9.570716	12.78506	3.52E-30	2.01E-28	57.80984	Up-Regulated
PRKG1	1.752372	6.415052	12.77084	3.96E-30	2.24E-28	57.69258	Up-Regulated
TMEM47	1.68017	9.028223	12.76444	4.17E-30	2.36E-28	57.63984	Up-Regulated
SORCS2	2.197623	5.881886	12.73544	5.31E-30	2.99E-28	57.40082	Up-Regulated
ZNF135	1.800558	4.837764	12.71455	6.32E-30	3.55E-28	57.2287	Up-Regulated
TXLNB	1.918857	4.429054	12.69875	7.21E-30	4.03E-28	57.09861	Up-Regulated
SLAMF8	1.626746	8.639164	12.68093	8.36E-30	4.64E-28	56.95194	Up-Regulated
CILP	3.348228	6.401693	12.67733	8.62E-30	4.77E-28	56.9223	Up-Regulated
RTN1	1.98662	6.12168	12.67588	8.72E-30	4.81E-28	56.91033	Up-Regulated
FN1	2.052495	14.41243	12.65991	9.96E-30	5.48E-28	56.77901	Up-Regulated
C1orf162	1.593292	7.645931	12.64285	1.15E-29	6.30E-28	56.63865	Up-Regulated
EBF3	1.856194	5.117072	12.6018	1.61E-29	8.83E-28	56.30134	Up-Regulated
RNLS	1.779549	4.41913	12.58192	1.90E-29	1.03E-27	56.1381	Up-Regulated
CSF2RA	1.742288	6.567139	12.55509	2.38E-29	1.28E-27	55.91787	Up-Regulated
NAV3	2.217351	5.00035	12.50648	3.55E-29	1.90E-27	55.51932	Up-Regulated
P4HA3	1.75429	5.512561	12.50323	3.65E-29	1.94E-27	55.49268	Up-Regulated
MOXD1	2.156743	7.847533	12.49422	3.93E-29	2.09E-27	55.41886	Up-Regulated
EPB41L3	1.592329	8.117335	12.49273	3.98E-29	2.11E-27	55.40667	Up-Regulated
ISM1	2.771066	5.608344	12.49093	4.04E-29	2.13E-27	55.39187	Up-Regulated
CHRDL1	3.847458	5.790138	12.48803	4.14E-29	2.18E-27	55.36814	Up-Regulated
CFH	1.640735	10.14836	12.46674	4.94E-29	2.58E-27	55.19382	Up-Regulated
CPXM1	1.906223	7.932708	12.45881	5.27E-29	2.75E-27	55.12893	Up-Regulated
RGMA	2.928274	8.432403	12.45013	5.66E-29	2.95E-27	55.05789	Up-Regulated
THSD7A	1.818618	5.349523	12.43272	6.54E-29	3.39E-27	54.91543	Up-Regulated
INMT	2.133514	6.790068	12.41987	7.27E-29	3.76E-27	54.81033	Up-Regulated
FAM20A	1.671566	6.790998	12.41826	7.37E-29	3.80E-27	54.79715	Up-Regulated
FCGR1A	1.696517	6.188918	12.41053	7.85E-29	4.04E-27	54.73396	Up-Regulated
LPPR4	1.92918	6.191569	12.40551	8.19E-29	4.18E-27	54.69293	Up-Regulated
FBLN5	1.70095	9.507128	12.39533	8.90E-29	4.52E-27	54.60975	Up-Regulated
LIMS2	1.922834	8.616844	12.38505	9.69E-29	4.89E-27	54.52578	Up-Regulated
ANGPTL1	2.992613	5.539226	12.37342	1.07E-28	5.35E-27	54.43073	Up-Regulated

NLRP3	1.644553	5.507746	12.36905	1.11E-28	5.51E-27	54.39502	Up-Regulated
SPARCL1	1.982275	11.63987	12.36841	1.11E-28	5.53E-27	54.38979	Up-Regulated
VSIG4	1.834101	7.816343	12.3622	1.17E-28	5.79E-27	54.33909	Up-Regulated
CD53	1.607195	9.356438	12.36194	1.17E-28	5.79E-27	54.33697	Up-Regulated
GHR	2.09224	5.623657	12.35419	1.25E-28	6.15E-27	54.27375	Up-Regulated
DOCK10	1.61973	8.028859	12.33536	1.46E-28	7.17E-27	54.12006	Up-Regulated
PDE1B	1.926838	5.836922	12.33396	1.48E-28	7.24E-27	54.10863	Up-Regulated
DHH	1.693508	2.991941	12.33228	1.50E-28	7.32E-27	54.0949	Up-Regulated
ARHGAP15	1.614269	6.482954	12.31329	1.75E-28	8.52E-27	53.94006	Up-Regulated
MAPK10	2.12594	5.556971	12.31243	1.76E-28	8.56E-27	53.93305	Up-Regulated
FMO1	2.489372	4.882605	12.29064	2.11E-28	1.02E-26	53.75536	Up-Regulated
ATP10A	1.606818	7.416745	12.28656	2.18E-28	1.05E-26	53.72213	Up-Regulated
C13orf33	2.037931	6.854821	12.28635	2.19E-28	1.05E-26	53.7204	Up-Regulated
NCAM2	2.603616	3.655278	12.2769	2.36E-28	1.13E-26	53.64347	Up-Regulated
CCDC8	2.134477	6.179862	12.27285	2.44E-28	1.17E-26	53.61048	Up-Regulated
OGN	3.598746	7.197985	12.26985	2.50E-28	1.20E-26	53.58603	Up-Regulated
ADAMTSL3	2.38157	6.188672	12.25676	2.79E-28	1.33E-26	53.47944	Up-Regulated
MYL9	2.269252	12.19617	12.24265	3.13E-28	1.48E-26	53.36459	Up-Regulated
DSEL	1.771628	6.640433	12.22586	3.59E-28	1.69E-26	53.22793	Up-Regulated
TCF21	1.649719	7.402881	12.19626	4.58E-28	2.13E-26	52.98726	Up-Regulated
TMEM130	2.618622	4.424371	12.19132	4.77E-28	2.21E-26	52.94706	Up-Regulated
CALD1	1.782847	12.98533	12.19099	4.79E-28	2.21E-26	52.94441	Up-Regulated
SV2B	2.198033	4.843342	12.15715	6.32E-28	2.88E-26	52.66952	Up-Regulated
PTGFR	1.99718	5.502963	12.13099	7.83E-28	3.52E-26	52.45712	Up-Regulated
ADCY2	2.623965	4.725146	12.12304	8.36E-28	3.75E-26	52.39268	Up-Regulated
GEFT	1.988041	7.16738	12.11082	9.24E-28	4.12E-26	52.29352	Up-Regulated
DACT3	2.135025	7.256679	12.09595	1.04E-27	4.59E-26	52.173	Up-Regulated
RGAG4	1.743153	6.307753	12.08633	1.13E-27	4.95E-26	52.09501	Up-Regulated
MFAP4	2.315297	10.58075	12.07718	1.22E-27	5.33E-26	52.02085	Up-Regulated
GALNTL2	2.11799	5.707648	12.07134	1.28E-27	5.55E-26	51.97356	Up-Regulated
DACT1	1.802793	8.088534	12.06012	1.40E-27	6.06E-26	51.88266	Up-Regulated
NNMT	1.651908	9.606581	12.04732	1.55E-27	6.70E-26	51.77904	Up-Regulated
COL10A1	3.168311	8.247438	12.04525	1.58E-27	6.80E-26	51.76225	Up-Regulated
SLC24A3	1.917669	7.603784	12.04281	1.61E-27	6.92E-26	51.74249	Up-Regulated
ABI3BP	2.682949	8.297054	12.03628	1.70E-27	7.29E-26	51.68964	Up-Regulated
FAM70A	1.999095	5.335899	12.02607	1.85E-27	7.90E-26	51.60708	Up-Regulated
CD163	1.764069	8.731156	12.02428	1.88E-27	8.00E-26	51.59256	Up-Regulated
TCEAL7	1.685359	4.45344	12.01803	1.97E-27	8.41E-26	51.54199	Up-Regulated
NCF1C	2.143867	5.433273	12.01273	2.06E-27	8.76E-26	51.49919	Up-Regulated
NPR1	1.633815	6.737874	12.00933	2.12E-27	8.99E-26	51.47168	Up-Regulated
ITGA9	2.015844	7.509405	12.00092	2.27E-27	9.59E-26	51.40368	Up-Regulated
LTBP1	1.592032	10.66616	11.9904	2.47E-27	1.04E-25	51.31865	Up-Regulated
ADAMTSL1	1.841215	6.465111	11.98711	2.54E-27	1.06E-25	51.29206	Up-Regulated

ZNF454	1.65498	3.192507	11.9624	3.11E-27	1.29E-25	51.09248	Up-Regulated
FBLN1	2.455843	10.98115	11.96191	3.12E-27	1.29E-25	51.08852	Up-Regulated
EPHA3	2.044814	7.4206	11.95635	3.27E-27	1.35E-25	51.04362	Up-Regulated
KCNN3	1.658807	6.143221	11.9542	3.32E-27	1.37E-25	51.02622	Up-Regulated
LRRK2	2.014145	6.90218	11.94281	3.65E-27	1.49E-25	50.93428	Up-Regulated
TLL1	1.966069	4.440223	11.93317	3.95E-27	1.61E-25	50.85654	Up-Regulated
NTM	1.944663	6.534075	11.92526	4.21E-27	1.71E-25	50.7927	Up-Regulated
NBLA00301	3.49996	4.358577	11.91358	4.63E-27	1.88E-25	50.69851	Up-Regulated
TNFSF8	1.812738	4.923013	11.86444	6.90E-27	2.78E-25	50.30256	Up-Regulated
GGTA1	1.626885	6.041813	11.84377	8.17E-27	3.26E-25	50.13614	Up-Regulated
SIGLEC10	1.746536	7.597259	11.83883	8.50E-27	3.38E-25	50.09641	Up-Regulated
C1QC	1.635986	11.21269	11.82992	9.14E-27	3.63E-25	50.02474	Up-Regulated
CD180	1.848541	6.707446	11.82205	9.75E-27	3.85E-25	49.9615	Up-Regulated
C7orf58	2.116276	8.256875	11.8081	1.09E-26	4.31E-25	49.84931	Up-Regulated
CCR2	2.058863	5.039073	11.80469	1.12E-26	4.42E-25	49.82189	Up-Regulated
CD37	1.796452	8.94497	11.8015	1.15E-26	4.52E-25	49.79627	Up-Regulated
CELF2	1.688578	9.049296	11.7859	1.31E-26	5.09E-25	49.67092	Up-Regulated
NEXN	2.007245	8.768199	11.77585	1.42E-26	5.51E-25	49.59024	Up-Regulated
PEG3	2.196081	5.41316	11.7605	1.61E-26	6.23E-25	49.46699	Up-Regulated
ZFHX4	2.268827	6.798243	11.74053	1.89E-26	7.27E-25	49.30678	Up-Regulated
PTGIS	3.030834	8.420149	11.72519	2.14E-26	8.21E-25	49.18372	Up-Regulated
SIGLEC5	1.841654	4.708069	11.71838	2.26E-26	8.66E-25	49.12914	Up-Regulated
LMOD1	2.746274	9.187756	11.71808	2.27E-26	8.66E-25	49.12673	Up-Regulated
OLR1	1.963637	6.736899	11.71052	2.41E-26	9.19E-25	49.06617	Up-Regulated
ABCA9	2.483286	4.828665	11.69793	2.67E-26	1.01E-24	48.96531	Up-Regulated
LILRA2	1.668261	3.489075	11.67878	3.11E-26	1.18E-24	48.81192	Up-Regulated
FLNA	1.857601	14.53566	11.67132	3.31E-26	1.25E-24	48.75223	Up-Regulated
KLHL6	1.707313	7.624232	11.66533	3.47E-26	1.31E-24	48.70426	Up-Regulated
COL1A1	1.790352	15.8063	11.65222	3.86E-26	1.45E-24	48.59938	Up-Regulated
RSPO1	2.367687	3.13529	11.65011	3.93E-26	1.47E-24	48.5825	Up-Regulated
GAPT	2.117444	5.086066	11.6404	4.25E-26	1.58E-24	48.50485	Up-Regulated
IGF1	2.538079	8.2934	11.63016	4.61E-26	1.71E-24	48.42297	Up-Regulated
HHIPL1	1.600357	5.973911	11.6243	4.84E-26	1.78E-24	48.37616	Up-Regulated
EVI2B	1.641685	9.210376	11.62415	4.84E-26	1.78E-24	48.37497	Up-Regulated
FLJ42709	1.621518	5.505366	11.59264	6.24E-26	2.29E-24	48.12331	Up-Regulated
NUDT11	1.744784	3.684152	11.5742	7.25E-26	2.64E-24	47.97611	Up-Regulated
PLXNA4	2.446938	6.502522	11.55545	8.43E-26	3.06E-24	47.8266	Up-Regulated
CADM3	3.083561	4.593674	11.55531	8.44E-26	3.06E-24	47.82543	Up-Regulated
ST8SIA1	1.620656	5.489311	11.55124	8.72E-26	3.15E-24	47.79299	Up-Regulated
C14orf132	2.001997	7.738308	11.54997	8.81E-26	3.18E-24	47.78284	Up-Regulated
SMOC2	2.339604	9.463396	11.54553	9.13E-26	3.29E-24	47.74745	Up-Regulated
FGFR1	1.63488	9.757596	11.54048	9.51E-26	3.42E-24	47.70723	Up-Regulated
MRC1	1.927174	7.976898	11.53813	9.69E-26	3.47E-24	47.68853	Up-Regulated

CSDC2	2.080113	5.005663	11.53808	9.69E-26	3.47E-24	47.68808	Up-Regulated
POSTN	1.652913	11.78198	11.5213	1.11E-25	3.94E-24	47.5544	Up-Regulated
WDFY4	2.177212	7.674417	11.46854	1.70E-25	5.95E-24	47.13456	Up-Regulated
ANK2	2.295847	7.211819	11.46844	1.70E-25	5.95E-24	47.13378	Up-Regulated
PLA2G4C	1.638133	6.424518	11.468	1.70E-25	5.96E-24	47.1303	Up-Regulated
PRKCB	1.984208	8.236207	11.44003	2.13E-25	7.40E-24	46.90804	Up-Regulated
PLEK	1.643737	8.567214	11.43095	2.29E-25	7.91E-24	46.83593	Up-Regulated
NALCN	2.401672	4.06285	11.4191	2.52E-25	8.66E-24	46.7418	Up-Regulated
EVC2	1.821704	5.191434	11.41752	2.55E-25	8.76E-24	46.72928	Up-Regulated
CR1	2.394256	6.006682	11.40588	2.80E-25	9.60E-24	46.63696	Up-Regulated
EFS	1.656458	6.446302	11.37593	3.57E-25	1.22E-23	46.39946	Up-Regulated
NAPSB	2.066673	5.718705	11.37378	3.63E-25	1.24E-23	46.38241	Up-Regulated
KERA	2.030135	2.113155	11.36857	3.78E-25	1.29E-23	46.34109	Up-Regulated
CNN1	3.161872	9.83063	11.35836	4.10E-25	1.39E-23	46.26021	Up-Regulated
GSTM5	2.022075	4.945928	11.34732	4.48E-25	1.52E-23	46.17284	Up-Regulated
MMP16	1.723032	4.937303	11.34232	4.67E-25	1.58E-23	46.13319	Up-Regulated
HSPB7	3.167647	7.240352	11.3181	5.66E-25	1.90E-23	45.94156	Up-Regulated
DZIP1	1.622072	7.030779	11.29339	6.90E-25	2.30E-23	45.74624	Up-Regulated
LSAMP	1.824433	6.576555	11.28958	7.11E-25	2.37E-23	45.71614	Up-Regulated
FAM43B	1.85721	2.867737	11.28508	7.37E-25	2.45E-23	45.68059	Up-Regulated
HS3ST3A1	1.613649	5.651092	11.28489	7.39E-25	2.45E-23	45.67903	Up-Regulated
MAP6	1.939072	5.763075	11.27859	7.77E-25	2.57E-23	45.62929	Up-Regulated
GNAO1	2.495316	7.133408	11.26735	8.50E-25	2.81E-23	45.5405	Up-Regulated
AOAH	1.875037	6.693579	11.24508	1.01E-24	3.34E-23	45.36478	Up-Regulated
FCGR3A	1.730334	9.531993	11.24371	1.03E-24	3.38E-23	45.35397	Up-Regulated
NUDT10	1.89161	2.829725	11.24234	1.04E-24	3.41E-23	45.34318	Up-Regulated
GXYLT2	2.080186	6.054178	11.23842	1.07E-24	3.50E-23	45.31224	Up-Regulated
F13A1	2.410027	7.637861	11.21076	1.33E-24	4.31E-23	45.09416	Up-Regulated
KLHL4	1.975755	3.692965	11.18252	1.67E-24	5.35E-23	44.87177	Up-Regulated
LOC339524	1.788694	5.061633	11.17639	1.75E-24	5.61E-23	44.82355	Up-Regulated
P2RY13	1.71335	6.210378	11.17388	1.79E-24	5.71E-23	44.80383	Up-Regulated
PPFIA2	1.753569	2.746126	11.1503	2.16E-24	6.84E-23	44.61832	Up-Regulated
BHMT2	2.06517	4.43427	11.14233	2.30E-24	7.27E-23	44.55569	Up-Regulated
PNMAL2	1.600575	4.963567	11.13764	2.39E-24	7.54E-23	44.51883	Up-Regulated
EML1	1.599122	8.315614	11.13537	2.43E-24	7.66E-23	44.50103	Up-Regulated
LRRN4CL	1.753096	5.788922	11.12879	2.56E-24	8.05E-23	44.44934	Up-Regulated
PGR	1.99076	5.31458	11.10313	3.14E-24	9.79E-23	44.24783	Up-Regulated
UBE2QL1	1.805701	4.293187	11.09061	3.47E-24	1.08E-22	44.14962	Up-Regulated
SFRP1	3.074451	6.577797	11.06156	4.36E-24	1.34E-22	43.92182	Up-Regulated
RNF180	1.61374	5.739317	11.05725	4.52E-24	1.39E-22	43.88808	Up-Regulated
C1QB	1.625974	11.476	11.05659	4.54E-24	1.39E-22	43.88292	Up-Regulated
TSPAN11	1.620796	7.639717	11.04615	4.93E-24	1.51E-22	43.80116	Up-Regulated
GPR88	1.854373	2.755316	11.04408	5.01E-24	1.53E-22	43.78493	Up-Regulated

TAGAP	1.66015	7.371582	11.01753	6.18E-24	1.88E-22	43.57714	Up-Regulated
ZDHHC15	1.865853	3.91511	10.99257	7.53E-24	2.26E-22	43.38199	Up-Regulated
DKK2	1.993698	5.538737	10.99105	7.62E-24	2.29E-22	43.37012	Up-Regulated
CTHRC1	1.690639	9.083743	10.98365	8.08E-24	2.41E-22	43.31226	Up-Regulated
FAM107A	1.839175	6.581937	10.98206	8.19E-24	2.44E-22	43.29984	Up-Regulated
ADD2	2.268316	3.720255	10.96702	9.22E-24	2.72E-22	43.18235	Up-Regulated
CCDC69	1.586359	9.250497	10.96675	9.24E-24	2.73E-22	43.18025	Up-Regulated
SLIT3	1.922534	8.733657	10.96284	9.53E-24	2.80E-22	43.14976	Up-Regulated
TLR8	1.872561	5.815025	10.96138	9.64E-24	2.83E-22	43.13838	Up-Regulated
LOC728264	2.285592	8.080675	10.95681	9.99E-24	2.93E-22	43.10271	Up-Regulated
NCF1	1.694682	7.123407	10.94403	1.11E-23	3.23E-22	43.00294	Up-Regulated
RASSF8	1.67222	7.758929	10.93876	1.15E-23	3.36E-22	42.96184	Up-Regulated
SELP	2.413305	6.708415	10.93049	1.23E-23	3.57E-22	42.89738	Up-Regulated
ADORA3	1.645143	6.079187	10.9163	1.38E-23	3.97E-22	42.78676	Up-Regulated
LOC145820	1.794278	3.081164	10.91249	1.42E-23	4.09E-22	42.75711	Up-Regulated
LRRC4C	2.016665	3.164979	10.91088	1.44E-23	4.13E-22	42.74454	Up-Regulated
TSPAN2	1.893612	7.47902	10.90973	1.45E-23	4.16E-22	42.73561	Up-Regulated
ADAM23	2.118688	4.766104	10.90814	1.47E-23	4.21E-22	42.7232	Up-Regulated
MAP1B	1.589177	10.10749	10.90002	1.56E-23	4.46E-22	42.65993	Up-Regulated
CPEB1	2.183515	2.721396	10.89442	1.63E-23	4.66E-22	42.61639	Up-Regulated
FOLR2	1.890237	6.809936	10.88729	1.73E-23	4.92E-22	42.56084	Up-Regulated
POPDC2	1.956979	5.476393	10.88553	1.75E-23	4.98E-22	42.54715	Up-Regulated
P2RY8	1.604659	7.154216	10.88477	1.76E-23	5.01E-22	42.54127	Up-Regulated
KCNMB1	2.146587	7.650765	10.87956	1.84E-23	5.21E-22	42.50073	Up-Regulated
GPR133	2.412751	6.391324	10.87599	1.89E-23	5.35E-22	42.4729	Up-Regulated
FAM19A5	1.749684	6.571281	10.86941	1.99E-23	5.62E-22	42.42173	Up-Regulated
C21orf34	2.213321	3.772079	10.8674	2.02E-23	5.70E-22	42.40614	Up-Regulated
SPON1	2.228805	9.655737	10.85498	2.23E-23	6.27E-22	42.3095	Up-Regulated
ADAM33	2.582879	5.906966	10.85357	2.25E-23	6.33E-22	42.2986	Up-Regulated
AR	2.123833	3.71792	10.82989	2.71E-23	7.57E-22	42.11461	Up-Regulated
SOD3	1.731431	10.3231	10.81511	3.05E-23	8.44E-22	41.99988	Up-Regulated
SETBP1	1.780854	7.970556	10.80722	3.24E-23	8.95E-22	41.9386	Up-Regulated
SIGLEC8	2.088686	4.631395	10.78263	3.93E-23	1.08E-21	41.74798	Up-Regulated
AVPR1A	1.673105	4.974109	10.77645	4.13E-23	1.13E-21	41.70007	Up-Regulated
AMPH	1.610187	4.934909	10.76452	4.53E-23	1.23E-21	41.60762	Up-Regulated
P2RY12	1.875503	2.862761	10.75988	4.70E-23	1.27E-21	41.57171	Up-Regulated
FRZB	1.74797	8.223835	10.749	5.12E-23	1.38E-21	41.48748	Up-Regulated
CLEC10A	2.128693	5.424198	10.74621	5.23E-23	1.41E-21	41.46589	Up-Regulated
RASGRP2	1.848618	6.162599	10.73774	5.59E-23	1.50E-21	41.40036	Up-Regulated
PLAC9	1.813778	4.899928	10.73339	5.78E-23	1.55E-21	41.36669	Up-Regulated
ITIH3	2.111872	3.096311	10.72882	5.99E-23	1.60E-21	41.33132	Up-Regulated
CCL19	3.07974	5.889852	10.71729	6.56E-23	1.75E-21	41.24219	Up-Regulated
HOPX	1.940917	8.292951	10.71723	6.56E-23	1.75E-21	41.24174	Up-Regulated

PRICKLE1	1.718853	6.949557	10.69562	7.77E-23	2.07E-21	41.07476	Up-Regulated
CHRD	1.737676	6.657712	10.68482	8.45E-23	2.25E-21	40.99136	Up-Regulated
MATK	1.590782	4.819897	10.68225	8.63E-23	2.29E-21	40.97152	Up-Regulated
PIK3CG	1.753373	7.015842	10.67364	9.22E-23	2.43E-21	40.90512	Up-Regulated
LILRA4	2.019226	2.537425	10.67215	9.33E-23	2.45E-21	40.89358	Up-Regulated
FHL1	2.173743	9.885383	10.64734	1.13E-22	2.97E-21	40.7023	Up-Regulated
CNKSR2	2.428945	2.392307	10.63572	1.24E-22	3.23E-21	40.61274	Up-Regulated
CNTN4	1.686145	6.23101	10.63349	1.26E-22	3.28E-21	40.59553	Up-Regulated
FAT3	2.357729	4.338742	10.61719	1.43E-22	3.72E-21	40.47007	Up-Regulated
IKZF1	1.624007	8.50047	10.61555	1.45E-22	3.76E-21	40.45742	Up-Regulated
GLI2	1.622124	7.753605	10.61492	1.46E-22	3.77E-21	40.45258	Up-Regulated
PCBP3	1.994101	4.809834	10.61252	1.49E-22	3.84E-21	40.43413	Up-Regulated
DARC	2.691315	7.291528	10.59374	1.72E-22	4.43E-21	40.28966	Up-Regulated
FMO2	2.603318	6.880605	10.5908	1.76E-22	4.53E-21	40.26705	Up-Regulated
MDGA1	1.611116	6.373462	10.58318	1.87E-22	4.80E-21	40.20841	Up-Regulated
RGS4	1.936923	6.49465	10.57765	1.95E-22	5.01E-21	40.16592	Up-Regulated
KCNT2	1.743887	3.913668	10.57473	1.99E-22	5.11E-21	40.14352	Up-Regulated
BARX1	2.513774	7.854211	10.57418	2.00E-22	5.12E-21	40.13928	Up-Regulated
PDE3A	2.10956	6.995072	10.57171	2.04E-22	5.21E-21	40.12031	Up-Regulated
SHANK1	1.81116	3.021858	10.56284	2.19E-22	5.57E-21	40.0522	Up-Regulated
CLEC12A	1.717259	4.073651	10.56138	2.21E-22	5.63E-21	40.04094	Up-Regulated
FILIP1	1.721958	6.927993	10.54866	2.44E-22	6.19E-21	39.94334	Up-Regulated
ST6GAL2	2.295638	4.116338	10.54808	2.45E-22	6.21E-21	39.9389	Up-Regulated
CNTN1	3.080802	4.315	10.5294	2.83E-22	7.15E-21	39.79554	Up-Regulated
KCNJ5	1.884132	4.341056	10.52148	3.01E-22	7.59E-21	39.7348	Up-Regulated
HSPB6	3.099642	9.52642	10.50434	3.44E-22	8.63E-21	39.60351	Up-Regulated
SYNPO2	2.857915	10.46682	10.49844	3.60E-22	9.02E-21	39.55832	Up-Regulated
ADAMTS12	1.882466	7.552273	10.48813	3.90E-22	9.76E-21	39.47936	Up-Regulated
CCL14	2.383102	6.110173	10.46936	4.51E-22	1.12E-20	39.33575	Up-Regulated
P2RY14	1.682666	6.092912	10.46328	4.73E-22	1.17E-20	39.28927	Up-Regulated
SYNC	2.128877	7.225365	10.41901	6.66E-22	1.64E-20	38.95108	Up-Regulated
SELL	1.718537	7.953895	10.41718	6.76E-22	1.66E-20	38.9371	Up-Regulated
APOE	1.783518	11.7525	10.39813	7.83E-22	1.92E-20	38.79179	Up-Regulated
KIAA1644	2.175662	6.096715	10.38676	8.54E-22	2.09E-20	38.70512	Up-Regulated
ZNF471	1.680065	4.817216	10.38401	8.73E-22	2.13E-20	38.68421	Up-Regulated
CD48	1.610431	7.939894	10.37653	9.24E-22	2.25E-20	38.6272	Up-Regulated
PGM5	2.64383	7.62196	10.34777	1.15E-21	2.78E-20	38.40828	Up-Regulated
C15orf59	1.813646	3.853886	10.34381	1.19E-21	2.86E-20	38.37815	Up-Regulated
TNC	1.897038	11.25343	10.34336	1.19E-21	2.87E-20	38.37473	Up-Regulated
CASQ2	2.794701	4.468872	10.33789	1.24E-21	2.99E-20	38.33314	Up-Regulated
PDE2A	1.660799	6.82982	10.33748	1.25E-21	2.99E-20	38.33006	Up-Regulated
GPBAR1	1.622002	6.144169	10.31903	1.44E-21	3.43E-20	38.18978	Up-Regulated
KIAA0748	1.662201	4.77516	10.31121	1.53E-21	3.64E-20	38.13041	Up-Regulated

PTPRC	1.589558	9.923165	10.3074	1.57E-21	3.74E-20	38.10148	Up-Regulated
NCF1B	1.920809	4.510253	10.29855	1.68E-21	3.99E-20	38.0343	Up-Regulated
SRPX	1.894903	6.85785	10.28382	1.89E-21	4.46E-20	37.92252	Up-Regulated
C20orf103	2.100359	5.017047	10.28268	1.90E-21	4.48E-20	37.91392	Up-Regulated
TNFRSF9	1.632384	5.267215	10.28046	1.94E-21	4.56E-20	37.89706	Up-Regulated
SCN7A	2.845159	3.577954	10.26965	2.10E-21	4.95E-20	37.81509	Up-Regulated
SHISA3	2.491895	4.640298	10.2667	2.15E-21	5.05E-20	37.79271	Up-Regulated
MN1	1.908342	8.236644	10.26623	2.16E-21	5.06E-20	37.78921	Up-Regulated
DNAJB5	1.60023	7.737611	10.26536	2.17E-21	5.09E-20	37.78258	Up-Regulated
PDZD4	2.147513	5.045309	10.2605	2.26E-21	5.26E-20	37.7458	Up-Regulated
ZNF835	1.841272	3.288684	10.24798	2.48E-21	5.77E-20	37.65094	Up-Regulated
JPH2	2.436291	7.148846	10.23896	2.66E-21	6.18E-20	37.58269	Up-Regulated
C7	3.547149	7.590267	10.23876	2.66E-21	6.18E-20	37.58112	Up-Regulated
DAAM2	1.626065	8.527561	10.23692	2.70E-21	6.26E-20	37.56725	Up-Regulated
CPE	1.802291	8.49719	10.2304	2.84E-21	6.58E-20	37.51789	Up-Regulated
ELN	1.929128	9.767705	10.22871	2.88E-21	6.65E-20	37.50511	Up-Regulated
PNMAL1	2.118564	5.004884	10.22468	2.97E-21	6.85E-20	37.4746	Up-Regulated
KCNK2	2.410725	3.138738	10.22407	2.98E-21	6.87E-20	37.47002	Up-Regulated
TMTC1	1.681104	8.27482	10.22145	3.04E-21	6.99E-20	37.45019	Up-Regulated
PRICKLE2	1.615462	8.288661	10.22001	3.08E-21	7.06E-20	37.43929	Up-Regulated
FAM129A	1.717245	9.931941	10.21914	3.10E-21	7.10E-20	37.43271	Up-Regulated
PLA2G5	1.887159	3.756226	10.20802	3.37E-21	7.71E-20	37.34861	Up-Regulated
HLA-DOA	1.937481	8.523448	10.2067	3.41E-21	7.78E-20	37.33866	Up-Regulated
PDGFRL	2.016088	5.521161	10.20562	3.43E-21	7.83E-20	37.33047	Up-Regulated
CRYAB	1.98897	7.431872	10.19969	3.59E-21	8.18E-20	37.28569	Up-Regulated
BEND6	1.680223	4.595792	10.19224	3.80E-21	8.63E-20	37.2294	Up-Regulated
ACSM5	1.615527	2.15046	10.18734	3.95E-21	8.93E-20	37.19246	Up-Regulated
CHRDL2	2.9606	7.52495	10.18458	4.03E-21	9.11E-20	37.17157	Up-Regulated
PTCH2	2.115582	5.163963	10.17896	4.21E-21	9.49E-20	37.12913	Up-Regulated
NRXN2	2.250455	5.766196	10.16604	4.65E-21	1.04E-19	37.03171	Up-Regulated
ADAM12	1.813087	8.030916	10.16564	4.66E-21	1.04E-19	37.02865	Up-Regulated
CCR4	1.804908	5.002204	10.16168	4.80E-21	1.07E-19	36.99878	Up-Regulated
GPR1	1.808567	3.306711	10.16058	4.84E-21	1.08E-19	36.99051	Up-Regulated
NPAS3	1.712801	3.875534	10.14272	5.55E-21	1.23E-19	36.85591	Up-Regulated
PI16	2.945823	4.683066	10.13652	5.82E-21	1.28E-19	36.80916	Up-Regulated
MYH11	3.171729	12.54622	10.12767	6.23E-21	1.37E-19	36.74251	Up-Regulated
GPRASP1	1.659857	6.458559	10.12049	6.58E-21	1.44E-19	36.68853	Up-Regulated
FABP4	2.576621	4.374357	10.11787	6.71E-21	1.47E-19	36.6688	Up-Regulated
ZNF354C	1.672081	4.145769	10.09754	7.83E-21	1.70E-19	36.5159	Up-Regulated
NTRK3	2.019476	2.92826	10.08948	8.33E-21	1.80E-19	36.45527	Up-Regulated
SCRG1	3.152584	4.315599	10.08431	8.66E-21	1.87E-19	36.41645	Up-Regulated
LOC100192378	1.606163	1.712809	10.05629	1.07E-20	2.30E-19	36.20604	Up-Regulated

PCDHGB6	1.642651	5.567053	10.04871	1.14E-20	2.44E-19	36.14918	Up-Regulated
PCDH10	2.636344	2.942063	10.03166	1.29E-20	2.77E-19	36.02141	Up-Regulated
IQSEC3	1.708009	4.280184	10.01578	1.46E-20	3.11E-19	35.90247	Up-Regulated
C3	2.015722	13.14502	10.00933	1.53E-20	3.27E-19	35.85414	Up-Regulated
NKX3-2	2.320488	4.179974	10.00659	1.56E-20	3.33E-19	35.83361	Up-Regulated
MEOX1	1.716388	5.483484	10.00544	1.58E-20	3.35E-19	35.82499	Up-Regulated
PRKAR2B	1.604946	7.581438	10.00041	1.64E-20	3.48E-19	35.7874	Up-Regulated
FAM13C	1.604497	6.276521	10.00009	1.64E-20	3.49E-19	35.78497	Up-Regulated
CH25H	1.697455	5.923846	9.977118	1.95E-20	4.12E-19	35.61322	Up-Regulated
VIPR2	2.465894	4.367773	9.97307	2.01E-20	4.25E-19	35.58297	Up-Regulated
PREX2	1.817334	5.631398	9.949183	2.41E-20	5.07E-19	35.4046	Up-Regulated
PRND	1.994514	4.110527	9.945066	2.49E-20	5.23E-19	35.37388	Up-Regulated
TNXB	2.096501	9.033	9.935376	2.68E-20	5.61E-19	35.30159	Up-Regulated
CCL21	2.688047	8.061773	9.933663	2.71E-20	5.67E-19	35.28882	Up-Regulated
NR2F1	1.612288	8.344622	9.929386	2.80E-20	5.85E-19	35.25693	Up-Regulated
ASB2	2.131576	6.858145	9.928032	2.83E-20	5.91E-19	35.24684	Up-Regulated
CD36	1.599161	7.699053	9.926259	2.87E-20	5.98E-19	35.23362	Up-Regulated
C1QTNF7	2.09106	5.354347	9.908255	3.29E-20	6.82E-19	35.09948	Up-Regulated
ALPK2	1.71898	5.39074	9.905967	3.34E-20	6.94E-19	35.08243	Up-Regulated
PTGDS	2.08093	8.733148	9.897313	3.57E-20	7.39E-19	35.018	Up-Regulated
C2orf40	3.287601	4.495305	9.888824	3.81E-20	7.84E-19	34.95483	Up-Regulated
KCNA5	1.716273	3.873716	9.879699	4.08E-20	8.39E-19	34.88696	Up-Regulated
LYVE1	1.631433	5.781309	9.868744	4.43E-20	9.07E-19	34.80551	Up-Regulated
ISLR2	1.79468	5.317747	9.864573	4.57E-20	9.34E-19	34.77452	Up-Regulated
CYS1	2.053091	5.990352	9.843928	5.34E-20	1.09E-18	34.62119	Up-Regulated
ARSI	1.752546	5.534427	9.843193	5.36E-20	1.09E-18	34.61573	Up-Regulated
CACNA1H	1.716439	9.3161	9.839844	5.50E-20	1.12E-18	34.59088	Up-Regulated
PPP2R2B	1.693281	4.079504	9.80945	6.91E-20	1.39E-18	34.36549	Up-Regulated
KCNA3	1.844771	5.520004	9.801952	7.31E-20	1.47E-18	34.30995	Up-Regulated
C6orf186	2.202821	3.322679	9.790105	7.99E-20	1.60E-18	34.22222	Up-Regulated
FXYD1	2.163364	4.791542	9.776113	8.88E-20	1.78E-18	34.11869	Up-Regulated
SCUBE2	2.073755	5.610962	9.770888	9.23E-20	1.85E-18	34.08005	Up-Regulated
ITIH5	1.706043	8.517557	9.767703	9.45E-20	1.89E-18	34.0565	Up-Regulated
HS3ST2	1.864521	4.766838	9.761817	9.88E-20	1.97E-18	34.01299	Up-Regulated
MMRN1	2.091317	6.30839	9.73952	1.17E-19	2.31E-18	33.84829	Up-Regulated
NRXN3	2.367874	6.480965	9.726557	1.29E-19	2.53E-18	33.75263	Up-Regulated
SLC22A17	1.58854	7.02981	9.722321	1.33E-19	2.61E-18	33.72138	Up-Regulated
EBF2	1.892293	3.144415	9.701102	1.56E-19	3.05E-18	33.56496	Up-Regulated
PLD4	2.14684	5.431127	9.694977	1.63E-19	3.19E-18	33.51984	Up-Regulated
NEGR1	1.91393	7.037681	9.689913	1.69E-19	3.30E-18	33.48255	Up-Regulated
HSPB8	2.023581	8.175727	9.68773	1.72E-19	3.35E-18	33.46648	Up-Regulated
LRRN2	1.852508	5.952741	9.67986	1.82E-19	3.55E-18	33.40855	Up-Regulated
SIGLEC6	1.997462	3.685029	9.67689	1.86E-19	3.63E-18	33.3867	Up-Regulated

FOXP2	2.552432	5.128552	9.6661	2.02E-19	3.92E-18	33.30734	Up-Regulated
RERG	1.861148	6.6962	9.662554	2.07E-19	4.02E-18	33.28126	Up-Regulated
FGF2	1.647602	6.782496	9.660336	2.11E-19	4.08E-18	33.26496	Up-Regulated
PLP1	2.652605	3.292443	9.642927	2.40E-19	4.63E-18	33.13704	Up-Regulated
CDH2	2.005728	5.228975	9.625525	2.73E-19	5.23E-18	33.0093	Up-Regulated
AOX1	2.352989	5.460581	9.591098	3.53E-19	6.71E-18	32.75694	Up-Regulated
BVES	1.779786	6.61774	9.578261	3.88E-19	7.35E-18	32.66296	Up-Regulated
FPR1	1.648619	6.527351	9.576321	3.93E-19	7.45E-18	32.64876	Up-Regulated
LMO3	2.258669	5.060434	9.525397	5.74E-19	1.07E-17	32.27666	Up-Regulated
FGF10	1.893551	2.320201	9.520217	5.96E-19	1.11E-17	32.23887	Up-Regulated
ATP1A2	2.906067	4.889569	9.486229	7.66E-19	1.41E-17	31.99118	Up-Regulated
BCHE	2.319521	4.025422	9.483787	7.80E-19	1.44E-17	31.97341	Up-Regulated
TMEM100	2.088786	4.726732	9.472605	8.47E-19	1.55E-17	31.89203	Up-Regulated
ACTG2	2.916066	11.16024	9.465467	8.93E-19	1.64E-17	31.84011	Up-Regulated
HLA-DQA1	1.829431	10.32014	9.465319	8.94E-19	1.64E-17	31.83903	Up-Regulated
SCN2B	1.932784	2.816536	9.447976	1.02E-18	1.85E-17	31.71298	Up-Regulated
TMEM35	2.245089	3.715314	9.440539	1.07E-18	1.95E-17	31.65896	Up-Regulated
AGTR1	2.439894	3.686197	9.436721	1.10E-18	2.01E-17	31.63123	Up-Regulated
TCEAL2	2.843786	3.667177	9.429258	1.17E-18	2.12E-17	31.57707	Up-Regulated
FAM65B	1.596598	6.80156	9.412364	1.32E-18	2.39E-17	31.45453	Up-Regulated
LOC401093	1.705157	6.537959	9.410336	1.34E-18	2.43E-17	31.43982	Up-Regulated
RGS9	1.802531	3.945774	9.408361	1.36E-18	2.46E-17	31.42551	Up-Regulated
PTPRD	1.787559	5.431835	9.403631	1.41E-18	2.54E-17	31.39123	Up-Regulated
ABCD2	1.654173	4.092009	9.390187	1.55E-18	2.80E-17	31.29385	Up-Regulated
COMP	3.082439	6.43389	9.374048	1.75E-18	3.14E-17	31.17704	Up-Regulated
CD52	1.596646	8.419012	9.327851	2.45E-18	4.34E-17	30.84331	Up-Regulated
WSCD2	2.65997	4.166503	9.326168	2.48E-18	4.39E-17	30.83116	Up-Regulated
CHRM2	2.845818	2.940218	9.313051	2.73E-18	4.83E-17	30.73658	Up-Regulated
PKNOX2	1.673338	4.488261	9.306582	2.87E-18	5.05E-17	30.68995	Up-Regulated
TWIST1	1.673879	5.612562	9.299785	3.01E-18	5.29E-17	30.64099	Up-Regulated
GDF10	2.053784	2.815449	9.292586	3.17E-18	5.56E-17	30.58915	Up-Regulated
FCRL6	1.692368	3.721456	9.274266	3.63E-18	6.34E-17	30.45733	Up-Regulated
NXPH3	2.190485	5.16263	9.271071	3.71E-18	6.48E-17	30.43436	Up-Regulated
MYOCD	2.221399	6.775201	9.269278	3.76E-18	6.56E-17	30.42147	Up-Regulated
CORIN	1.716395	5.312655	9.254752	4.18E-18	7.25E-17	30.31708	Up-Regulated
CRISPLD1	1.620477	6.78492	9.251109	4.29E-18	7.43E-17	30.29091	Up-Regulated
LY9	1.820688	5.527313	9.249451	4.35E-18	7.51E-17	30.279	Up-Regulated
PTPN5	1.805206	3.036525	9.246236	4.45E-18	7.68E-17	30.25592	Up-Regulated
PALM	1.613657	7.898722	9.242709	4.56E-18	7.86E-17	30.2306	Up-Regulated
NFASC	1.910999	8.015051	9.230144	5.00E-18	8.56E-17	30.14044	Up-Regulated
PCDH9	2.109622	4.066987	9.21367	5.64E-18	9.63E-17	30.02234	Up-Regulated
PYGM	2.055897	4.481019	9.204975	6.01E-18	1.02E-16	29.96005	Up-Regulated
KCND2	1.589594	4.02547	9.198895	6.28E-18	1.07E-16	29.91651	Up-Regulated

HPGDS	1.652871	4.158403	9.180803	7.16E-18	1.22E-16	29.78706	Up-Regulated
TM7SF4	1.607337	3.107512	9.15697	8.51E-18	1.44E-16	29.61675	Up-Regulated
HGF	1.591625	5.810019	9.14929	8.99E-18	1.52E-16	29.56192	Up-Regulated
ZNF831	1.66495	5.324884	9.139221	9.67E-18	1.63E-16	29.49007	Up-Regulated
AKAP12	1.671081	9.564899	9.13307	1.01E-17	1.70E-16	29.44621	Up-Regulated
ZBTB16	2.268888	4.906617	9.095526	1.33E-17	2.21E-16	29.17882	Up-Regulated
FAM198A	1.919172	5.199812	9.085056	1.43E-17	2.38E-16	29.10436	Up-Regulated
LAX1	1.638328	5.957479	9.079524	1.49E-17	2.47E-16	29.06503	Up-Regulated
GZMK	1.95629	5.761208	9.070148	1.59E-17	2.64E-16	28.99843	Up-Regulated
CSMD2	1.704573	5.003874	9.056951	1.75E-17	2.90E-16	28.90473	Up-Regulated
CCL11	1.816226	7.151378	9.055971	1.76E-17	2.92E-16	28.89778	Up-Regulated
NLGN4X	1.702876	5.66059	9.042144	1.95E-17	3.22E-16	28.7997	Up-Regulated
NAP1L2	1.851689	3.534197	9.025838	2.19E-17	3.60E-16	28.68415	Up-Regulated
PDZRN3	1.586626	8.617326	9.012859	2.40E-17	3.95E-16	28.59226	Up-Regulated
NECAB1	1.669469	5.089182	8.986568	2.90E-17	4.73E-16	28.40635	Up-Regulated
BAI3	1.824871	2.50041	8.971379	3.24E-17	5.26E-16	28.29909	Up-Regulated
PRUNE2	2.466922	8.918318	8.965028	3.39E-17	5.50E-16	28.25427	Up-Regulated
P2RY10	1.689421	5.580183	8.962549	3.45E-17	5.59E-16	28.23678	Up-Regulated
CRTAC1	2.281118	2.929533	8.961317	3.48E-17	5.64E-16	28.22809	Up-Regulated
SORCS1	2.098494	2.970223	8.940961	4.02E-17	6.47E-16	28.0846	Up-Regulated
CD79B	1.844924	5.721167	8.934654	4.21E-17	6.76E-16	28.04018	Up-Regulated
ACSS3	1.817379	4.88491	8.924769	4.52E-17	7.22E-16	27.9706	Up-Regulated
SCN4B	1.609087	5.663037	8.90683	5.14E-17	8.19E-16	27.84443	Up-Regulated
FLNC	2.524241	10.11459	8.904404	5.22E-17	8.31E-16	27.82739	Up-Regulated
PSD	2.175927	5.951828	8.894824	5.59E-17	8.88E-16	27.76008	Up-Regulated
CACNA1C	1.692031	8.221083	8.88082	6.18E-17	9.79E-16	27.66177	Up-Regulated
PLA2G2D	2.366707	5.599385	8.869813	6.68E-17	1.05E-15	27.58456	Up-Regulated
FAM159A	1.669331	3.576437	8.839142	8.31E-17	1.30E-15	27.36971	Up-Regulated
SAMD11	1.622486	5.985989	8.837649	8.40E-17	1.31E-15	27.35926	Up-Regulated
CCL13	1.754431	4.323625	8.83381	8.63E-17	1.35E-15	27.33241	Up-Regulated
KLF17	1.647012	2.478345	8.790001	1.18E-16	1.82E-15	27.0264	Up-Regulated
GRIK5	2.113733	4.726108	8.784533	1.22E-16	1.89E-15	26.98827	Up-Regulated
XCR1	1.601491	2.680698	8.780109	1.26E-16	1.95E-15	26.95743	Up-Regulated
SYNM	2.486351	9.654864	8.770749	1.35E-16	2.07E-15	26.8922	Up-Regulated
LRRC15	2.017154	7.42219	8.765719	1.40E-16	2.15E-15	26.85717	Up-Regulated
CD38	1.742649	6.37954	8.75431	1.52E-16	2.32E-15	26.77775	Up-Regulated
CCR7	1.747526	6.459277	8.740256	1.67E-16	2.56E-15	26.68001	Up-Regulated
GLRB	1.59687	4.590255	8.727162	1.84E-16	2.80E-15	26.58903	Up-Regulated
CD27	1.658742	7.046544	8.721533	1.91E-16	2.91E-15	26.54994	Up-Regulated
ASPA	1.641371	3.151675	8.720064	1.93E-16	2.93E-15	26.53974	Up-Regulated
MKX	1.954782	4.757091	8.718003	1.96E-16	2.97E-15	26.52544	Up-Regulated
CTSG	2.052917	2.767163	8.699081	2.24E-16	3.39E-15	26.39418	Up-Regulated
WISP2	2.010615	3.619865	8.649776	3.16E-16	4.74E-15	26.05297	Up-Regulated

SOX10	2.155822	3.770272	8.638804	3.42E-16	5.11E-15	25.9772	Up-Regulated
NGFR	2.480867	5.912486	8.609685	4.19E-16	6.23E-15	25.77639	Up-Regulated
HAMP	1.645348	2.980023	8.608198	4.23E-16	6.29E-15	25.76614	Up-Regulated
PPAPDC1A	2.032857	3.798843	8.605064	4.33E-16	6.43E-15	25.74455	Up-Regulated
GLP2R	1.927311	2.220643	8.571418	5.47E-16	8.07E-15	25.5131	Up-Regulated
TACR1	1.773941	3.665221	8.57086	5.50E-16	8.10E-15	25.50927	Up-Regulated
BEND5	1.658939	3.855375	8.551493	6.29E-16	9.22E-15	25.3763	Up-Regulated
CD1C	1.814176	4.552399	8.550674	6.33E-16	9.26E-15	25.37068	Up-Regulated
SGCA	2.168953	4.863399	8.485239	9.97E-16	1.44E-14	24.92279	Up-Regulated
CACNA2D1	1.973413	6.100829	8.484674	1.00E-15	1.45E-14	24.91893	Up-Regulated
SORBS1	1.689969	10.5027	8.472833	1.09E-15	1.56E-14	24.8381	Up-Regulated
SMAD9	1.789901	5.908148	8.471041	1.10E-15	1.58E-14	24.82587	Up-Regulated
NOG	1.593005	2.886247	8.468541	1.12E-15	1.61E-14	24.80882	Up-Regulated
C1orf95	2.065357	3.696568	8.463753	1.16E-15	1.66E-14	24.77617	Up-Regulated
DES	3.596241	10.43101	8.452183	1.25E-15	1.79E-14	24.69732	Up-Regulated
NCAM1	1.805489	6.332271	8.445485	1.31E-15	1.87E-14	24.6517	Up-Regulated
ADH1B	2.800502	6.265086	8.442485	1.34E-15	1.91E-14	24.63128	Up-Regulated
LOC100126784	1.595348	4.335352	8.41141	1.66E-15	2.34E-14	24.41996	Up-Regulated
PAR5	1.670526	3.645271	8.398588	1.82E-15	2.55E-14	24.33292	Up-Regulated
ADCY5	2.389556	6.624406	8.390734	1.92E-15	2.68E-14	24.27963	Up-Regulated
GPM6A	2.008727	2.997186	8.37226	2.18E-15	3.02E-14	24.15443	Up-Regulated
SUCNR1	1.767651	4.293796	8.370967	2.20E-15	3.05E-14	24.14567	Up-Regulated
AFF3	1.850693	5.598974	8.342541	2.67E-15	3.68E-14	23.95338	Up-Regulated
IPW	1.626989	6.51617	8.337482	2.76E-15	3.81E-14	23.91919	Up-Regulated
TUB	1.693234	5.469894	8.328051	2.95E-15	4.06E-14	23.8555	Up-Regulated
PAK3	1.732962	3.165759	8.272761	4.31E-15	5.84E-14	23.48304	Up-Regulated
GREM2	2.421648	5.874634	8.267114	4.48E-15	6.06E-14	23.44509	Up-Regulated
VIP	2.444935	3.097649	8.260251	4.69E-15	6.33E-14	23.39899	Up-Regulated
PCDH7	1.68315	9.399009	8.245355	5.19E-15	6.98E-14	23.299	Up-Regulated
RELN	2.021733	4.075855	8.242142	5.31E-15	7.12E-14	23.27745	Up-Regulated
CDH23	1.637482	4.997091	8.237115	5.49E-15	7.35E-14	23.24374	Up-Regulated
MYOC	2.635718	2.963123	8.221137	6.12E-15	8.15E-14	23.13669	Up-Regulated
THSD7B	2.040016	3.202994	8.189597	7.59E-15	1.00E-13	22.92575	Up-Regulated
CST2	2.009991	4.753498	8.136819	1.09E-14	1.42E-13	22.57393	Up-Regulated
CRTAM	1.634601	3.83159	8.127655	1.15E-14	1.51E-13	22.51299	Up-Regulated
LDB3	2.136281	5.281136	8.127201	1.16E-14	1.51E-13	22.50996	Up-Regulated
ABCA8	2.455885	5.737362	8.113731	1.27E-14	1.65E-13	22.42048	Up-Regulated
REEP2	1.710654	5.316538	8.083129	1.56E-14	2.02E-13	22.21751	Up-Regulated
MS4A2	1.661513	5.027691	8.045285	2.01E-14	2.58E-13	21.9672	Up-Regulated
NPR3	1.783865	3.230698	8.031389	2.21E-14	2.82E-13	21.87547	Up-Regulated
C5orf23	1.807357	4.458535	8.027149	2.27E-14	2.90E-13	21.8475	Up-Regulated
FCRLA	2.198465	4.679397	8.004464	2.64E-14	3.36E-13	21.69803	Up-Regulated

MAMDC2	2.197639	5.084205	7.967977	3.38E-14	4.25E-13	21.45821	Up-Regulated
TMEM59L	1.752326	3.045112	7.949922	3.81E-14	4.77E-13	21.33979	Up-Regulated
ADCYAP1	1.836638	3.40955	7.944961	3.94E-14	4.93E-13	21.30728	Up-Regulated
NTN1	1.652882	8.417929	7.944074	3.96E-14	4.95E-13	21.30148	Up-Regulated
ADAMTS8	1.982118	5.56461	7.925589	4.48E-14	5.59E-13	21.18048	Up-Regulated
ASB5	2.54301	2.496349	7.920317	4.64E-14	5.77E-13	21.146	Up-Regulated
NPTXR	1.722045	7.233463	7.859638	6.94E-14	8.48E-13	20.75029	Up-Regulated
CD22	1.987943	6.092609	7.845859	7.60E-14	9.25E-13	20.66072	Up-Regulated
ANGPTL7	1.630024	2.59302	7.822244	8.88E-14	1.08E-12	20.50743	Up-Regulated
PLIN4	2.314707	6.637866	7.817433	9.17E-14	1.11E-12	20.47624	Up-Regulated
ZNF385B	1.777286	4.14741	7.806827	9.83E-14	1.19E-12	20.40752	Up-Regulated
KIAA2022	1.826647	3.468167	7.786732	1.12E-13	1.35E-12	20.2775	Up-Regulated
KCNA1	2.067166	2.119005	7.781085	1.17E-13	1.39E-12	20.241	Up-Regulated
C20orf200	1.762563	2.51287	7.742963	1.50E-13	1.77E-12	19.99505	Up-Regulated
TNN	1.879511	4.373378	7.740125	1.52E-13	1.80E-12	19.97678	Up-Regulated
SLITRK5	2.062851	3.189114	7.729632	1.63E-13	1.93E-12	19.90924	Up-Regulated
STMN2	1.912713	3.877483	7.727447	1.66E-13	1.95E-12	19.89519	Up-Regulated
RBPMS2	1.699844	7.009482	7.722232	1.71E-13	2.01E-12	19.86165	Up-Regulated
TACR2	2.362525	5.885262	7.717521	1.77E-13	2.08E-12	19.83137	Up-Regulated
CPA3	1.751153	7.431851	7.697162	2.02E-13	2.37E-12	19.70064	Up-Regulated
DLG2	1.732256	4.178735	7.683475	2.21E-13	2.58E-12	19.61288	Up-Regulated
PNOC	1.619011	3.535889	7.681536	2.24E-13	2.61E-12	19.60046	Up-Regulated
COL4A4	1.769484	6.441067	7.681534	2.24E-13	2.61E-12	19.60045	Up-Regulated
GFRA1	2.221447	6.719053	7.675854	2.32E-13	2.70E-12	19.56406	Up-Regulated
SELE	1.588375	6.447019	7.642786	2.88E-13	3.33E-12	19.35262	Up-Regulated
EPHA7	2.157282	5.253796	7.609519	3.57E-13	4.09E-12	19.14052	Up-Regulated
C9orf4	1.782073	1.812235	7.60905	3.58E-13	4.10E-12	19.13753	Up-Regulated
COL11A1	2.59224	6.913333	7.594057	3.95E-13	4.50E-12	19.04215	Up-Regulated
FCER1A	1.855558	3.607926	7.582005	4.27E-13	4.85E-12	18.96557	Up-Regulated
GAP43	1.631879	3.101641	7.57566	4.45E-13	5.04E-12	18.92529	Up-Regulated
WNT2	1.712392	6.328643	7.565191	4.76E-13	5.38E-12	18.85887	Up-Regulated
KCNA6	1.661519	3.278673	7.557351	5.01E-13	5.65E-12	18.80917	Up-Regulated
PRG4	1.596784	4.714378	7.533362	5.85E-13	6.56E-12	18.65733	Up-Regulated
NOVA1	1.754065	4.771891	7.514493	6.60E-13	7.36E-12	18.53812	Up-Regulated
KIAA0408	2.481738	2.923448	7.461588	9.27E-13	1.02E-11	18.20497	Up-Regulated
HAS1	1.656305	3.136067	7.453709	9.75E-13	1.07E-11	18.1555	Up-Regulated
MGC45800	1.597835	2.526054	7.428039	1.15E-12	1.26E-11	17.99455	Up-Regulated
CDH19	1.996398	2.847102	7.426685	1.16E-12	1.27E-11	17.98607	Up-Regulated
NRXN1	1.942309	3.182961	7.41183	1.27E-12	1.39E-11	17.89312	Up-Regulated
KCNB1	1.918902	2.859611	7.408102	1.30E-12	1.42E-11	17.86981	Up-Regulated
GRIK3	1.865912	3.352821	7.401465	1.36E-12	1.48E-11	17.82834	Up-Regulated
LOC572558	1.60244	1.664457	7.383014	1.53E-12	1.65E-11	17.71317	Up-Regulated
STAP1	1.598952	3.531658	7.371708	1.64E-12	1.77E-11	17.6427	Up-Regulated

TLR10	1.690155	4.685771	7.366869	1.70E-12	1.82E-11	17.61256	Up-Regulated
SNORD116-4	1.967598	3.001351	7.334128	2.09E-12	2.22E-11	17.40901	Up-Regulated
SPEG	1.989577	7.350372	7.326099	2.20E-12	2.33E-11	17.35919	Up-Regulated
BMPER	1.586888	3.808586	7.318658	2.30E-12	2.44E-11	17.31305	Up-Regulated
CD79A	1.967947	8.009084	7.305169	2.51E-12	2.65E-11	17.22949	Up-Regulated
CNR1	2.066595	4.98725	7.304961	2.51E-12	2.65E-11	17.2282	Up-Regulated
EPYC	1.882231	2.030482	7.289653	2.76E-12	2.92E-11	17.13351	Up-Regulated
GALNTL1	1.755854	5.064974	7.284749	2.85E-12	3.00E-11	17.1032	Up-Regulated
MUSK	1.654427	2.487654	7.221695	4.24E-12	4.40E-11	16.71481	Up-Regulated
APOD	2.174418	9.051889	7.200678	4.83E-12	5.01E-11	16.58587	Up-Regulated
TPSB2	1.672637	8.020813	7.145527	6.82E-12	6.96E-11	16.24881	Up-Regulated
NLGN1	1.704091	3.384947	7.114686	8.26E-12	8.37E-11	16.06111	Up-Regulated
STON1-GTF2A1L	1.914516	2.046158	7.100393	9.03E-12	9.11E-11	15.97432	Up-Regulated
DTNA	1.693762	6.0981	7.085793	9.88E-12	9.93E-11	15.88579	Up-Regulated
CXCL13	1.935286	7.981161	7.077931	1.04E-11	1.04E-10	15.83818	Up-Regulated
RSPO2	1.840321	3.614884	7.049841	1.23E-11	1.23E-10	15.66836	Up-Regulated
PENK	1.989685	2.796594	7.049361	1.24E-11	1.23E-10	15.66546	Up-Regulated
CHI3L2	1.68363	4.992363	6.92648	2.63E-11	2.53E-10	14.92831	Up-Regulated
SIGLEC14	1.59834	4.754117	6.890287	3.27E-11	3.12E-10	14.71297	Up-Regulated
NBEA	1.739884	6.42822	6.888239	3.32E-11	3.15E-10	14.70081	Up-Regulated
MS4A1	2.328984	6.199884	6.884963	3.38E-11	3.21E-10	14.68137	Up-Regulated
NPTX1	2.107693	4.42762	6.884677	3.39E-11	3.21E-10	14.67967	Up-Regulated
FAM19A4	1.72035	1.67072	6.883675	3.41E-11	3.23E-10	14.67373	Up-Regulated
MARCO	1.919999	4.009106	6.879818	3.49E-11	3.31E-10	14.65084	Up-Regulated
UCHL1	1.613207	6.427404	6.842447	4.37E-11	4.10E-10	14.4296	Up-Regulated
BLK	1.964264	4.428244	6.835902	4.55E-11	4.26E-10	14.39094	Up-Regulated
AHNAK2	1.641142	10.2087	6.812491	5.24E-11	4.86E-10	14.25289	Up-Regulated
PRIMA1	2.020268	5.489786	6.804701	5.49E-11	5.08E-10	14.20704	Up-Regulated
FCRL5	1.642523	6.215279	6.788323	6.06E-11	5.59E-10	14.11074	Up-Regulated
CTNNA3	1.650503	2.348029	6.761646	7.11E-11	6.53E-10	13.95427	Up-Regulated
CXCR5	1.6555	5.03792	6.760999	7.14E-11	6.55E-10	13.95048	Up-Regulated
RYR2	1.785071	5.688868	6.688946	1.10E-10	9.86E-10	13.53014	Up-Regulated
FCRL3	1.651236	4.638188	6.672059	1.21E-10	1.09E-09	13.4321	Up-Regulated
CARTPT	1.916076	1.928613	6.666231	1.26E-10	1.12E-09	13.39832	Up-Regulated
KCNK3	1.673933	5.370575	6.637411	1.49E-10	1.32E-09	13.23154	Up-Regulated
ACTC1	1.750193	5.531192	6.625698	1.60E-10	1.41E-09	13.16391	Up-Regulated
ACTN2	1.856925	2.551107	6.622057	1.63E-10	1.44E-09	13.14291	Up-Regulated
KIAA0125	1.666807	4.611575	6.619529	1.66E-10	1.46E-09	13.12833	Up-Regulated
SIX2	1.949344	5.954974	6.549417	2.50E-10	2.16E-09	12.72567	Up-Regulated
SPIB	1.837122	5.645234	6.527074	2.85E-10	2.45E-09	12.59803	Up-Regulated
ITGA8	1.647561	6.143091	6.508929	3.17E-10	2.71E-09	12.4946	Up-Regulated
CHRNA3	1.722397	4.545434	6.506012	3.22E-10	2.75E-09	12.478	Up-Regulated

GRIN2A	1.785021	4.921194	6.468798	4.00E-10	3.39E-09	12.26664	Up-Regulated
HLA-DQA2	1.841454	6.751286	6.457794	4.27E-10	3.60E-09	12.20432	Up-Regulated
SMYD1	2.422374	2.832422	6.443898	4.62E-10	3.88E-09	12.12573	Up-Regulated
CLEC17A	1.590263	2.800226	6.425111	5.16E-10	4.29E-09	12.01968	Up-Regulated
FMN2	1.60765	3.108687	6.36099	7.46E-10	6.06E-09	11.65952	Up-Regulated
FCRL1	1.776618	3.28528	6.275288	1.22E-09	9.64E-09	11.18244	Up-Regulated
ECEL1	1.688494	4.367478	6.226866	1.60E-09	1.25E-08	10.91508	Up-Regulated
FAM129C	1.608315	3.118402	6.21133	1.75E-09	1.36E-08	10.82964	Up-Regulated
FCER2	1.622923	3.299618	6.081841	3.61E-09	2.70E-08	10.12393	Up-Regulated
DPP6	1.706675	2.579123	6.055374	4.18E-09	3.10E-08	9.981109	Up-Regulated
CES1	1.794106	6.5136	6.033482	4.71E-09	3.47E-08	9.86334	Up-Regulated
CD19	1.630373	4.463058	5.96371	6.92E-09	4.99E-08	9.490247	Up-Regulated
SCARA5	1.69011	5.094016	5.922405	8.67E-09	6.18E-08	9.270986	Up-Regulated
SNAP25	1.611571	4.132634	5.827221	1.45E-08	1.01E-07	8.770304	Up-Regulated
TCL1A	1.757584	3.363343	5.773852	1.93E-08	1.32E-07	8.492405	Up-Regulated
ADIPOQ	1.869887	1.927438	5.730314	2.44E-08	1.64E-07	8.267207	Up-Regulated
C16orf89	1.657303	4.560049	5.652258	3.68E-08	2.41E-07	7.866901	Up-Regulated
CHIT1	1.867771	4.924589	5.632747	4.08E-08	2.66E-07	7.767531	Up-Regulated
BMP3	1.835685	4.008657	5.463367	9.82E-08	6.07E-07	6.916612	Up-Regulated
FREM1	1.645604	4.987694	5.368679	1.59E-07	9.53E-07	6.45019	Up-Regulated
MAPK4	1.636252	4.034673	5.184661	3.98E-07	2.23E-06	5.563049	Up-Regulated
CR2	1.917283	6.203258	5.128349	5.25E-07	2.90E-06	5.296726	Up-Regulated
PPP1R1A	1.623999	3.71537	5.075168	6.80E-07	3.68E-06	5.047452	Up-Regulated
C4orf7	1.586216	5.063317	4.027191	7.15E-05	0.000269	0.595996	Up-Regulated
DUOXA2	-1.94851	6.749088	-4.73083	3.45E-06	1.66E-05	3.486901	Down-Regulated
S100A7	-1.90365	2.78254	-4.38218	1.63E-05	6.94E-05	2.003395	Down-Regulated
DUOX2	-1.76081	9.310458	-4.07365	5.93E-05	0.000228	0.774177	Down-Regulated
NOTUM	-1.60182	5.683168	-3.96919	9.03E-05	0.000334	0.376156	Down-Regulated
REG3A	-1.99659	6.546055	-3.56856	0.000418	0.001342	-1.06333	Down-Regulated

Supplementary Table 2.

DEGs associated with poor overall survival in TCGA database

Sig gene name	p. value	HR	Low 95%CI	High 95%CI
MMP16	5.72E-05	1.295856	1.142185	1.470201621
NPTX1	0.000214106	1.120607	1.055041	1.190247724
PRICKLE1	0.000269209	1.251101	1.109052	1.411343048
RGS4	0.000380853	1.203755	1.086702	1.333416069
NALCN	0.000406277	1.175169	1.074593	1.285156885
CNTN1	0.000507297	1.115196	1.048718	1.185888658
C5orf23	0.000545836	1.172825	1.071478	1.283758146
DCLK1	0.000589779	1.163153	1.067085	1.267868693
GDF6	0.00059745	1.180121	1.073642	1.297159501
AKAP12	0.000613739	1.194987	1.07919	1.323208947

BCHE	0.000674876	1.121784	1.049869	1.198625427
NPR3	0.00069059	1.164525	1.066452	1.271617553
CDH2	0.000708345	1.17305	1.06954	1.286577171
CPE	0.000727759	1.197707	1.078705	1.329837334
TNN	0.000733388	1.139665	1.056385	1.229509497
PTPRD	0.000748723	1.190466	1.075709	1.317465133
TMTC1	0.000795277	1.207049	1.081369	1.347336689
KCND2	0.000865481	1.216655	1.084062	1.365465899
GHR	0.000892351	1.184961	1.072074	1.309734875
ITIH3	0.000893218	1.168792	1.066043	1.281445387
GLP2R	0.000949911	1.144506	1.056466	1.239883911
PRKD1	0.001035552	1.221996	1.084057	1.377487726
DPP6	0.001054863	1.109306	1.042542	1.180344456
VCAN	0.001117471	1.266691	1.098822	1.46020612
LPPR4	0.001137881	1.207968	1.078032	1.353565213
TACR1	0.001188101	1.164676	1.062129	1.277124802
LRRC32	0.001203879	1.289166	1.105447	1.503417536
EBF2	0.001236916	1.168462	1.063136	1.284221679
FAT3	0.001281353	1.135909	1.051134	1.227521326
CHRD	0.001341804	1.211251	1.077367	1.361772023
APOD	0.001354009	1.12126	1.045448	1.202569404
FBN1	0.001520438	1.245018	1.087286	1.425631227
NPAS3	0.001571064	1.182827	1.065884	1.31259967
THSD7B	0.001578858	1.130663	1.047735	1.220154396
THSD7A	0.001578944	1.227305	1.080877	1.393569106
PDE1B	0.001594523	1.208365	1.074399	1.359035027
CPZ	0.00163095	1.210867	1.074974	1.363937871
FBXL7	0.001702813	1.257392	1.089774	1.450791985
KCNB1	0.001716952	1.116019	1.042007	1.195288559
CD36	0.001766698	1.194408	1.068545	1.33509782
CDO1	0.001805745	1.146729	1.052236	1.249707643
GALNTL1	0.001843062	1.139156	1.04948	1.236494679
SVEP1	0.001872785	1.170226	1.059842	1.292107149
GALNTL2	0.001909892	1.171422	1.060045	1.294501106
GPR133	0.001928644	1.142183	1.050133	1.242301824
RECK	0.002037362	1.253989	1.086026	1.447927876
RBMS3	0.002063393	1.195546	1.067142	1.339399936
KLHL4	0.002077549	1.153426	1.053243	1.263137956
MXRA8	0.002092522	1.215105	1.073281	1.375670663
KCNT2	0.002150316	1.184273	1.063011	1.319368274
MN1	0.002200617	1.167828	1.057417	1.289767919
RASSF8	0.002240393	1.195881	1.066274	1.341242639
EFEMP1	0.002243992	1.178235	1.060585	1.30893532

MMRN1	0.002437234	1.153349	1.051707	1.264813286
KCNA1	0.002451244	1.098544	1.033733	1.167417506
PDGFRL	0.002496519	1.162584	1.054428	1.281832656
SLIT2	0.002537954	1.117714	1.039804	1.201461527
SLC22A17	0.002547128	1.189311	1.062653	1.331064047
PODN	0.002604479	1.17152	1.056811	1.298681036
KCNJ8	0.002628353	1.256897	1.082932	1.458809534
LTBP2	0.002798343	1.237362	1.076082	1.422813602
CHRDL1	0.002833378	1.091765	1.030613	1.156546021
PDE3A	0.003078365	1.153728	1.049492	1.268318017
MAP6	0.003117197	1.1661	1.05314	1.291176004
NUDT10	0.003146323	1.163254	1.052171	1.286066232
RNF180	0.003174266	1.19899	1.062813	1.352615738
ACSS3	0.003174414	1.159461	1.050919	1.279212891
ECM2	0.003195754	1.249513	1.077528	1.448947625
FLRT2	0.003262518	1.1575	1.050029	1.275971663
LRCH2	0.003293046	1.200429	1.062742	1.355954767
C20orf103	0.003320262	1.150642	1.047767	1.263617702
ABCA6	0.00339314	1.150312	1.047441	1.263285472
PDE2A	0.003465631	1.185911	1.05779	1.329549423
TIMP2	0.003478022	1.279258	1.084463	1.509043835
MAPK4	0.003491701	1.096793	1.030859	1.166944174
ABCA9	0.003546244	1.134559	1.042257	1.235035886
ADH1B	0.003580289	1.091214	1.028969	1.15722316
RNF150	0.003610875	1.13051	1.040869	1.22786955
GGT5	0.00364385	1.238916	1.072311	1.431406088
SORCS2	0.00365659	1.165988	1.051278	1.293214481
CST2	0.003664887	1.131175	1.040931	1.229242367
CSDC2	0.00369504	1.159441	1.049234	1.281222627
PDE1A	0.003699421	1.19777	1.060362	1.352983233
ZFHX4	0.003741124	1.140428	1.043489	1.246371769
FGFR1	0.003786183	1.195281	1.059326	1.348684846
AKT3	0.003840362	1.21643	1.065128	1.389224389
ABCA8	0.0038861	1.102408	1.031808	1.177837955
VGLL3	0.00390527	1.189667	1.057296	1.338609332
NUDT11	0.003906245	1.17546	1.053226	1.31187893
KAL1	0.003945349	1.208896	1.062596	1.375338558
AR	0.004052374	1.134497	1.040951	1.23644992
SV2B	0.004074909	1.156918	1.047387	1.277904227
FREM1	0.00422701	1.101983	1.031049	1.177797147
AEBP1	0.004252213	1.206432	1.060781	1.372080825
GREM1	0.004353439	1.130111	1.038979	1.229237275
FERMT2	0.004372968	1.183131	1.053917	1.328187904

A2M	0.004435747	1.245082	1.070591	1.448012029
CASQ2	0.004463885	1.094594	1.028479	1.16495961
COL8A2	0.004505867	1.194069	1.056517	1.349529118
ADIPOQ	0.004576797	1.08163	1.024523	1.141918948
TCEAL7	0.004653998	1.187601	1.054283	1.337777085
ELN	0.004655663	1.161429	1.04708	1.288265938
BGN	0.004716901	1.216926	1.062	1.394453913
NOVA1	0.004756937	1.125445	1.036791	1.221680297
PDZD4	0.00476014	1.12716	1.037267	1.224842952
ACSM5	0.004774171	1.180408	1.051966	1.324533228
NRK	0.004777753	1.10541	1.031078	1.185101555
LAMA2	0.004976703	1.168068	1.048057	1.301821218
BOC	0.005090963	1.137129	1.039347	1.244110372
OLFML2B	0.005147822	1.208842	1.058433	1.380625974
C7	0.005155987	1.080059	1.023318	1.139946582
ASPN	0.005209311	1.152995	1.043405	1.274095622
RERG	0.005312164	1.14032	1.039751	1.250615781
MSRB3	0.005324435	1.147176	1.041576	1.263482627
CRTAC1	0.005423248	1.110722	1.031481	1.196050088
PDGFRB	0.005573136	1.263577	1.070929	1.490880868
SCUBE2	0.005769287	1.131452	1.036468	1.235141235
PTPN5	0.005831736	1.143374	1.039498	1.257631903
CTHRC1	0.005922294	1.189306	1.051165	1.345601188
PALM	0.005968568	1.164823	1.044785	1.298652215
ZBTB16	0.005993007	1.105427	1.029165	1.187341058
NAV3	0.006050205	1.151586	1.041192	1.273684855
PTGER3	0.006083667	1.136368	1.037176	1.245047561
EDNRA	0.006137881	1.219449	1.058124	1.405369369
FABP4	0.006192899	1.10037	1.027536	1.178367257
ABCC9	0.006254539	1.147837	1.039813	1.267084905
ISLR2	0.006307724	1.164428	1.04393	1.298835375
PABPC5	0.006318631	1.197331	1.052149	1.362546674
NTM	0.006402466	1.173594	1.046027	1.316717294
ZFPM2	0.006417115	1.153484	1.040928	1.278212058
FNDC1	0.006419142	1.119883	1.032315	1.214878989
ZNF521	0.006512619	1.223859	1.058115	1.415565598
DAAM2	0.006562977	1.169714	1.0447	1.309689002
ADD2	0.006634695	1.12633	1.033626	1.227349333
SGCD	0.006643313	1.153669	1.040523	1.27911832
TUB	0.006659293	1.141363	1.037398	1.255746837
FBLN5	0.006702267	1.202481	1.052423	1.373933626
HTRA3	0.006766515	1.204949	1.052856	1.379014009
SLC24A3	0.00681857	1.179452	1.046536	1.32925073

KCNE4	0.006844623	1.180337	1.046693	1.33104436
LHFP	0.006957964	1.218828	1.055672	1.407199937
PLXDC2	0.006987611	1.213593	1.054351	1.396885183
GXYLT2	0.007085811	1.150508	1.038893	1.274114733
ADAM33	0.007132959	1.110271	1.028812	1.198179629
ISLR	0.007253919	1.159085	1.040668	1.290975448
COL8A1	0.00727319	1.139343	1.03582	1.253212264
DPYSL3	0.007276128	1.1424	1.036564	1.259042542
CDH11	0.007327321	1.213759	1.053514	1.398378347
LOC399959	0.007368846	1.12941	1.033226	1.234547972
OMD	0.007429703	1.106133	1.027385	1.190917743
SSC5D	0.007482553	1.133261	1.033989	1.242062453
ITGA11	0.007582883	1.180158	1.04504	1.332745807
ANTXR1	0.007600283	1.205553	1.050933	1.382922746
TIMP3	0.007618226	1.19194	1.047726	1.356004224
NOX4	0.007703645	1.203652	1.050249	1.37946135
NR2F1	0.007712559	1.176853	1.043994	1.326618692
DCLK2	0.007798071	1.161853	1.0403	1.297609917
PREX2	0.007835417	1.158687	1.039484	1.2915593
NAP1L3	0.007850262	1.160488	1.039881	1.295083592
C1QTNF7	0.007877727	1.128163	1.032151	1.233106643
NGFR	0.007912279	1.093901	1.023793	1.168808464
HSPB2	0.008041612	1.176671	1.043288	1.327106805
MOXD1	0.008074418	1.164376	1.040377	1.303154437
RSPO1	0.008184746	1.122772	1.030424	1.223395367
C21orf34	0.008195286	1.129986	1.032118	1.23713296
SCARF2	0.00824107	1.194179	1.046878	1.362205606
CNTN4	0.008242303	1.177614	1.043108	1.329465613
ARHGAP20	0.008246238	1.166194	1.04048	1.307098571
NPR1	0.008257815	1.193909	1.046781	1.36171581
HSPB7	0.008261778	1.086252	1.021568	1.155032052
POSTN	0.008288999	1.196563	1.047314	1.367081822
TCEAL2	0.008293417	1.082812	1.020703	1.148700552
PCDHGB7	0.00830329	1.188454	1.045451	1.351017136
NTRK3	0.008309547	1.129027	1.031726	1.235503028
ADCYAP1	0.008339552	1.123512	1.030386	1.22505511
TMEM59L	0.008377695	1.118812	1.029226	1.216196554
MAMDC2	0.008468617	1.096204	1.023751	1.173784782
THBS1	0.008609496	1.181568	1.043283	1.338181543
MPDZ	0.008685656	1.165923	1.039621	1.307568309
DZIP1	0.008805573	1.176474	1.041769	1.328597103
PLN	0.008825006	1.094532	1.022983	1.17108467
PDLIM3	0.008838755	1.126562	1.030415	1.231680975

NNMT	0.00884086	1.192924	1.045346	1.361337963
FAM19A5	0.008911003	1.177794	1.041865	1.331457917
HMCN1	0.00891123	1.13628	1.032537	1.2504457
CLIP3	0.008918982	1.159328	1.037734	1.295170867
ASPA	0.008962751	1.14645	1.034766	1.270189573
SSPN	0.009147218	1.173359	1.040452	1.323242103
VIPR2	0.009166345	1.105911	1.025264	1.192901759
ACTA2	0.009207308	1.141386	1.033258	1.260829161
PRELP	0.009297454	1.100271	1.023828	1.182421077
GPR124	0.009363196	1.236727	1.053597	1.451687406
COL14A1	0.00943969	1.134341	1.031354	1.247611361
FAM26E	0.009440968	1.19328	1.044226	1.363608619
SCN4B	0.009542692	1.146786	1.03396	1.271923655
MYL9	0.009660356	1.118076	1.027446	1.216699259
NAP1L2	0.009667184	1.121873	1.028283	1.223981457
COLEC12	0.009813406	1.140087	1.032102	1.25937052
ADAMTS12	0.009907612	1.156866	1.035596	1.292337387
PKNOX2	0.009952628	1.141868	1.032294	1.26307287
CCDC8	0.00997193	1.143245	1.032565	1.265788083
GRIK3	0.010088453	1.10119	1.023225	1.185096069
DACT1	0.010102423	1.177026	1.039563	1.332666682
CARTPT	0.010196437	1.081843	1.018827	1.148757205
C14orf132	0.010269167	1.141394	1.031753	1.262685661
TAGLN	0.010383292	1.11637	1.026232	1.214425356
FSTL1	0.010391805	1.229504	1.049782	1.439993808
ROR2	0.010426697	1.136902	1.030584	1.254187552
FXYD6	0.010448965	1.160708	1.035576	1.300959287
BEND5	0.010564243	1.136819	1.030386	1.254245379
FOXP2	0.010600941	1.093417	1.021033	1.17093253
SETBP1	0.010641476	1.151082	1.033279	1.282315797
SERPINF1	0.010686739	1.173927	1.03794	1.327730303
NRP2	0.010708977	1.189228	1.041027	1.358526217
FLNC	0.010741	1.084552	1.018984	1.154340256
NXPH3	0.010758398	1.104232	1.023221	1.191657758
ITGA9	0.010783889	1.145583	1.031932	1.271750255
GLT8D2	0.011025751	1.201778	1.042976	1.384759263
GUCY1B3	0.011099649	1.199908	1.04247	1.381124451
FLNA	0.011156839	1.138414	1.029955	1.258294772
DTNA	0.011216324	1.107442	1.023448	1.19832992
ST6GALNAC5	0.01127646	1.14948	1.032062	1.280256498
NRXN3	0.011293801	1.099909	1.021791	1.18400008
ADAMTSL3	0.011331961	1.121585	1.026272	1.225749151
AVPR1A	0.011427141	1.17042	1.036059	1.322204596

MEIS3	0.011458045	1.195296	1.040922	1.372565002
PTGIS	0.011509394	1.100843	1.021788	1.18601388
RGAG4	0.01156194	1.170277	1.035825	1.322181071
CCL14	0.011741071	1.11252	1.023974	1.208723848
BEND6	0.011759266	1.160932	1.033685	1.303842844
CACNA1H	0.012022275	1.133408	1.027882	1.249767631
MFAP5	0.012070575	1.114928	1.024129	1.213776396
IQSEC3	0.012072428	1.158142	1.032697	1.298825642
TLL1	0.012216552	1.150807	1.031071	1.284446991
CHRM2	0.012257317	1.075951	1.016047	1.139387499
CYP1B1	0.012379346	1.108475	1.022533	1.201640092
GFRA1	0.012417887	1.093168	1.01943	1.172239033
FAP	0.012471813	1.154501	1.031452	1.292230928
NCAM1	0.012478614	1.116847	1.024099	1.21799403
OLFML3	0.012537597	1.180797	1.03637	1.345351582
FXYD1	0.012568978	1.11423	1.023492	1.213012902
RSPO3	0.012622241	1.111721	1.022946	1.208199568
CTNNA3	0.012666766	1.09849	1.020288	1.182685576
SCN2B	0.012670228	1.117896	1.024112	1.220269063
FIBIN	0.012762782	1.17608	1.035144	1.336205629
CPEB1	0.012873373	1.115841	1.023509	1.216503629
RELN	0.013035638	1.091666	1.018641	1.169926166
ANGPTL1	0.013040547	1.088475	1.018009	1.163819259
F13A1	0.013043349	1.115104	1.0232	1.215263667
GPRASP1	0.013118552	1.141261	1.028118	1.266855916
NPTXR	0.013171747	1.119126	1.023848	1.223270486
SPOCK1	0.013200872	1.110991	1.022258	1.207426755
STON1	0.013284858	1.131592	1.026102	1.247926948
ODZ3	0.013296363	1.104601	1.020942	1.195116001
HSPB6	0.013344973	1.077015	1.015545	1.142206182
COL3A1	0.0133533	1.170482	1.033258	1.325929355
COL5A1	0.013413622	1.194792	1.037587	1.375814284
IGFBP5	0.013414353	1.171108	1.033288	1.327310803
LAYN	0.013485014	1.16774	1.032575	1.320597739
ITGBL1	0.01349253	1.105788	1.020998	1.197619869
EFEMP2	0.013519143	1.214402	1.040913	1.416806857
NECAB1	0.013523017	1.122353	1.024109	1.230021853
P2RY14	0.013537553	1.152826	1.029769	1.290588737
SPARCL1	0.013631561	1.13436	1.026242	1.253868942
COL1A2	0.013659652	1.177008	1.034014	1.339776985
AOC3	0.013683578	1.124701	1.024388	1.234838122
CRISPLD2	0.013779463	1.202543	1.038385	1.392653149
FAM20A	0.013779632	1.184254	1.03514	1.354847721

PCDH7	0.013856255	1.133469	1.025833	1.252398122
CRYAB	0.013962017	1.118797	1.023014	1.22354712
EVC	0.013999193	1.165421	1.031467	1.316770674
MAP1B	0.014049019	1.154839	1.029503	1.295434296
UBE2QL1	0.014132189	1.152078	1.028905	1.289997441
WISP2	0.014243194	1.10385	1.019994	1.194600664
THBS2	0.014255434	1.133213	1.025361	1.252409623
MGP	0.014260095	1.122971	1.023493	1.232117683
CCDC80	0.014326354	1.11737	1.022407	1.221153474
KIAA1755	0.01435548	1.189491	1.035214	1.366759163
HTR2A	0.014563953	1.12698	1.023919	1.240413696
ASAM	0.014603291	1.120119	1.022647	1.226882485
FN1	0.014750751	1.145293	1.026977	1.277239441
FBLN1	0.014869906	1.119581	1.022301	1.226118964
ENOX1	0.014911108	1.150529	1.027711	1.288024157
COL10A1	0.014921907	1.094105	1.017678	1.176271636
COL6A2	0.015305283	1.190571	1.034021	1.370822462
KIAA2022	0.015343643	1.10266	1.01889	1.193318296
ANGPTL2	0.015662335	1.174952	1.030939	1.339083101
CYS1	0.015709212	1.110521	1.01997	1.209110945
DDR2	0.01580394	1.127932	1.022877	1.243777871
PGR	0.015828067	1.122616	1.021947	1.233200803
OGN	0.015927218	1.074884	1.01359	1.139884116
NFASC	0.015953602	1.110897	1.019832	1.210093793
PRDM6	0.016044622	1.150178	1.02637	1.288921441
INMT	0.016081207	1.135685	1.023915	1.259655134
FLJ42709	0.016152871	1.161865	1.028174	1.312940495
CILP	0.01621039	1.083643	1.014952	1.15698237
BAI3	0.01621857	1.113353	1.02003	1.215215208
LOC100192378	0.016257902	1.133887	1.02344	1.256252669
ZCCHC24	0.016271708	1.156484	1.027155	1.302097001
CALD1	0.016288118	1.137903	1.024077	1.26438034
DSEL	0.016403332	1.150783	1.026075	1.29064732
TMEM47	0.016451272	1.169084	1.028988	1.328253764
SMOC2	0.016470536	1.112148	1.019618	1.213074903
LIMS2	0.016724894	1.134346	1.02306	1.257738153
PLA2G5	0.016770977	1.134001	1.022959	1.25709629
GLI1	0.016890089	1.14287	1.024277	1.275194626
EFS	0.016999844	1.167396	1.028061	1.325616649
FOLR2	0.017081434	1.143787	1.024228	1.277301898
ST6GAL2	0.017123556	1.111223	1.018937	1.211867111
GAS1	0.017160002	1.110335	1.018763	1.21013738
PRKG1	0.017243543	1.154078	1.025688	1.298538684

SORCS1	0.01731899	1.098284	1.016679	1.186440213
HSPB8	0.017401575	1.10574	1.017831	1.201242632
SIGLEC6	0.017507192	1.118662	1.019824	1.22707873
THBS4	0.017521721	1.060345	1.010304	1.112863912
LRRC4C	0.017575499	1.118402	1.019725	1.226627256
HIC1	0.017691058	1.203004	1.032627	1.401491596
GDF10	0.017767036	1.103446	1.017192	1.197014606
SFRP2	0.018155469	1.07819	1.012908	1.147679945
PDZRN4	0.018174605	1.080364	1.013245	1.151929494
DARC	0.018200795	1.098811	1.016151	1.188193832
ATP1A2	0.018267283	1.070872	1.011678	1.133530191
WSCD2	0.018585151	1.080391	1.01302	1.152241364
BICC1	0.018590208	1.135965	1.021552	1.263191796
SFRP4	0.018661294	1.081876	1.01321	1.155195237
NEGR1	0.018743303	1.117161	1.018582	1.225280405
SGCA	0.018758262	1.093909	1.015019	1.178931324
CNN1	0.018848113	1.072331	1.011621	1.136684251
ITGA8	0.018866297	1.104868	1.016623	1.200772616
GPC6	0.019111033	1.168329	1.02578	1.330687942
SELP	0.019145799	1.112997	1.017643	1.217286155
SRPX	0.019205072	1.123492	1.019156	1.238508654
ADORA3	0.019378188	1.167272	1.025334	1.328858111
OLFML1	0.019396706	1.212222	1.031595	1.42447567
GFPT2	0.019460575	1.133028	1.020335	1.258167698
KCNMA1	0.019460841	1.082873	1.012916	1.157661065
CFH	0.019468852	1.178285	1.026787	1.352135946
HGF	0.019630071	1.14396	1.021755	1.280781668
C15orf59	0.019860447	1.134867	1.020248	1.262362333
NHSL2	0.019879883	1.127743	1.019214	1.247828441
MAPK10	0.020251163	1.129713	1.019183	1.252230063
PRIMA1	0.02031674	1.080728	1.012134	1.153971375
AOX1	0.020480577	1.096765	1.014349	1.185877753
PTCH2	0.020593729	1.112284	1.016468	1.217132755
LMOD1	0.020648614	1.086201	1.012742	1.164989315
FAM180A	0.020721439	1.112688	1.016432	1.218058329
ADAMTSL1	0.020766587	1.146557	1.021053	1.287487033
EVC2	0.020792626	1.14997	1.021491	1.29460984
C10orf72	0.021089964	1.155356	1.021926	1.306207712
FMO2	0.021173723	1.098706	1.014186	1.190269268
LUM	0.021181326	1.174361	1.024335	1.34635989
COMP	0.021285291	1.069112	1.010001	1.131682321
ZDHHC15	0.021292904	1.131113	1.018509	1.256165712
SLIT3	0.021392336	1.1285	1.018078	1.250898666

ODZ4	0.021488893	1.134909	1.018852	1.26418631
BVES	0.021514246	1.114293	1.016081	1.221998676
RAB31	0.021528476	1.211787	1.028703	1.427454273
RBPMS2	0.021574874	1.101504	1.014314	1.196188153
TSHZ3	0.022132233	1.177453	1.023703	1.354295997
SPON1	0.022756606	1.109289	1.014566	1.212855604
PLIN4	0.022777088	1.073592	1.00994	1.141256351
FBLN2	0.022788535	1.128571	1.016981	1.252404295
GRIN2A	0.022833841	1.084309	1.011309	1.162578574
TLR7	0.022940939	1.142451	1.018583	1.281382637
HS3ST2	0.023322519	1.119423	1.015445	1.234047657
PLP1	0.023358965	1.076367	1.010031	1.147060249
PRICKLE2	0.023713198	1.133361	1.016844	1.263228754
FGF14	0.023854502	1.109335	1.013849	1.213813892
FAM19A4	0.023915798	1.090811	1.011555	1.176276117
GPR1	0.023976354	1.133065	1.016602	1.262870928
ABI3BP	0.024092047	1.094068	1.011855	1.182960006
TMEM130	0.024299177	1.102605	1.012761	1.200419836
ISM1	0.024556125	1.096402	1.011873	1.187991549
KLF17	0.024565694	1.113694	1.0139	1.223309414
FAM198A	0.024695489	1.113653	1.013809	1.223329153
PLXNC1	0.024707995	1.138809	1.016688	1.275598397
TGFB3	0.024899574	1.159357	1.018831	1.31926496
PAK3	0.025138533	1.11187	1.013315	1.220010433
TMEM35	0.025188738	1.087826	1.01053	1.171034218
DCN	0.025310383	1.150387	1.017481	1.300652527
MEOX2	0.02568685	1.094205	1.010994	1.184265195
C13orf33	0.026006271	1.130044	1.014724	1.258468905
CDH19	0.026060095	1.083632	1.009623	1.163065492
PNMAL1	0.026212438	1.103528	1.011727	1.203659462
TNS1	0.02629168	1.118007	1.013236	1.233612402
ATP8B2	0.026362851	1.170304	1.018644	1.344543545
ADAMTS2	0.026507796	1.142366	1.015642	1.284901818
TMEM119	0.026522832	1.147845	1.016197	1.296548485
PRND	0.026560944	1.111921	1.012415	1.221206905
MYLK	0.026564654	1.096792	1.010801	1.190098425
COL1A1	0.026650934	1.141384	1.015428	1.282963049
DOK5	0.026895636	1.148109	1.015919	1.29749903
SCN7A	0.02693475	1.073017	1.008075	1.142141389
CPA3	0.027085134	1.110354	1.011926	1.218355702
MRAS	0.027096027	1.187178	1.019611	1.382284526
BMPER	0.027381669	1.106916	1.011395	1.211459521
GNB4	0.027413814	1.19626	1.020156	1.402764608

LILRA4	0.027593607	1.111749	1.011756	1.221624215
FAM43B	0.027720584	1.130648	1.013549	1.261276925
AGTR1	0.027768823	1.079029	1.00835	1.154662315
C1orf95	0.027788582	1.086274	1.009079	1.169374498
JAKMIP2	0.027803775	1.124269	1.012864	1.247926421
NBLA00301	0.02784287	1.064644	1.006845	1.125760056
COL6A3	0.028115785	1.170644	1.017057	1.347423562
GLRB	0.02817058	1.123462	1.012539	1.246537094
PLAC9	0.028380077	1.12646	1.012685	1.253018657
ZNF423	0.028391802	1.142529	1.014195	1.287102185
PYGM	0.028469372	1.091219	1.009239	1.179859023
CADM3	0.028527128	1.077362	1.007856	1.15165994
DES	0.028758143	1.050607	1.005134	1.098137386
ADCY5	0.028835201	1.0791	1.007894	1.155335918
TM6SF1	0.030047258	1.197714	1.017575	1.409743149
ACTC1	0.030151155	1.092358	1.008516	1.18316946
MRGPRF	0.030202781	1.100375	1.009197	1.199789922
ARSI	0.03023975	1.12284	1.011128	1.246894386
FHL1	0.030396572	1.094106	1.00855	1.186920748
KCNK3	0.030794457	1.091157	1.008101	1.181054953
CCL21	0.030943966	1.083299	1.007362	1.164960364
NRXN2	0.031281323	1.094683	1.008162	1.188628478
FAM124B	0.031357233	1.162379	1.013551	1.333060094
MRVI1	0.031487345	1.122442	1.010304	1.247027014
PCDHGB6	0.031843691	1.132688	1.010879	1.269175175
PCDHGA12	0.031874253	1.138129	1.011279	1.280890536
ACTN2	0.032347394	1.075062	1.006111	1.148739494
ADCY2	0.032405772	1.089048	1.007179	1.1775718
DKK2	0.032422653	1.123563	1.009807	1.250132561
DACT3	0.032484362	1.107661	1.008569	1.216488668
STMN2	0.032503051	1.087797	1.007038	1.175031991
RUNX1T1	0.032643366	1.115749	1.009088	1.233683657
C9orf4	0.032767049	1.085358	1.006735	1.170119963
ANK2	0.033105149	1.096838	1.007438	1.194170342
ADAMTS10	0.033140778	1.155384	1.01162	1.319578391
C7orf58	0.033261851	1.106198	1.008042	1.213911345
SLITRK5	0.033265235	1.079824	1.006112	1.158937314
SYNPO2	0.033495905	1.06913	1.005237	1.137083997
HRH2	0.034085237	1.168513	1.011761	1.34954965
BNC2	0.03408567	1.111856	1.007992	1.226422583
NBEA	0.034158728	1.090177	1.00647	1.180846053
GSTM5	0.034161688	1.113649	1.008072	1.230284241
BMP3	0.034299194	1.063641	1.004575	1.126180311

LTBP1	0.034465679	1.152806	1.010452	1.315215384
CYP7B1	0.035064017	1.133413	1.008812	1.273402926
SFRP1	0.035222462	1.071497	1.004794	1.142629384
LMO3	0.035348668	1.091285	1.006011	1.183785662
CDH23	0.035484848	1.111188	1.007186	1.225928398
DPT	0.035576436	1.084807	1.005506	1.170361496
GEFT	0.036495613	1.119417	1.007113	1.244245404
FGF7	0.036646038	1.108686	1.006425	1.221336532
LOC100126784	0.036655818	1.109532	1.006468	1.223149588
PCDH9	0.037018724	1.085495	1.004953	1.172493216
C2orf40	0.037026938	1.059961	1.003511	1.119586607
MS4A2	0.037340653	1.109481	1.006109	1.223473409
HPGDS	0.037883008	1.120686	1.006395	1.247956785
GNAO1	0.038047084	1.088782	1.004701	1.179899841
TMEM100	0.038455533	1.092941	1.004734	1.188891327
ZNF835	0.038579629	1.11077	1.005534	1.227020048
FAM129A	0.038769535	1.114996	1.005634	1.236250965
ZNF354C	0.039198746	1.129577	1.006053	1.268268045
PDZRN3	0.039316219	1.1158	1.005379	1.238349189
DLG2	0.039363527	1.094969	1.004431	1.193667375
ADAMTS8	0.039391959	1.087673	1.004092	1.178212145
PLXNA4	0.039793107	1.093373	1.004174	1.190494999
ZNF385D	0.040005312	1.104446	1.004544	1.214283712
NOG	0.040136721	1.110718	1.004737	1.227877167
NTN1	0.040412251	1.105573	1.004395	1.216943493
SIRPB2	0.040910954	1.157102	1.006047	1.330838137
AFF3	0.041242638	1.090552	1.003451	1.185212653
FRZB	0.041278443	1.124902	1.004668	1.25952461
CYBRD1	0.041380627	1.138182	1.005072	1.288919585
CHRNA3	0.041884425	1.07609	1.002696	1.154856205
SCRG1	0.042051932	1.062634	1.002185	1.126729252
JAM2	0.042738819	1.122358	1.003784	1.254939231
EML1	0.042871012	1.128225	1.00388	1.267971645
MYOCD	0.042877641	1.078809	1.002436	1.161001966
REEP2	0.043281004	1.097323	1.002808	1.20074586
SULF1	0.043528999	1.119369	1.003281	1.248889046
NRXN1	0.044119475	1.076411	1.00194	1.1564161
PRUNE2	0.044177901	1.069317	1.001748	1.141444378
MFAP4	0.044506134	1.096784	1.002271	1.200209316
HAS1	0.044616463	1.090074	1.002076	1.185798785
RGMA	0.045203028	1.077357	1.001593	1.158852285
PTGFR	0.045235729	1.112421	1.002263	1.234687072
ASB5	0.045286539	1.058871	1.001201	1.119863015

EBF3	0.045327907	1.129823	1.002542	1.273264104
TNC	0.045480665	1.099943	1.001917	1.207560351
GLI3	0.0455955	1.121754	1.002253	1.255503816
HAND2	0.045644915	1.058962	1.00111	1.120158335
NDN	0.04608805	1.134555	1.002194	1.284396735
TMEM90B	0.046144199	1.122009	1.001971	1.256427869
ACTG2	0.046368177	1.059147	1.000925	1.120755
FMO1	0.046544353	1.095093	1.001391	1.197562994
CORIN	0.047218242	1.112296	1.001308	1.235586683
COPZ2	0.047759782	1.137267	1.00127	1.291736174
NLGN1	0.047994438	1.082604	1.000701	1.171211609
PPP1R1A	0.048260014	1.06515	1.000483	1.133995977
MYH11	0.048280133	1.057431	1.000422	1.117689065
GUCY1A3	0.049432723	1.118365	1.000278	1.250392506
PPAPDC3	0.049529824	1.134159	1.000259	1.285983055
NCAM2	0.049640935	1.083044	1.000125	1.172837679
MEF2C	0.049740847	1.15961	1.000168	1.344468795
KERA	0.049774532	1.097614	1.000092	1.20464529

Supplementary Table 3.

Univariate Cox regression model verification of genes both significant in TCGA and GSE26253.

Sig gene name	p.value	HR	Low 95%CI	High 95%CI
GALNTL1	0.022205097	1.09620301	1.013215792	1.185987279
MRAS	0.045428766	1.109764654	1.002120881	1.228971086
FAP	0.007528325	1.122417275	1.031269928	1.221620552
GLI1	0.049496161	1.127429066	1.000264345	1.27076038
HIC1	0.01396333	1.127863955	1.024688007	1.241428701
INMT	0.04701597	1.127933608	1.001588862	1.270216027
TNS1	0.016548873	1.131511578	1.022764767	1.251821038
FAM19A5	0.006441892	1.132280609	1.035473217	1.238138616
ADIPOQ	0.037311091	1.134034145	1.007419578	1.276561892
SPOCK1	0.034774705	1.136023763	1.009164892	1.278829655
LRCH2	0.03085733	1.13653604	1.011863667	1.276569377
TNN	0.009395689	1.141063079	1.03291136	1.260538901
MXRA8	0.014584973	1.142753091	1.026713864	1.271907075
NTN1	0.012861788	1.142942375	1.028742895	1.269818999
HAND2	0.03160617	1.146048365	1.012084365	1.297744437
SLC22A17	0.019787363	1.146358878	1.021947395	1.285916167
MOXD1	0.016081488	1.150335898	1.026355497	1.289292729
MN1	0.027617393	1.151629457	1.015678128	1.305778249
TGFB3	0.00483978	1.158037347	1.045670308	1.282479275
LHFP	0.027153137	1.161356512	1.017026082	1.326169476
SLIT2	0.033628899	1.163005706	1.011763314	1.336856411

C7	0.01339622	1.16380558	1.031973289	1.312479154
HS3ST2	0.024150206	1.165451721	1.020217999	1.331360273
SCRG1	0.005978889	1.165888858	1.045036075	1.300717614
C1QTNF7	0.017139439	1.167566134	1.027922306	1.326180654
PREX2	0.00060116	1.168491622	1.069043536	1.277190896
RERG	0.011501327	1.168897989	1.035643428	1.319298199
CPE	0.006365194	1.172434155	1.045821131	1.314375669
SULF1	0.009313748	1.180017125	1.041605349	1.336821492
TMEM119	0.00103597	1.182145094	1.069680111	1.306434522
MAP1B	0.025450562	1.18586	1.02116462	1.377117766
FLRT2	0.016544449	1.190724717	1.032319373	1.373436737
PLXDC2	0.039099369	1.19141513	1.008797139	1.407091632
FSTL1	0.036847182	1.191439403	1.010755293	1.404422871
SVEP1	0.010213603	1.194805333	1.043070683	1.368612701
BICC1	0.014828002	1.195200677	1.035493552	1.379539888
ISLR2	0.001375281	1.196591643	1.072017303	1.33564221
MEOX2	0.002036594	1.200133592	1.068786219	1.347622764
APOD	0.001092491	1.201163798	1.07603287	1.340846093
PDE1A	0.010196583	1.203897971	1.044979236	1.386984808
COMP	4.31E-05	1.204699329	1.101855149	1.317142707
CYP1B1	0.010851221	1.207540124	1.044450863	1.396095501
BOC	0.009754129	1.220730297	1.04937204	1.420070673
GLT8D2	0.012291962	1.221091413	1.044330593	1.427770334
NRXN2	0.000974079	1.22452435	1.085645232	1.381169316
OLFML2B	0.000368254	1.22613884	1.096022022	1.371702779
NCAM2	0.029980678	1.233781476	1.020573159	1.491531222
CDH11	0.001959655	1.235422721	1.080676617	1.412327494
LRRC32	0.030927985	1.238965933	1.019858274	1.505146963
RGS4	0.000603431	1.239716725	1.096471562	1.401675713
MAP6	0.014690621	1.239877208	1.043202008	1.473631646
EFEMP1	0.004637576	1.247326438	1.070373435	1.453533123
HGF	0.028436921	1.258529989	1.024563237	1.545924816
CDO1	0.006052665	1.259750425	1.068262296	1.485563179
FMO2	9.81E-07	1.262049095	1.149763429	1.385300557
SFRP2	0.001290532	1.269535762	1.097792282	1.468147551
FAM124B	0.000754645	1.269953406	1.105111874	1.459383156
NOX4	0.000129726	1.27306606	1.12499903	1.440620968
FNDC1	0.000292436	1.275913348	1.118296583	1.45574519
CST2	2.59E-05	1.289074365	1.145233183	1.450981986
DACT1	9.13E-05	1.29605488	1.138170057	1.475841191
MAPK10	0.00461218	1.296901502	1.083409296	1.552463611
F13A1	0.00137296	1.300681689	1.107257401	1.527894827
THBS2	0.016814637	1.303123024	1.0488624	1.619020394

THBS4	6.59E-07	1.320862671	1.183653161	1.473977558
RUNX1T1	0.000120586	1.329598118	1.149882814	1.537401145
SCARF2	0.000677288	1.356051515	1.137609234	1.616438805
OMD	3.31E-05	1.360158823	1.176283845	1.572776869
ELN	0.004932745	1.381510866	1.102824087	1.730622586
ASPN	0.000355394	1.390401842	1.16032757	1.666096138
COL1A1	0.024961273	1.390873198	1.042377847	1.855880052
FBLN5	0.005177808	1.411779016	1.108594945	1.797879376
PRICKLE1	0.002616435	1.505195171	1.153281802	1.964491678
COL8A1	1.20E-06	1.536975938	1.29216164	1.828173009
AEBP1	0.003765841	1.540384805	1.14999197	2.063306015
DCN	0.026318219	1.598495866	1.05677256	2.417917657
COL10A1	8.79E-07	1.657157586	1.354951771	2.026766799
ANTXR1	6.44E-05	1.698088849	1.30970807	2.201640048
MGP	9.96E-05	1.891679751	1.372179856	2.607859505
PODN	0.001767424	2.010031854	1.297611738	3.113587782
BGN	1.83E-05	5.841759195	2.605760536	13.09642618

Supplementary information

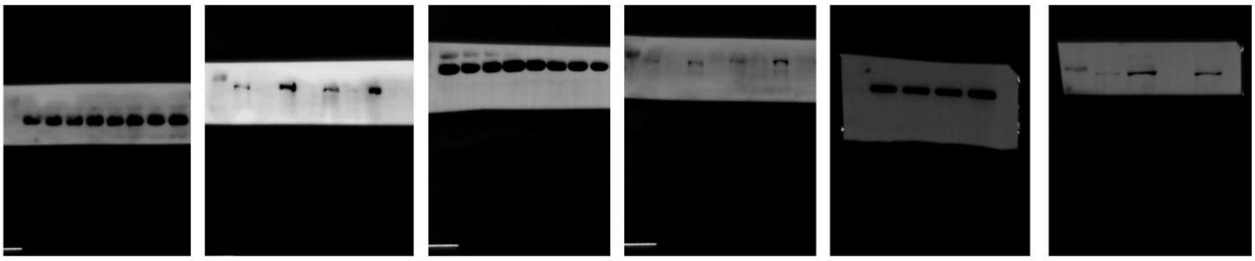

Figure5-1 β-actin   Figure5-1 ADAMTS12   Figure5-2 β-actin   Figure5-2 ADAMTS12   Figure9 β-actin   Figure9 ADAMTS12

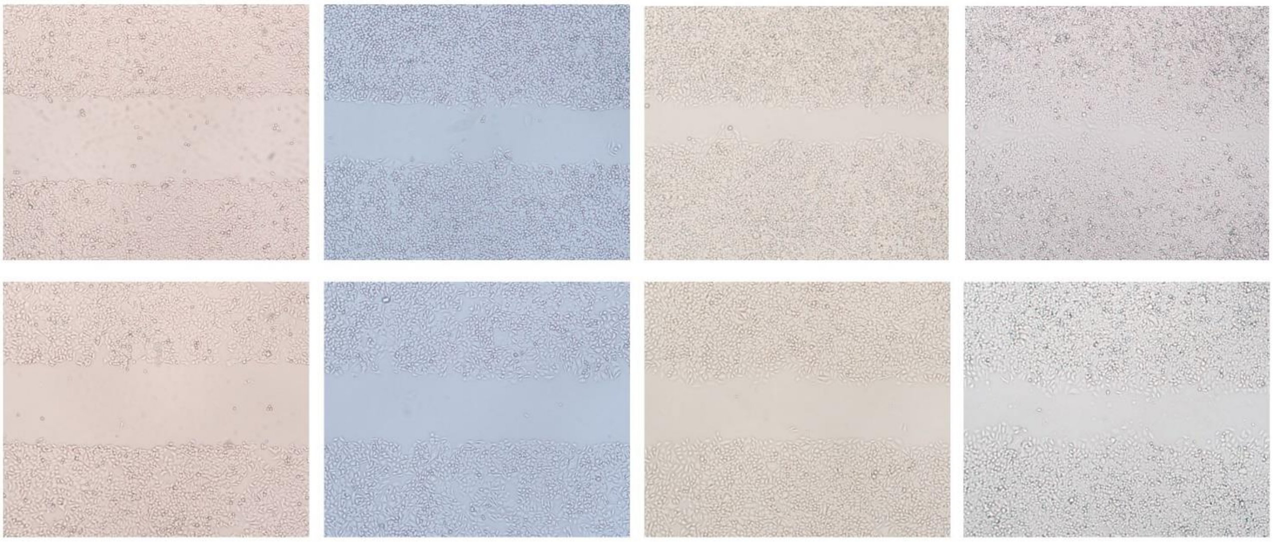

Supplement: Supplementary file 1 — Supplementary Information. [file 41598_2021_90330_MOESM1_ESM.pdf]
